# Supplementary material for: New Approaches for Escherichia coli Genotyping
Source: Pathogens. 2020 Jan 21;9(2):73. doi: 10.3390/pathogens9020073 (PMC7168681; doi:10.3390/pathogens9020073)
Supplement: Supplementary file 1 [file pathogens-09-00073-s001.pdf]

```
                             *        20         *        40         *        60         *        80         *        100
H21_AIHL01000060.1: ggtcaggcgattgctaaccgttttacggcaaatattaaaggtctgacccaggcttcccgtaacgcgaatgatggtatttctgttgcgcagaccactgaag :  100
H8_AJ865465.1     : ggtcaggcgattgctaaccgttttacggcaaatattaaaggtctgacccaggcttcccgtaacgcaaatgatggtatttctgttgcgcagaccactgaag :  100
H40_AJ884568.1    : ggtcaggcgattgctaaccgttttacggcaaatattaaaggtctgacccaggcttcccgtaacgcgaatgatggtatttctgttgcgcagaccactgaag :  100
H11_AY337465.1    : ggtcaggcgattgctaaccgttttacagcaaatattaaaggtctgactcaggcttcccgtaacgcgaatgatggtatttctgttgcgcagaccactgaag :  100
H27_AM231154.2    : ggtcaggcgattgctaaccgttttacggcaaatattaaaggtctgactcaggcttcccgtaacgcgaatgatggtatttctgttgcgcagactactgaag :  100
H2_AIHA01000023.1 : ggtcaggcgattgctaaccgttttacagcaaatattaaaggtctgactcaggcttcccgtaacgcgaatgatggtatttctgttgcgcagaccactgaag :  100
H16_AY337475.1    : ggtcaggcgattgctaaccgttttacagcaaatattaaaggtctgactcaggcttcccgtaacgcgaatgatggtatttctgttgcgcagaccactgaag :  100
H3_AB128916.1     : ggccaggcgattgctaaccgcttcacatctaacatcaaggggttaagccaggcatctcgtaatgctaatgatggtatctcactggcacagaccactgaag :  100
H54_AB128918.1    : ggtcaggcgattgctaaccgtttcaccgcgaacatcaaaggtctgactcaggcttctcgtaacgccaatgatggtatttctgttgctcagaccacggaag :  100
H53_AB128917.1    : ggtcaggcgattgctaaccgctttacttcgaacatcaaaggtctgactcaggcttctcgtaacgccaatgacggtatctccatcgcacagacgactgaag :  100
H47_EF392694.1    : ggtcaagcgattgctaaccgctttacctccaacatcaaaggtctgacccaggcatctcgtaacgctaatgatggtatttctgtagctcaaactactgaag :  100
H35_EF392692.1    : ggtcaggcgattgctaaccgctttacttcaaacattaagggcctgacccaggcatctcgtaatgccaatgatgggatttctgtagctcagacgaccgaag :  100
H43_AIGA01000038.1: ggtcaggcgattgctaaccgttttacttctaatattaaaggcctgactcaggctgcacgtaacgccaatgacggtatttctctggcgcagaccactgaag :  100
H17_CP002291.1    : ggccaggcgattgctaaccgtttcacctctaacattaaaggtctgactcaggcagctcgtaacgctaatgatggtatctccgttgcgcagaccactgaag :  100
H5_AY249990.1     : ggccaggcgattgctaaccgcttcacttctaacatcaaaggtctgactcaggccgcacgtaacgccaacgacggtatctctctggcgcagaccactgaag :  100
H56_AY250029.1    : ggtcaggcgatagctaaccgtttcacctctaacattaaaggcctgactcaggctgcgcgtaacgccaacgacggtatttctctggcgcagaccacagaag :  100
H42_AY250021.1    : ggccaggcgattgctaaccgcttcacttctaacatcaaaggtctgactcaggccgcacgtaacgccaacgacggtatctctctggcgcagaccactgaag :  100
H29_AY250012.1    : ggccaggcgattgctaaccgcttcacttctaacatcaaaggtctgactcaggccgcacgtaacgccaacgacggtatctctctggcgcagaccactgaag :  100
H38_AY250018.1    : ggccaggcgattgctaaccgcttcacttctaacatcaaaggtctgactcaggccgcacgtaacgccaacgacggtatctctctggcgcagaccactgaag :  100
H33_AY250015.1    : ggccaggcgattgctaaccgcttcacttctaacatcaaaggtctgactcaggccgcacgtaacgccaacgacggtatctctctggcgcagaccactgaag :  100
H39_AY250019.1    : ggccaggcgattgctaaccgcttcacttctaacatcaaaggtctgactcaggctgcacgtaacgccaatgacggtatttctctagcacagacagcggaag :  100
H52_AY250028.1    : ggtcaggcgattgctaaccgttttacttctaacattaaaggcctgactcaggctgcacgtaacgccaacgacggtatttctgttgcacagaccactgaag :  100
H4_AJ536600.1     : ggccaggcgattgctaaccgctttacttctaacatcaaaggtctgactcaggccgcacgtaacgccaacgacggtatttctctggcgcagacggctgaag :  100
H25_AGSG01000116.1: ggccaggcgattgctaaccgcttcacttctaacatcaaaggtctgactcaggccgcacgtaacgccaacgacggtatttctctggcgcagacggctgaag :  100
H10_AY249995.1    : ggtcaggcgattgctaaccgttttacttctaacattaaaggcctgactcaggctgcacgtaacgccaacgacggtatttctgttgcgcagaccaccgaag :  100
H24_AY250006.1    : ggccaggcgattgctaaccgcttcacttctaacatcaaaggtctgactcaggccgcacgtaacgccaacgacggtatttctctggcgcagaccactgaag :  100
H48_AY250025.1    : ggtcaggcgattgctaaccgtttcacctctaacattaaaggcctgactcaggcggcccgtaacgccaacgacggtatctccgttgcgcagaccaccgaag :  100
H1_AB028471.1     : ggtcaggcgattgctaaccgttttacttctaacattaaaggcctgactcaggctgcacgtaacgccaacgacggtatttctgttgcacagaccactgaag :  100
H12_AY337471.1    : ggtcaggcgattgctaaccgttttacttctaacattaaaggcctgactcaggctgcacgtaacgccaacgacggtatttccgttgcgcagaccaccgaag :  100
H51_AY250027.1    : ggtcaggcgattgctaaccgtttcacctctaacattaaaggcctgactcaggctgcacgtaacgctaacgatggtatctctctggcgcagaccactgaag :  100
H45_AY250023.1    : ggtcaggcgattgctaaccgttttacctctaacattaaaggtctgactcaggctgcacgtaacgccaacgacggtatttctgttgcacagaccactgaag :  100
H49_AY250026.1    : ggtcaggcgattgctaaccgttttacttctaacattaaaggcctgactcaggctgcacgtaacgccaacgacggtatttctgttgcgcagaccaccgaag :  100
H23_AB028476.1    : ggtcaggcgattgctaaccgttttacttctaacattaaaggcctgactcaggcggcacgtaacgccaacgacggtatctctctggcgcagaccaccgaag :  100
H7_AY337468.1     : ggtcaggcgattgctaaccgttttacttctaacattaaaggcctgactcaggcggcccgtaacgccaacgacggtatttctgttgcgcagaccaccgaag :  100
H6_AY249991.1     : ggtcaggcgattgctaaccgttttacttctaacattaaaggcctgactcaggctgcacgtaacgccaacgacggtatttctgttgcgcagaccaccgaag :  100
H18_AY250001.1    : ggtcaggcgattgctaaccgttttacttctaatattaaaggcctgactcaggctgcacgtaacgccaatgacggtatttctgttgcacagaccactgaag :  100
H34_AY250016.1    : ggtcaggcgattgctaaccgttttacttctaacattaaaggcctgactcaggctgcacgtaacgccaatgacggtatttctgttgcacagaccactgaag :  100
H14_AY249998.1    : ggtcaggcgattgctaaccgttttacttctaacattaaaggcctgactcaggctgcacgtaacgccaacgacggtatttccgttgcgcagaccactgaag :  100
H15_AY249999.1    : ggtcaggcgattgctaaccgttttacttctaacattaaaggcctgactcaggctgcacgtaacgccaacgacggtatttctgttgcacagaccactgaag :  100
H20_AY250003.1    : ggtcaggcgattgctaaccgttttacttctaacattaaaggcctgactcaggcggcccgtaacgccaacgacggtatttctgttgcgcagaccaccgaag :  100
H31_AY250013.1    : ggtcaggcgattgctaaccgttttacttctaacattaaaggcctgactcaggctgcacgtaacgccaacgacggtatttctgttgcgcagaccactgaag :  100
H28_AY250010.1    : ggtcaggcgattgctaaccgttttacttctaacattaaaggcctgactcaggctgcacgtaacgccaacgatggtatttctgttgcacagaccactgaag :  100
H46_AY250024.1    : ggtcaggcgattgctaaccgttttacttctaatattaaaggcctgactcaggctgcacgtaacgccaatgacggtatttctgttgcacagaccactgaag :  100
H44_AB269770.1    : ggccaggcgattgctaaccgtttcacctctaacatcaaaggtctgactcaggctgcccgtaacgccaacgacggtatctccgttgcacagactactgaag :  100
```

```
H19_AY250002.1     : ggtcaggcgattgctaaccgtttacttctaacattaaaggcctgactcaggctgcacgtaacgccaacgacggtatttctgttgcgcagaccactgaag : 100
H55_AB269771.1     : ggccaggcgattgccaaccgcttcacctctaacatcaaaggtctgactcaggcagctcgtaacgccaacgacggtatctccgttgcacagaccactgaag : 100
H26_AY250008.1     : ggtcaggcgattgctaaccgtttacttctaacattaaaggcctgactcaggctgcacgtaacgccaacgacggtatttctgttgcacagaccactgaag : 100
H30_AY250011.1     : ggtcaggcgattgctaaccgtttacttctaacattaaaggcctgactcaggctgcacgtaacgccaacgacggtatttctgttgcacagaccactgaag : 100
H32_AY250014.1     : ggtcaggcgattgctaaccgtttacttctaacattaaaggcctgactcaggctgcacgtaacgccaacgacggtatttccgttgcgcagaccaccgaag : 100
H37_AY250017.1     : ggtcaggcgattgctaaccgtttacttctaacattaaaggcctgactcaggctgcacgtaacgccaacgacggtatttctgttgcgcagaccaccgaag : 100
H41_AY250020.1     : ggtcaggcgattgctaaccgtttcacctctaacattaaaggcctgactcaggctgcacgtaacgccaacgacggtatttctgttgcacagaccaccgaag : 100
H9_AY249994.1      : ggtcaggcgattgctaaccgtttacttctaacattaaaggcctgactcaggctgcacgtaacgccaacgacggtatttccgttgcacagaccactgaag : 100
H36_EF392693.1     : ggtcaggcgattgctaaccgcttcacatctaatatcaaaggtcttactcaggcttcccgaaacgccaacgatggtatctctatcgctcagaccactgaag : 100
                     GG CAgGCGATtGCtAACCG TT AC tCtAAcAT AAaGG cTgActCAGGC   C CGtAAcGCcAA GA GGtAT TCt T GC CAgACcaC GAAG

                            *       120         *       140         *       160         *       180         *       200
H21_AIHL01000060.1: gtgcgctgaatgaaattaacaacaacctgcagcgtattcgtgaactttctgttcaggcaactaacggtactaactctgacagcgatctttcttctatcca : 200
H8_AJ865465.1      : gtgcgctgaatgaaattaacaacaacctgcagcgtattcgtgaactttctgttcaggcaactaacggtactaactctgacagcgatctttcttctatcca : 200
H40_AJ884568.1     : gtgcgctgaatgaaattaacaacaacctgcagcgtattcgtgaactttctgttcaggcaactaacggtactaactctgacagcgatctttcttctatcca : 200
H11_AY337465.1     : gtgcgctgaatgaaattaacaacaacctgcagcgtgtacgtgaactgactgttcaggcaactaacggtactaactctgacagcgatctttcttctatcca : 200
H27_AM231154.2     : gtgcgctgaatgaaattaacaacaacctgcagcgtgtacgtgaactgactgttcaggcaactaacggtactaactctgacagcgatctttcttctattca : 200
H2_AIHA01000023.1 : gtgcgctgaatgaaattaacaacaacctgcagcgtattcgtgaactttctgttcaggcaactaacggtactaactctgacagtgacctgacctccatcca : 200
H16_AY337475.1     : gtgcgctttctgaaatcaacaataacttacagcgtattcgtgaattgtcagtacaggccactaatggtacaaactctgactccgacctgaattcaattca : 200
H3_AB128916.1      : gtgcactgtcagaaatcaacaacaacctccaacgagtacgtgagcttgctgttcaggctactaacggcacgaactctcagtctgatctaaactccattca : 200
H54_AB128918.1     : gcgcactgtctgaaatcaacaataacttacaacgtgtccgtgagcttacagttcaagctactaacggtacaaattctgatagcgatttatcatcaatcca : 200
H53_AB128917.1     : gtgcactgaacgaaatcaacaataatctgcagcgtgtacgtgaactggctgttcaatctcagaatgggacaaactctgattcagatgttcaatccattca : 200
H47_EF392694.1     : gtgccctcggcgaaattaacaacaacttacaacgtattcgagagctaacggttcaagctacaaatggcactaactctcaatctgacatggaatctatcca : 200
H35_EF392692.1     : gttcattatctgaaatcaacaataacttacagcgtattcgtgagttgtcagtacaagcaactaatggctctaactctgattcagacttgtcctcgattca : 200
H43_AIGA01000038.1: gcgcactgtctgaaatcaacaacaacttgcagcgtgtgcgtgaactgaccgtacaggcgacaaccggaacgaactccgaatctgacctgtcctctatcca : 200
H17_CP002291.1     : gcgcactgtctgaaatcaacaacaacttacagcgtgttcgtgagctgacggttcaggcctctaccggtaccaactctgcttctgacctgtcttccatcca : 200
H5_AY249990.1      : gcgcgctgtctgaaatcaacaacaacttgcagcgtgtgcgtgagttgaccgttcaggcgacgaccgggactaactctgattctgacctgtcttctattca : 200
H56_AY250029.1     : gtgcgttgtctgaaatcaacaacaacttgcaacgtgtgcgtgagttgaccgttcaggcgacgaccggtactaactctgattctgacctgtcatctattca : 200
H42_AY250021.1     : gcgcactgtctgaaatcaacaacaacttgcagcgtgttcgtgaactgaccgttcaggccactaccggtactaactctgattctgacctgtcttcaatcca : 200
H29_AY250012.1     : gcgcactgtctgaaatcaacaacaacttgcagcgtgttcgtgagctgaccgttcaggccactaccggtactaactctgattctgacctgtcttcaatcca : 200
H38_AY250018.1     : gcgcactgtctgaaatcaacaacaacttgcagcgtgttcgtgaactgaccgttcaggccactaccggtactaactctgattctgacctgtcttcaatcca : 200
H33_AY250015.1     : gcgcactgtctgaaatcaacaacaacttgcagcgtgtgcgtgagttgactgttcaggcgacgaccgggactaactctgattctgacctgtcttctattca : 200
H39_AY250019.1     : gcgcgctgtcagagattaacaacaacttgcagcgtgtgcgtgagttgaccgtgcaggcaaccactggtaccaactctgattccgatctctcttctattca : 200
H52_AY250028.1     : gcgcgctgtccgaaatcaacaacaacttacagcgtattcgtgaactgacggttcaggcttctaccgggactaactctgattcggatctggactccattca : 200
H4_AJ536600.1      : gcgcgctgtcagagattaacaacaacttgcagcgtattcgtgaactgaccgttcaggcctctaccggcacgaactctgattccgacctgtcttctattca : 200
H25_AGSG01000116.1: gcgcgctgtcagagattaacaacaacttgcagcgtattcgtgaactgaccgttcaggcctctaccggcacgaactctgattccgacctgtcttctattca : 200
H10_AY249995.1     : gcgcgctgtccgaaattaacaacaacttacagcgtgtgcgtgagctgactgttcaggcgaccaccggtactaactctgagtctgacctgtcttctatcca : 200
H24_AY250006.1     : gcgcgctgtctgagattaacaacaacttgcagcgtgtgcgtgagttgactgtacaggcgacgaccgggactaactctgattctgacctgtcttctatcca : 200
H48_AY250025.1     : gcgcgctgtccgaaatcaacaacaacttacagcgtgtgcgtgaactgacggtacaggccactaccggtactaactctgagtctgatctgtcttctatcca : 200
H1_AB028471.1      : gcgcgctgtccgaaatcaacaacaacttacagcgtatccgtgagctgacggttcaggcttctaccgggactaactctgattcggatctggactccattca : 200
H12_AY337471.1     : gcgcgctgtccgaaatcaacaacaacttacagcgtatccgtgaactgacggttcaggcttctaccgggactaactccgattcggatctggactccattca : 200
H51_AY250027.1     : gcgcactgtctgagattaacaacaacttacaacgtgtgcgtgagttgactgtacaggcgaccaccggtactaactctgattctgacctggcttctattca : 200
H45_AY250023.1     : gcgcgctgtccgaaatcaacaacaacttacagcgtatccgtgaactgacggttcaggcttctaccgggactaactccgattcggatctggactccattca : 200
H49_AY250026.1     : gcgcgctgtctgaaatcaacaacaacttacagcgtattcgtgaactgacggttcaggcttctaccgggactaactctgattcggatctggactccattca : 200
H23_AB028476.1     : gtgcgctgtctgaaatcaacaacaacttacagcgtgtacgtgaactgaccgttcaggcaaccaccggtactaactccgactccgacctggcttctattca : 200
H7_AY337468.1      : gcgcgctgtccgaaatcaacaacaacttacagcgtattcgtgaactgacggttcaggccactacagggactaactccgattctgacctggactccatcca : 200
```

```
H6_AY249991.1      : gcgcgctgtccgaaattaacaacaacttacagcgtattcgtgaactgacggttcaggcttctaccgggactaactctgattcggatctggactccattca : 200
H18_AY250001.1     : gcgcgctgtccgaaatcaacaacaacttacagcgtattcgtgaactgacggttcaggccactacagggactaactccgattctgacctggactccatcca : 200
H34_AY250016.1     : gcgcgctgtccgaaatcaacaacaacttacagcgtattcgtgaactgacggttcaggcttctaccgggactaactctgattcggatctggactccattca : 200
H14_AY249998.1     : gtgcgctgtccgaaatcaacaacaacttacagcgtattcgtgagctgacggttcaggcttctaccgggactaactccgattctgacctggactccatcca : 200
H15_AY249999.1     : gcgcgctgtccgaaatcaacaacaacttacagcgtgtgcgtgaactgaccgttcaggcaaccaccggtaccaactcccagtctgacctggactctatcca : 200
H20_AY250003.1     : gcgcgctgtccgaaattaacaacaacttacagcgtgtgcgtgagctgactgttcaggcgaccaccggtaccaactcccagtctgatctggactctatcca : 200
H31_AY250013.1     : gcgcgctgtccgaaatcaacaacaacttacagcgtatccgtgaactgacggttcaggcttctaccgggactaactccgattcggatctggactccattca : 200
H28_AY250010.1     : gcgcgctgtccgaaatcaacaacaacttacagcgtatccgtgaactgacggttcaggcttctaccgggactaactccgattcggatctggactccattca : 200
H46_AY250024.1     : gcgcgctgtccgaaatcaacaacaacttacagcgtgtgcgtgaactgaccgttcaggcgaccaccggtaccaactcccagtctgatctggactctatcca : 200
H44_AB269770.1     : gcgcactgtctgaaatcaacaacaacttacagcgtatccgtgagctgactgttcagtcttctactggcaccaactccaagtctgacctggactccatcca : 200
H19_AY250002.1     : gcgcgctgtccgaaattaacaacaacttacagcgtattcgtgaactgacggttcaggcgacgaccggaactaactccacctctgacctggactccatcca : 200
H55_AB269771.1     : gcgcactgtctgaaatcaacaacaacctgcagcgtatccgtgagctgactgttcagtcttctacgggtactaactctgaatccgatctgaactcaatcca : 200
H26_AY250008.1     : gcgcgctgtccgaaatcaacaacaacttacagcgtattcgtgaactgacggttcaggccactacagggactaactccgattctgacctggactccatcca : 200
H30_AY250011.1     : gcgcgctgtccgaaatcaacaacaacttacagcgtattcgtgaactgacggttcaggcgacgaccggaactaactccacctctgacctggactccattca : 200
H32_AY250014.1     : gcgcgctgtccgaaatcaacaacaacttacagcgtatccgtgaactgacggttcaggccactaccggtactaactccgattctgacctggactccatcca : 200
H37_AY250017.1     : gcgcgctgtccgaaatcaacaacaacttacagcgtgtgcgtgaactgaccgttcaggcaaccaccggtaccaactcccagtctgacctggactctatcca : 200
H41_AY250020.1     : gcgcgctgtccgaaatcaacaacaacttacagcgtatccgtgaactgacggttcaggcttctaccgggactaactctgattcggatctggactccattca : 200
H9_AY249994.1      : gcgcgctgtccgaaattaacaacaacttacagcgtattcgtgaactgacggttcaggcttctaccgggactaactccgattcggatctggactccattca : 200
H36_EF392693.1     : gcgcgttgtctgaaatcaacaacaacctgcagcgtgttcgtgagttgtctgtacaggcaactaacggcactaactctccgtctgacctagattctattca : 200
                     G gC cTgtc GAaAT AACAAcAActT CAgCGt T CGtGA cTgaC GTtCAggC  c A cGG aC AAcTC ga tc GA cTg   TC AT CA

                            *     220         *     240         *     260         *     280         *     300
H21_AIHL01000060.1: ggctgaaattactcaacgtctggaagaaattgaccgtgtatctgagcaaactcagtttaacggcgtgaaagtccttgctgaaataatgaaatgaaaatt : 300
H8_AJ865465.1      : ggctgaaattactcaacgtctggaagaaattgaccgtgtatctgagcaaactcagtttaacggcgtgaaagtccttgctgaaataatgaaatgaaaatt : 300
H40_AJ884568.1     : ggctgaaattactcaacgtctggaagaaattgaccgtgtatctgagcaaactcagtttaacggcgtgaaagtccttgctgaaataatgaaatgaaaatt : 300
H11_AY337465.1     : ggctgaaattactcaacgtctggaagaaattgaccgtgtatctgagcaaactcagtttaacggcgtgaaagtccttgctgaaataatgaaatgaaaatt : 300
H27_AM231154.2     : ggcagaaattactcaacgtctggaagaaattgaccgtgtatctgagcaaactcagtttaacggcgtgaaagtccttgccgaaataatgaaatgaaaatt : 300
H2_AIHA01000023.1 : gtccgaaatccagcagcgtctgagtgaaattgaccgtgtttctggtcagactcagtttaacggcgttaaagtgctggcttctgatcaggatatgactatt : 300
H16_AY337475.1     : ggatgaaattacacaacgccttagtgaaattgatcgtgtttctaaccagacacaatttaatggtgtaaaagttctggcttctgatcagactatgaaaatt : 300
H3_AB128916.1      : agacgaaattactcagcgcctgagtgaaattgatcgtgtttctcaacagactcaattcaatggtgtaaaagtattggcaactaaccaaacgatgaaaatt : 300
H54_AB128918.1     : ggcggaaataactcagcgattaaatgaaattgatcgcgtctcaggccaaacacaattcaatggtgttaaagtcttagcttctaaaaataccttacaatt : 300
H53_AB128917.1     : agaggaaattcaacaacgtttagctgaaattgaccggtttctggtcaaactcaatttaacggcgttaaggtattaacatcagactcaaaacttagcatc : 300
H47_EF392694.1     : ggctgaaattacacagcgactcgatgaaattgatcgcgtttctcaacagaccgagttcaatggtgttagtgtattaggtgaagataagactctgaaaatt : 300
H35_EF392692.1     : ggatgagattactcaacgtttgcaagaaattgatcgtgtctccaatcagactcagtttaacggcgtaaaagtattagcatctcaacagactatgaaaatt : 300
H43_AIGA01000038.1: ggacgaaatcaaatcccgtctggaagagattgaccgcgtatccggccagactcagttcaacggcgtgaatgtgctggcaaagacggcaccatgaaaatt : 300
H17_CP002291.1     : ggacgaaatcaaatcccgactgtctgaaattgaccgtgtttctagtcagactcagttcaacggcgtgaacgtactggctaaagacggcaagatgaacatc : 300
H5_AY249990.1      : ggacgaaatcaaatcccgtctggatgaaattgatcgcgtttccggtcagacccagttcaacggcgtgaatgtgctggcgaaagatggttcgatgaagatt : 300
H56_AY250029.1     : ggacgaaatcaaatcccgtctggatgagattgaccgtgtttccggtcagacccagttcaacggcgtgaatgtactggcaaaagacggttcgatgaagatt : 300
H42_AY250021.1     : ggacgaaatcaaatcccgcttggctgaaatcgatcgtgtctctggtcagacccagttcaacggcgtgaacgtgctggctaaaaacggttctctgaatatt : 300
H29_AY250012.1     : ggacgaaatcaaatcccgtctcgatgaaattgaccgcgtatccggtcagactcagttcaacggcgtgaacgtactggcaaaagataacaccatgaagatt : 300
H38_AY250018.1     : ggacgaaatcaaatcccgtctcgatgaaattgaccgcgtatccggtcagactcagttcaacggcgtgaacgtactggcaaaagatggctcgatgaaaatt : 300
H33_AY250015.1     : ggacgaaatcaaatcccgtctggatgaaattgaccgtgtttccggtcagacccagttcaacggcgtgaacgtgctggctaaaaacggttctatggcgatt : 300
H39_AY250019.1     : ggatgaaattaaatctcgtctggatgaaattgaccgcgtctctggtcagacccagtttaacggcgtgaacgtactggctaaaaacggttctatggcaatt : 300
H52_AY250028.1     : ggacgaaatcaaatcccgtctcgacgaaattgagcgcgtttccggtcagacccagttcaacggcgtgaacgtgctggcgaaagacggttcgatgaagatt : 300
H4_AJ536600.1      : ggacgaaatcaaatcccgtcttgatgaaattgaccgtgtatctggtcagacccagttcaacggtgtgaacgtgctgtcgaaaaacgattcgatgaagatt : 300
H25_AGSG01000116.1: ggacgaaatcaaatcccgtcttgatgaaattgaccgtgtatctggtcagacccagttcaacggtgtgaacgtgctgtcgaaaaacgattcgatgaagatt : 300
```

```
H10_AY249995.1     : ggacgaaatcaaatctcgcctggaagagattgatcgtgtttcaagtcagactcaatttaacggcgtgaatgttttggctaaagatgggaaaatgaacatt : 300
H24_AY250006.1     : ggatgaaatcaaatcccgtttaagcgaaattgaccgtgtatctggtcagactcagtttaacggcgtgaacgtactggctaagaatgacaccctgtctatt : 300
H48_AY250025.1     : ggacgaaattaaatcccgtctggatgaaattgaccgcgtatctggtcagacccagttcaacggcgtgaacgtgctggcaaaaaatggctccatgaaaatc : 300
H1_AB028471.1      : ggacgaaatcaaatcccgtctcgacgaaattgaccgcgtatccggtcagacccagttcaacggcgtgaacgtactggcaaaagacggttcgatgaaaatt : 300
H12_AY337471.1     : ggacgaaatcaaatcccgtctggacgaaattgaccgcgtatctggccagacccagttcaacggcgtgaacgtactggcgaaagacggttcaatgaaaatt : 300
H51_AY250027.1     : ggacgaaatcaaatcccgtttgtctgaaattgaccgcgtatccgggcagacccagttcaacggcgtgaacgtattgtctaaagatggctccctgaaaatt : 300
H45_AY250023.1     : ggacgaaatcaaatcccgtctggacgaaattgaccgcgtatccggtcaaacccagttcaacggtgtgaacgtactggcgaaagacggttcgatgaaaatt : 300
H49_AY250026.1     : ggacgaaatcaaatcccgtctggacgaaattgaccgcgtatccggtcaaacccagttcaacggtgtgaacgtactggcgaaagacggttcgatgaaaatt : 300
H23_AB028476.1     : ggacgaaatcaaatcccgtctggatgaaattgaccgcgtatctggtcagactcagttcaacggcgtgaacgtgctggcaaaagacggttccatgaaaatt : 300
H7_AY337468.1      : ggacgaaatcaaatctcgtcttgatgaaattgaccgcgtatccggccagacccagttcaacggcgtgaacgtgctggcgaaagacggttcaatgaaaatt : 300
H6_AY249991.1      : ggacgaaatcaaatcccgtctcgacgaaattgaccgcgtatccggtcagacccagttcaacggcgtgaacgtactggcaaaagacggttcgatgaaaatt : 300
H18_AY250001.1     : ggacgaaatcaaatctcgtctggacgaaattgaccgcgtatccggtcagacccagttcaacggcgtgaacgtgctgtccaaagatggttcaatgaaaatt : 300
H34_AY250016.1     : ggacgaaatcaaatcccgtctcgacgaaattgaccgcgtatccggtcagacccagttcaacggcgtgaacgtactggcaaaagacggttcgatgaaaatt : 300
H14_AY249998.1     : ggacgaaatcaagtctcgtctggacgaaattgaccgcgtatccggtcagacccagttcaacggcgtgaacgtgctggcgaaagacggttcgatgaaaatt : 300
H15_AY249999.1     : ggacgaaattaaatcccgtctggacgaaattgatcgcgtatccggtcagacccagttcaacggcgtgaacgtgctggcaaaagacggttccatgaaaatt : 300
H20_AY250003.1     : ggacgaaatcaaatcccgtctggacgaaattgaccgcgtatccggtcagacccagttcaacggcgtgaacgtgctggcaaaagacggttccatgaaaatt : 300
H31_AY250013.1     : ggacgaaatcaaatcccgtctggacgaaattgaccgcgtatctggccagacccagttcaacggcgtgaacgtactggcgaaagacggttcaatgaaaatt : 300
H28_AY250010.1     : ggacgaaatcaaatcccgtctggacgaaattgaccgcgtatctggccagacccagttcaacggcgtgaacgtactggcgaaagacggttcaatgaaaatt : 300
H46_AY250024.1     : ggacgaaatcaaatcccgtctggacgaaattgaccgcgtatccggtcagactcagttcaacggcgtgaacgtactggcaaaagacggttccatgaaaatt : 300
H44_AB269770.1     : ggacgaaatcaaatcccgtctggacgaaattgaccgcgtatccggtcagacccagttcaacggcgtgaacgtgctggcaaaagacggctccatgaaaatt : 300
H19_AY250002.1     : ggacgaaatcaaatcccgtcttgacgaaattgaccgcgtatctggtcagacccagttcaacggcgtgaacgtgctgtctaaagatggctcgatgaaaatt : 300
H55_AB269771.1     : ggacgaaattaaatcccgtctggacgaaattgaccgcgtatccggtcagacccagttcaacggcgtgaacgtgctggcaaaagacggctccatgaaaatt : 300
H26_AY250008.1     : ggacgaaatcaaatctcgtctggacgaaattgaccgcgtatctggtcagacccagttcaacggcgtgaacgtgctgtctaaagatggctcgatgaaaatt : 300
H30_AY250011.1     : ggacgaaatcaaatcccgtcttgatgaaattgaccgcgtatccggccaaacccagttcaacggcgtgaacgtactgtcaaaagatggctcgatgaaaatt : 300
H32_AY250014.1     : ggacgaaatcaaatctcgtcttgatgaaattgaccgcgtatctggtcagacccagttcaatggcgtgaatgtgttgtccaaagacggttcaatgaaaatt : 300
H37_AY250017.1     : ggacgaaattaaatcccgtctggacgaaattgaccgcgtatccggtcagacccagttcaacggcgtgaacgtactggcaaaagacggttccatgaaaatt : 300
H41_AY250020.1     : ggacgaaatcaaatcccgtctggacgaaattgaccgcgtatccggccagacccagttcaacggcgtgaacgtgctggcgaaagacggttcaatgaaaatt : 300
H9_AY249994.1      : ggacgaaatcaaatcccgtctggacgaaattgaccgcgtatccggccagacccagttcaacggcgtgaacgtgctgtccaaagatggctcgatgaaaatt : 300
H36_EF392693.1     : aaatgaaattacccaacgtctggaggaaatcaaccgtgtttctggtcagactcagttcaacggtgtgaaagtactggcatctgataactctatgaccatt : 300
                     gga GAaAT aaa    CGtcT ga GAaATtgAcCG GT TC gg CAgAC cAgTT AAcGGcGTgAa GT cTggc  aa A      c aTgaa ATt

                             *       320         *       340         *       360         *       380         *       400
H21_AIHL01000060.1: caggttggtgctaatgatggtgaaaccatcactatcaatctggcaaaaattgatgcgaaaactctcggcctggacggtttaatatcgatggcgcgcaga : 400
H8_AJ865465.1      : caggttggtgctaatgatggtgaaaccatcactatcaatctggcaaaaattgatgcgaaaactctcggcctggacggtttaatatcgatggcgcgcaga : 400
H40_AJ884568.1     : caggttggtgctaatgatggtgaagccatcactatcaatctggcaaaaattgatgcgaaaactctcggcctggacggtttaatatcgatggcgcgcaga : 400
H11_AY337465.1     : caggttggtgctaatgatggtgaaaccatcactatcaatctggcaaaaattgatgcgaaaactctcggcctggacggtttaatatcgatggcgcgcaga : 400
H27_AM231154.2     : caggttggtgctaatgatggggaaaccatcactatcaatctggcaaaaattgatgcgaaaactctcggcctggacggctttaatatcgatggcgcgcaga : 400
H2_AIHA01000023.1 : caggttggtgcaaacgacggcgaaacaattactattaaactgcaggaaattaattcgacacactgggattatctggtttggtattaaagatcctacta : 400
H16_AY337475.1     : caagtaggtgcgaacgatggtgaaaccattgagattgcccttgataaaattgatgctaaaaccttggggcttgataacttagcgtagcaccaggaaaag : 400
H3_AB128916.1      : caggttggcgcaaatgacggtcagactatagaaattggattagataaaattgatgcagacactctcggtctgaaagactttagcgtagcatctgcaaaag : 400
H54_AB128918.1     : caggttggtgccaatgatggtgagacaatagatattaacctgaaagaaataaattcacaaacacttggtttagataagcttaatgtccaaaaagcataca : 400
H53_AB128917.1     : caagttggcgctaatgatggagagaagattgatatcgatttgaagaaaattgacacaggtactctcggcttagcaaacttctctgttgattcaaaatttg : 400
H47_EF392694.1     : caggttggcgctaatgataaccaatcaattgacattaatctgaaaaaaattgactcaactgttctcaaattacgtgacttagatgtcgtatctgaaactc : 400
H35_EF392692.1     : caggttggtgctaacgacggcgaaacaattacgattgatttaaaagaaattaatagcaaaactttgggattggataaattagatgtgcgtaacacattta : 400
H43_AIGA01000038.1: caggtaggcgcgaacgatggtcagactatctctatcgatctgaaaaaaatcgactcttcaaccctgggcctgaccggtttgatgttcgacgaaagcga : 400
H17_CP002291.1     : caggttggcgcgaatgatggtcagaccatcaccattgacctgaagaaaattgactctgcgacgctgggtctgacgggtttgatgtgcagaaaaagagta : 400
```

```
H5_AY249990.1      : caggttggcgcgaatgatggcagactattagcattgatttgcagaagattgactcttctacattaggactgaacggttctccgtttcgggtcagtcac :  400
H56_AY250029.1     : caggttggcgcgaatgatggccagactattagcattgatttacagaaaattgactcttctacattagggttgaatggtttctccgtttctgctcaatcac :  400
H42_AY250021.1     : caggttggcgcgaatgatgggcagaccatctctatcgatttgcagaaaatagactcttctgcccttggtttaagtggttttagtgttgccggtggggcgc :  400
H29_AY250012.1     : caggttggtgcgaacgatggtcagactatatccatcgacctgcaaaaaatcgactcttctactcttggtttgaacggtttctccgtttctaaaaatgctc :  400
H38_AY250018.1     : caggtcggtgcaaatgatggtcagacaatcagcattgatttgcagaagattgattcttctactttagggttaaatggttttctgtttccaaaaatgcag :  400
H33_AY250015.1     : caggttggcgcgaatgatgggcagaccatcaacatcgacctgcagaaaatcgactcttctactctgggcctggcggcttctccgtatctaacaatgcac :  400
H39_AY250019.1     : caggttggcgcgaacgatggccagactatctctatcgacctgcagaaaatagactcttctactctgggtctgagcggcttctctgtttctcagaactccc :  400
H52_AY250028.1     : caggttggcgcgaatgacgggcagaccatctctatcgatttgcagaaaattgattcttcaacgctgggattgaaaggtttctcggtatcagggaacgcat :  400
H4_AJ536600.1      : cagattggtgccaatgataaccagacgatcagcattggcttgcaacaaatcgacagtaccactttgaatctgaaaggatttaccgtgtccggcatggcg- :  399
H25_AGSG01000116.1: cagattggtgccaatgataaccagacgatcagcattggcttgcaacaaatcgacagtaccactttgaatctgaaaggatttaccgtgtccggcatggcg- :  399
H10_AY249995.1     : caggttgggcaaatgatggacagactatcactattgatctgaaaaagatcgattcatctacactaaacctctccagttttgatgctacaaacttgggca :  400
H24_AY250006.1     : caggtaggtgcaaatgacggtcagactatcaatattgacctgcagcaaatcgattctcatacactgggtctggatggtttcagcgttaaaaataatgatg :  400
H48_AY250025.1     : caggttggcgcaaatgataaccagactatcactatcgatctgaagcagattgatgctaaaactcttggccttgatggttttagcgttaaaaataacgata :  400
H1_AB028471.1      : caggttggtgcgaacgacggccagactatcactattgatctgaagaaaattgactctgatacgctggggctgaatggttttaacgtgaatggttccggta :  400
H12_AY337471.1     : caggttggtgcgaatgacggccagactatcacgattgatctgaagaaaattgactcagatacgctggggctgaatggttttaacgtgaatggttccggta :  400
H51_AY250027.1     : caggttggcgcaaatgatggtcagactatctctatcgacctgaagaaaattgactctgatactctgggtttgaatggtttcaacgttaatggttctggta :  400
H45_AY250023.1     : caggttggtgcgaatgacggccagactatcacgattgatctgaagaaaattgactcagatacgctggggctgaatggtttcaacgttaatggcaaaggca :  400
H49_AY250026.1     : caggttggtgcgaatgacggccagactatcactattgatctgaagaaaattgactctgatacgctggggctgaatggttttaacgttaacggcaaaggta :  400
H23_AB028476.1     : caggtaggtgctaacgacggccagactatcactattgacctgaaaaaaatcgactctgatactctgggcctgaatggttttaacgtgaatggttctggga :  400
H7_AY337468.1      : caggttggtgcgaatgacggcgaaaccatcacgatcgacctgaaaaaaatcgattctgatactctgggtctgaatggctttaacgtaaatggtaaaggta :  400
H6_AY249991.1      : caggttggtgcgaatgacggccagactatcactattgatctgaagaaaattgactctgatacgctggggctgaatgggtttaatgtgaacggcaaagggg :  400
H18_AY250001.1     : caggtcggcgcaaatgatggtgaaaccatcacgattgatctgaagaaaattgactctgatacgctgaatctggctggttttaacgtgaatggcgaaggtg :  400
H34_AY250016.1     : caggttggtgcgaacgacggccagactatcactattgatctgaagaaaattgactctgatacgctggggctgagtgggtttaacgtaaatggtagcgcag :  400
H14_AY249998.1     : caggttggtgcgaatgacggccagactatcacgattgatctgaagaaaattgactcagatacgctggggctgagtgggtttaatgtgaatggtggcgggg :  400
H15_AY249999.1     : caggttggcgcgaacgatggccagaccatcactatcgacctgaagaagattgactcttctaccttgaacctgacaggttttaacgttaacggttctggtt :  400
H20_AY250003.1     : caggttggcgcgaatgatggccagaccatcactatcgacctgaagaagattgactcttctacgttgaaactgactggttttaacgtgaatggttctggtt :  400
H31_AY250013.1     : caggttggtgcgaatgacggccagactatcactattgatctgaagaaaattgactcagatacgctggggctgagtgggtttaatgtgaatggtggcgggg :  400
H28_AY250010.1     : caggttggtgcgaatgacggccagactatcacgattgatctgaagaaaattgactctgatacgctggggctgagtgggtttaatgtgaatggtagcgggg :  400
H46_AY250024.1     : caggttggcgcgaatgatggccagaccatcactatcgacctgaagaagattgactcttctacgttgaaactgactggttttaacgtgaatggttctggtt :  400
H44_AB269770.1     : caggttggcgcgaacgatggccagaccatcactatcgacctgaaaaaaattgactcgtctactctgaacctgactggctttaacgttaatggtgaaggtt :  400
H19_AY250002.1     : caggtcggcgcgaacgatggcgaaacgattactattgatctgaagaaaattgactctgatacgctgaatctggctggttttaacgttaacggtaaaggtt :  400
H55_AB269771.1     : caggttggcgcgaacgatggtgaaaccatcaccatcgacctgaaaaaaattgactcttctactttaaacctgactgggtttaacgttaacggtaaaggta :  400
H26_AY250008.1     : caggtcggcgcgaacgatggcgaaacgattactattgatctgaagaaaattgactctgatacgctaaatctggctggttttaacgtgaatggtgctggct :  400
H30_AY250011.1     : caggtcggcgcaaatgatggtgaaaccatcacgattgatctgaaaaagatcgactcttctacattgaagctgaccagcttcaatgttaacggtaaaggcg :  400
H32_AY250014.1     : caggtgggcgcaaatgatggtgaaaccatcacgattgacctgaaaaaaatcgactcttctacactgaagctgaccagcttcaacgtcaacggtaaaggcg :  400
H37_AY250017.1     : caggttggcgcgaacgatggccagaccatcactatcgacctgaagaagattgactcttctacgctgaaactgactggttttaacgtgaatggcaaagcag :  400
H41_AY250020.1     : caggttggtgcgaatgacggccagactatcactattgatctgaagaaaattgactctgatactctgggtttgagtggatttaatgtgaatggcaaagggg :  400
H9_AY249994.1      : caggtcggcgcgaacgatggcgaaacgattactattgatctgaagaaaattgactctgatacgctgaatctggctggttttaacgttaacggtaaaggtt :  400
H36_EF392693.1     : caggttggcgctaatgatggagaagctattactatagacctaaagaaattacagctgagactttgggtttaaccggatttaacgttaatggtaaggta :  400
                     CAggTtGG GC AA GA gg A ac AT a  AT ga cTg a aAaAT ga  c    ac  T     Tg   gg tT    gt

                          *      420        *       440       *       460       *       480       *       500
H21_AIHL01000060.1: aagcaaccggcagtgacctgatttctaaatttaa---agcgacaggtactgataattatcaa------------------------------------at :  461
H8_AJ865465.1      : aagcaacaggcagtgacctgatttctaaatttaa---agcgacaggtactgataattatgat------------------------------------gt :  461
H40_AJ884568.1     : aagcaaccggcagtgacctgatttctaaatttaa---agcgacaggtactgataactatgat------------------------------------gt :  461
H11_AY337465.1     : aagcaactggcagtgacctgatttctaaatttaa---agcgacaggtactgataactatgat------------------------------------gt :  461
```

```
H27_AM231154.2    : aagcaactggcagtgacctgatttctaaatttaa---agcgacaggtactgataattatcaa------------------------------------at :  461
H2_AIHA01000023.1 : aattaaaagccgcaacggctgaaacaacctatttggatcgacagttaagcttgctgacg----------------------------------------- :  460
H16_AY337475.1    : ttccaatgtcctctgcggttgcacttaagagcga---agccgctcctgacttaactaaggtaaat------------------------------gcaac :  467
H3_AB128916.1     : tgcctacatcaggagcggttgcattaaaaagtga---aatgtctccaacattaacctctgtaaat------------------------------gcaac :  467
H54_AB128918.1    : caccaagtggcgaaaatgtgaaagttgacactac---aacttatactacaacttatactgatggca---------------------------------- :  463
H53_AB128917.1    : attcaacaagcactaaaaccgttgctaatggcgg------------------------------------------------------------------ :  434
H47_EF392694.1    : aatataaagacggtgccaccacgcttaaaggagatatcaaggacagtacatccgttcaaggaaaatttg------------------------------- :  469
H35_EF392692.1    : gcacatctgatctagctgcaactgcaactactgaactagctccggctaaaacagatgttaaa-------------------------------------- :  462
H43_AIGA01000038.1: atatttctacgacagcagtaacgggggcggcaac---gaccacttatgctgatagcgccgtt---------------g---------------------- :  460
H17_CP002291.1    : ctataagcaaagctgccgtagaaattactacgac---taccactccatatactgaaggcaataata---------------------------------- :  463
H5_AY249990.1     : ttaacgttagtgattccattactcaaattaccgg---tgccgccg---ggacaaaacctgtt-------------------------------------- :  456
H56_AY250029.1    : ttaacgttggtgattcaattactcaaattacagg---agccgctg---ggacaaaacctgtt-------------------------------------- :  456
H42_AY250021.1    : taaaattaagcgatacagtgacgcaggtcggcga---tggttca------gccgcgccagtt-------------------------------------- :  453
H29_AY250012.1    : tcgaaactagcgaagcgatcactcagttgccgaa---cggtgcg------aatgcaccaatc-------------------------------------- :  453
H38_AY250018.1    : tatctgttggtgatgctattactcaattgcctgg---cgagacggcagccgatgcaccagta-------------------------------------- :  459
H33_AY250015.1    : tgaaactgagcgattctatcactcaggttggtgc------------gagtggttcactggca-------------------------------------- :  450
H39_AY250019.1    : tgaaactgagcgattctatcactacgatcggcaa---tactact------gctgcatcgaag-------------------------------------- :  453
H52_AY250028.1    : taaaagttagcgatgcgataactacagttcctgg---tgctaatgctggcgatgccccggtt-------------------------------------- :  459
H4_AJ536600.1     : --gatttcagcgcggcgaaactgacggctgctga---tggtacagcaa---------------------------------------------------- :  442
H25_AGSG01000116.1: --gatttcagcgcggcgaaactgacggctgctga---tggtacagcaa---------------------------------------------------- :  442
H10_AY249995.1    : ccagtgttaaagatggggccaccatcaataagca---agtggcagtaggtgctggcgacttt------------------------aaag---------- :  463
H24_AY250006.1    : cagtgaaaaccagtgctgccgtgaatactcttgg---gggggggcaggttctgttgctgtcgact----------------------------------- :  463
H48_AY250025.1    : cagttaccactagtgctccagtaactgcttttgg---tgctaccaccacaaacaatattaaa-------------------------------------- :  459
H1_AB028471.1     : cgatagccaataaagcggcgaccattagcgacct---gacagcagcgaaaatggatgctgca-------------------------------------- :  459
H12_AY337471.1    : cgatagccaataaagcggcgaccattagcgacct---gacagcagcgaaaatggatgctgca-------------------------------------- :  459
H51_AY250027.1    : ccattgcaaacaaagcggccacaatcagtgactt---gactgctcagaaagccgttgacaac-------------------------------------- :  459
H45_AY250023.1    : ctattgcgaacaaagctgctacagtcagcgatct---gaccgctgctggtgcaacgggaaca-------------------------------------- :  459
H49_AY250026.1    : ctattgcgaacaaagcggcaaccattagtgatct---ggcggcgacgggggcgaatgttact-------------------------------------- :  459
H23_AB028476.1    : cgattaccaacaaagcagcaactgtcagtgatgt---tactcgcgcaggcggtacattggtg-------------------------------------- :  459
H7_AY337468.1     : ctattaccaacaaagctgcaacggtaagtgattt---aacttctgctggcgcgaagttaaac-------------------------------------- :  459
H6_AY249991.1     : aaacggctaatacggcagcaaccctgaaagatat---gtctggattcacagctgcggcggca---------------------------ccaggggggaac :  470
H18_AY250001.1    : aaacagccaatactgctgcaacacttaaagatat---ggttggtttaaaactcgataatacg---------------------------ggggtcactac :  470
H34_AY250016.1    : ataaggcaagtgtcgcggcgacagctgacggaat---ggttaaagacggatatatcaaaggg---------------------------ttaacttcatc :  470
H14_AY249998.1    : ctgttgctaacactgctgcatctaaagctgactt---ggtagctgctaatgcaactgtggta-------------------------------------- :  459
H15_AY249999.1    : ctgtggcgaatactgcagcaactaaagctgattt---aaccgctgctcaactctctgcaccg------------------------ggtgcagcagacgc :  473
H20_AY250003.1    : ctgtggcgaatactgcggcgactaaagcggattt---ggctgctgctgcaattggtacccct------------------------ggggcagcagattc :  473
H31_AY250013.1    : ctgttgctaatactgcagcgactaaagatgattt---ggtcgctgcatcagtttcagctgcg-------------------------------------- :  459
H28_AY250010.1    : ctgtggctaatactgcagcgactaaatctgattt---ggcagcagctcaactcttggctcca------------------------ggtactgctgatgc :  473
H46_AY250024.1    : ctgtggcgaatactgcggcgactaaagacgaact---ggctgctgctgctgcggcggcgggt------------------acaactcctgctgtcggtac :  479
H44_AB269770.1    : cagtagctaataaggcagcaactaaagctgattt---gacagctgctcagctcactacaacagctgctggcggtactatcgctgctcctgctgcagatgc :  497
H19_AY250002.1    : ctgtagcgaataccgctgcgactacagataatct---gacattggctggttttacagcgggt---------------------------actaaagctgc :  470
H55_AB269771.1    : gtgtgtctaacactgcagcgactactgatacact---taagctggctggtttcaccgctggt------------------------gcaactcctgctgc :  473
H26_AY250008.1    : ctgttgataatgccaaggcgactggcaaagatct---tactgatgctggttttacggcaagc---------------------------gcagctgatgc :  470
H30_AY250011.1    : ctgttgataatgctaaagccactgaagcagatct---gaccgctgcgggcttctcccaaggt---------------------------gcagt---cgt :  467
H32_AY250014.1    : ctgttgataatgcaaaagccactgaagcagatct---gaccgctgcgggcttctcccaaagt---------------------------gcagt---tgt :  467
H37_AY250017.1    : cggttgataatgctaaagcgacggatgcaaatct---gactaccgccggttttacacaaggc---------------------------gttgtggattc :  470
```

```
H41_AY250020.1    : ctgtggctaacgcaaaagcgaccgaagcagattt---aacggggctggtttctctcaagga-----------------------------gcggtggatac :  470
H9_AY249994.1     : ctgtagcgaatacagctgcgacaagcgacgattt---aaaactggctggtttcactaagggc----------------------------accacagatac :  470
H36_EF392693.1    : gcgtaaataacactgttgcaacagctaaggattt---aacagataaaggttttattagtact--------------------------------------- :  459
                                  g

                        *       520       *       540       *       560       *       580       *       600
H21_AIHL01000060.1: taacggtactgataactatactg-------------------------------ttaatgtagatagtggagt------agtacaggataaagatggc--- :  522
H8_AJ865465.1     : tggcggta---aaacttataccg-------------------------------tgaatgtggagagcggcgc------ggttaagaatgatgctaat--- :  519
H40_AJ884568.1    : tggcggtg---atgcttataccg-------------------------------ttaacgtagatagcggtgc------agtcaaggataaagataat--- :  519
H11_AY337465.1    : tggcggtg---atgcttatactg-------------------------------ttaacgtagatagcggagc------tgttaaagatactacaggg--- :  519
H27_AM231154.2    : taacggtactgataactatactg-------------------------------ttaatgtagatagtggagc------agttcaaaatgaggatggt--- :  522
H2_AIHA01000023.1 : ------------------------------------------------------ctaatacacttgatgcaga------tattacagctacagttaaa--- :  498
H16_AY337475.1    : tgatggtagtgtgggaggtgctaaa------gcattcggtagcaatt------ataaaaatgctgatgttga------aacttattttggtaccggt--- :  546
H3_AB128916.1     : tactggtaaaaatggtactaactac------gcatttggagctacat------tccgcactgatgatgttaa------tacttattttgg---cgga--- :  543
H54_AB128918.1    : ------------------------------------------------------ctgctatcaaaaataaagc------tgcattagctactgatgtt--- :  501
H53_AB128917.1    : tgatatcg----------ttctgtc-----------------------------ctctaaaactattaaagcaga------aatccagattgatagtcatag- :  491
H47_EF392694.1    : ------------------------------------------------------atactacatctgaggcagc------tatttcgtttaaggatggga-- :  508
H35_EF392692.1    : -aatc---tgacgtttactgatggc------gcaacag----------------ctcctacatttaagttgta------cgttgataatacagatcct--- :  528
H43_AIGA01000038.1: ------------------------------------------------------caattgatatcggaacgga------tattagcggtattgctgct--- :  498
H17_CP002291.1    : ------------------------------------------------------ccatcgcgttaaaatcatc------tgatacggctaatatgaaa--- :  501
H5_AY249990.1     : -------------------------------ggtgttgatttcactg-------ctgttgcgaaagatctgac---tactgcgacaggtaaaacagtc--- :  513
H56_AY250029.1    : -------------------------------ggtgttgatttcactg-------ctgttgcgaaagatctgac---tactgcgacaggtaaaactgtc--- :  513
H42_AY250021.1    : -------------------------------aaagtggatctggatg-------cagcagcaacagatattgg---tactgctttggggcaaaaggtt--- :  510
H29_AY250012.1    : -------------------------------gctgtgaagatggatgcgt----ctgttctgaccgatcttaa------------------cattact--- :  498
H38_AY250018.1    : -------------------------------accatcaagtttgatgatt----cagtaaaaactgatttaaa------------------actgacc--- :  504
H33_AY250015.1    : -------------------------------gatgtgaaactgagct-------ctgttgcctcggctctggg---------------------tgta--- :  489
H39_AY250019.1    : -------------------------------aacgtggacctgagcg-------cagtagcaactaaactgggc-g--------------------tg--- :  492
H52_AY250028.1    : -------------------------------acggttaaatttggtgcgaacgataccgctgctgccgcaat---ggctaaaacattgggaataagt--- :  522
H4_AJ536600.1     : ------------------------------------------------------ttgctgctgcggatgtcaa---ggatgctggggggtaaacaagtc--- :  483
H25_AGSG01000116.1: ------------------------------------------------------ttgctgctgcggatgtcaa---ggatgctggggggtaaacaagtc--- :  483
H10_AY249995.1    : ------------------------------------------------------ataaagcttcaggatcgtt---------------------------a :  483
H24_AY250006.1    : ------------------------------------------------------tcgcaacaaccagtttgac------tgctatcactggtctcggtagc :  504
H48_AY250025.1    : -------------------------------cttactggaatta----------ccctttctacggaagcagc------cactgatactggcggaact--- :  510
H1_AB028471.1     : -------------actaatactata------actacaacaaataatgcgc---tgactgcatcaaaggccct------tgatcaactgaaagatggt--- :  528
H12_AY337471.1    : -------------actaatactata------actacaacaaataatgcgc---tgactgcatcaaaggcgct------tgatcaactgaaagatggt--- :  528
H51_AY250027.1    : ----ggtaatggtacttataaagtt------acaactagcaacgctgcac---ttactgcatctcaggcatt------aagtaagctgagtgatggc--- :  537
H45_AY250023.1    : ----------ggtccttatgctgtg------accacaaacaatacagcac---tcagcgctagcgatgcact------gtctcgcctgaaaaccgga--- :  531
H49_AY250026.1    : ----aactcaagcaatattgttgtc------acgacaaagttcaatgcct---tggatgcagcgactgcatt------tagcaaactcaaagatggt--- :  537
H23_AB028476.1    : -------aatggtgcctatgatata------aaaaccactaacacagcgc---tgactacaactgatgcctt------cgcgaaattgaatgatggt--- :  534
H7_AY337468.1     : -accacgacaggtctttatgatctg------aaaaccgaaaataccttgt---taactaccgatgctgcatt------cgataaattagggaatggc--- :  540
H6_AY249991.1     : tgttggtgtaactcaatatactgac-------------aaatcggctgtag---caagtagcgtagatattctaaatgctgttgctggcgcagatgga--- :  552
H18_AY250001.1    : agctggagttaatagatat-------------attgctgacaaagccgtcg---caagtagcacggatatttgaatgcggtagctggtgttgatggc--- :  552
H34_AY250016.1    : tgacggcagcactgcatatactaaa------actacagcaaatactgcag---caaaaggatctgatattct------tgcggcgcttaagactggc--- :  552
H14_AY249998.1    : -------ggcaacaaatatactgtg------agtgcgggttacgatgctg---ctaaagcgtctgatttgct------ggctggagttagtgatggt--- :  534
H15_AY249999.1    : aaatggtacagttacttatactgtc------agtgctggttataaagaat---ccactgctgcagatgttat------tgctagcatcaaagacggc--- :  555
H20_AY250003.1    : tacaggtgccattgcttacacagta------agtgctgggctgactaaaa---ctacagccgcagatgtact------gtctagcctcgctgatggt--- :  555
```

```
H31_AY250013.1     : ----gtaggtaatgaatacactgtc------tctgctggcctgtcgaaat---caactgctgctgatgttat------tgctagtctcacagatggt--- :  537
H28_AY250010.1     : taatggtacagttacctatactgtt------ggcgcaggcctgaaaacat---ctacagctgcagatgtaat------tgcgagtttggctaataac--- :  555
H46_AY250024.1     : tgacggcgtgaccaaatataccgta------gacgcagggcttaacaaag---ccacagcagcaaacgtgtt------tgcaaaccttgcagatggt--- :  561
H44_AB269770.1     : taatggcgtaactaagtatacagta------agcgcgggattgaacgaat---ctaccgtagctgacgtgtt------tgctggcttaggtgatact--- :  579
H19_AY250002.1     : tgatggcaccgtaacttatagcaaa------aatgtccagtttgccgccg---cgactgcaagcaatgtact------ggctgctgctaaagatggc--- :  552
H55_AB269771.1     : tgacggtactgtgacttacagcaaa------gatgtggacaatgcaaaag---cggcagcaagtaatgtgct------tgctgccgctaaaaacggc--- :  555
H26_AY250008.1     : taatggcaaaatcacttataccaaagacaccgttactaaattcgacaaag---cgacagcggctgatgtatt------gggcaaagcggctgctggc--- :  558
H30_AY250011.1     : cagtggcaacagcacctggactaaatctactgttactacctttaatgcag---caacagctaccgacgtgct------ggcaagcgttagcggcggc--- :  555
H32_AY250014.1     : cagtggcaatagcacctggactaaatctactgttactacctttaatgcag---caacagctaccgatgtgct------ggctagcgttagtggcggc--- :  555
H37_AY250017.1     : aaatggtaatagtacttggactaaatcaactacgactaatttcgatgcgg---caactgcagtaaacgtact------agcagcagttaaagatggc--- :  558
H41_AY250020.1     : aaacggaaatagtacttggacaaaatcaaccaccaccaattactcagctg---caacaactgctgacttgtt------atcgaccattaaggatggc--- :  558
H9_AY249994.1      : caatggcgtgaccgcgtatacaaac------acaattagtaatgacaaag---ccaaagcttccgatctgtt------agctaatatcaccgatgga--- :  552
H36_EF392693.1     : -gataatggtaagacctatactggc------tctgcaggtttagctaatg---caaaagctggtgatgtatt------tggtaagatggttgatact--- :  540

                          *       620       *       640       *       660       *       680       *       700
H21_AIHL01000060.1: aaaca------------agttta---tgtgagtgctgcg---gatggttcac---------ttacgaccagca---------gtgatactcaattc---- :  582
H8_AJ865465.1      : aaaga------------tgtttt---tgtaagcgcagct---gatggatcgc---------tgacgaccagta---------gtgatactaaagtatccg :  583
H40_AJ884568.1     : aaaga------------tgtttt---tgttagcgctgcc---gatggttcgc---------tgaccaccagta---------gtgatacgaaagttgatg :  583
H11_AY337465.1     : aatga------------tatttt---tgttagtgcagca---gatggttcac---------tgacaactaaat---------ctgacacaaacatagctg :  583
H27_AM231154.2     : gacgc------------aatttt---tgttagcgctacc---gatggttctc---------tgactactaaga---------gtgatacaaaagtcggtg :  586
H2_AIHA01000023.1 : ggcac------------tacgac---tccgggccaacgtgacggtaatattat-----gtc--------------------------------------- :  539
H16_AY337475.1     : aatgt------------acaaga---tacaaaggatacaactgatgcgaccg---------gtactgcaggaacaa------aagtttatcaagtacagg :  616
H3_AB128916.1      : aaagc------------tgttaa---tactggtgataaaacaaatgtattag---------gca---cagaggcgg------aagtattccagattcagg :  610
H54_AB128918.1     : aataa------------tgcttc---ttcgattggggttagtgacgcaattc------------------------------caggtgatatcaaattta :  556
H53_AB128917.1     : -----------------tgctga---tccaaaagcggca---gacggtttgt---------atgcattgaaggatg----gt------------------ :  537
H47_EF392694.1     : aatac-ta---------cgctgt----------------------------------------------------------------------------- :  521
H35_EF392692.1     : gacaa--a----ttattcgtttc---ttcaaatggtacc---aactattacg------------------------------------ctgctagttacg :  580
H43_AIGA01000038.1: gatgc------------------------------------------------------------------------tgcgttaggaacgatcaatttcg :  526
H17_CP002291.1     : gatgg---------------------cacaacgaatgcaa------------------------------------------aagtttcgttctcattcg :  538
H5_AY249990.1      : gatgt------------ttctag----------------------------------------------------------------------------- :  524
H56_AY250029.1     : gatgt------------ttccag----------------------------------------------------------------------------- :  524
H42_AY250021.1     : aatgc------------aagttc----------------------------------------------------------------------------- :  521
H29_AY250012.1     : gatgc------------ttccgc----------------------------------------------------------------------------- :  509
H38_AY250018.1     : gatgc------------ttcagg----------------------------------------------------------------------------- :  515
H33_AY250015.1     : gacgc------------aagcac----------------------------------------------------------------------------- :  500
H39_AY250019.1     : aatgc------------aagcac----------------------------------------------------------------------------- :  503
H52_AY250028.1     : gatac------------atcagg----------------------------------------------------------------------------- :  533
H4_AJ536600.1      : aatttac----------tgtctt----------------------------------------------------------------------------- :  496
H25_AGSG01000116.1: aatttac----------tgtctt----------------------------------------------------------------------------- :  496
H10_AY249995.1     : ggtac------------------------------------------------------------------------------------cctaaaattag :  499
H24_AY250006.1     : ggtgc------------tatcag---cgaaattgctaaa------------------------------------------------------------- :  528
H48_AY250025.1     : aaccc------------------------------------------------------------------------agcttcaattgagggtgtttata :  538
H1_AB028471.1      : gacac------------tgttac---tatcaaagcagatgcagctcaaactg------------------------------ccacggtctatacataca :  583
H12_AY337471.1     : gacac------------tgttac---tatcaaagcagatgctgctcaaactg------------------------------ccacggtttatacataca :  583
H51_AY250027.1     : gatac------------tgtaga---tattgcaacctatgctggtggtacaag------------------------ttcaacagttagttataaatacg :  598
```

```
H45_AY250023.1    : gatac------------agttac---tactactggctcgag---------------------------------------tgctgcgatctatacttatg :  577
H49_AY250026.1    : gattc------------tgttgc---cgttgctgctcag------------------------------------------------aaatatacttata :  574
H23_AB028476.1    : gatgt------------tgttac---tatcaataatggtaa---------------------------------------ggatactgcctataaatata :  580
H7_AY337468.1     : gataa------------agtcac---agttggcggcgta------------------------------------------------gattatacttaca :  577
H6_AY249991.1     : aataa------------agttac---aactagcgccgat---gttggttttg---------gtacaccagccgctg------ctgtaacctatacctaca :  619
H18_AY250001.1    : agtaa------------agtttc---cacggaggcagat---gttggttttg---------gtgcagctgcccctggtacgccagtggaatatacttatc :  625
H34_AY250016.1    : gataa------------aattac---cgcaacaggtgca---aatagccttg---------ctgataatgcgacat------cgacaacttatacttata :  619
H14_AY249998.1    : gatac------------tgttca---ggcaaccattaat---aacggcttcg---------gaacggcggctagtg------caacgaattacaagtatg :  601
H15_AY249999.1    : agtgctccgacttctgcaattac---tgcaaccattaat---aatggcttcggtgattccagtgcgctgactt---------ccaatgactatacttatg :  640
H20_AY250003.1    : acgac------------tattac---agccacaggcgtgaaaaatggctttg---------ctgcaggagccactt------ccaatgcctataaactta :  625
H31_AY250013.1    : gcgac------------agtaac---tgcggctggtgtaagcaatggttttg---------ctgcaggggcaactg------gagatgcttataaattca :  607
H28_AY250010.1    : gcaaa------------agttaa---tgccacaattgca---aatggttttg---------gatcgccaacag---------ctacagattatacataca :  619
H46_AY250024.1    : gctgt------------tgttga---tgctagcatttcc---aacggttttg---------gtgcagcagcag---------ccacagactacacctaca :  625
H44_AB269770.1    : gccgt------------agttaa---tgctaatattacc---agtggtttcg---------acgctgttactg---------gtaataactatacgtatc :  643
H19_AY250002.1    : gacga------------aattac---gttcgctggtaat---aacggcacag---------gtatagctgcaactg------gggggacttatacttatc :  619
H55_AB269771.1    : gatac------------tatcag---cttcgccgggaat---aacggtacag---------gaatcactgctacgg------cgggaacttatacttata :  622
H26_AY250008.1    : gatag------------cattac---ctatgcgggcactgatactggcttag---------gagtcgctgctgatg------cctcgacttacacctaca :  628
H30_AY250011.1    : agcac------------tattagcggttataccggtacaaacaatggattag---------gcgtagcggctt---------ctactgcatatacctaca :  625
H32_AY250014.1    : agcac------------tattagcggttatgctggcacaaacaatgggttag---------gcgtagcggctt---------ctactgcatatacctaca :  625
H37_AY250017.1    : agcac------------aatcaa---ttacaccggtactggtaatggtttag---------ggattgctgcaa---------caagtgcttatacatatc :  625
H41_AY250020.1    : tctac------------tgttac---atatgcagggacagacaccggattag---------gggtcgcagcag---------caggaaattatacttatg :  625
H9_AY249994.1     : tcagt------------gatcac---tgggggaggggca---aacgcttttg---------gcgtggctgcaa---------agaatggttacacctatg :  616
H36_EF392693.1    : ggcac------------agttac---gacaactattgat---aatggtttcg---------gtactgcacaaa---------gtaatacgtacaaatatg :  604

                         *       720         *       740         *       760         *       780         *       800
H21_AIHL01000060.1: -----aagattgat-------------------------------------------------------------------------------------- :  591
H8_AJ865465.1     : gtgaaagtattgat-------------------------------------------------------------------------------------- :  597
H40_AJ884568.1    : gtaaaagtattgat-------------------------------------------------------------------------------------- :  597
H11_AY337465.1    : gtacagggattgat-------------------------------------------------------------------------------------- :  597
H27_AM231154.2    : gtacaggtattgat-------------------------------------------------------------------------------------- :  600
H2_AIHA01000023.1 : -tgatgctaacggtaagttgtacgttaaa----------------------------------------------------------------------- :  567
H16_AY337475.1    : tggaagggcagacttattttg------------------------------------------------------------------------------- :  637
H3_AB128916.1     : ttgaaggacaaacctactttg------------------------------------------------------------------------------- :  631
H54_AB128918.1    : acgaaacatctggaaaatattacgttgca----------------------------------------------------------------------- :  585
H53_AB128917.1    : ---------------------------------------------------------------------------------------------------- :    -
H47_EF392694.1    : ---------------------------------------------------------------------------------------------------- :    -
H35_EF392692.1    : ataaagacacaaactctattaa---------------------------gtttaactccacaggtactggtactacaggtaca----------------- :  636
H43_AIGA01000038.1: ataatacaacaggcaagtacta---------------------------cgcacagattaccagtgcggccaatc------------------------- :  574
H17_CP002291.1    : ataaagcaaccaactcctactt---------------------------tgcaatcgttgctgatggttcagcag------------------------- :  586
H5_AY249990.1     : -------------------------------------------cctgacgttacacaacactctggatgcgaaaggggctgct----------------- :  564
H56_AY250029.1    : -------------------------------------------cctgacgttacacaacaccctggatgcgaaaggggctgcc----------------- :  564
H42_AY250021.1    : -------------------------------------------tttaacgttgcacaatatcttagacaaagatggtgcggca----------------- :  561
H29_AY250012.1    : -------------------------------------------tgtttcgctgcacaacgt---aactaaaggtggtgtcgca----------------- :  546
H38_AY250018.1    : -------------------------------------------gttaagtctgcataacctcaaagatgaaaatggtaattta----------------- :  555
H33_AY250015.1    : -------------------------------------------tctgactctgcacaacgtacagaccccagctggcgcagca----------------- :  540
```

```
H39_AY250019.1    : -------------------------------------------cctgagcctgcacgaagttcaggactctgctggtgacggt----------------- :  543
H52_AY250028.1    : -------------------------------------------cttgtccctacataacgtacaaagcgcggatggtaaagcg----------------- :  573
H4_AJ536600.1     : ---------------------------------------------------------------------------------------------------- :   -
H25_AGSG01000116.1: ---------------------------------------------------------------------------------------------------- :   -
H10_AY249995.1    : ttgagaaagacggtaagtacta---------------------------tgtaaatgacactaaaa---------------------------------- :  538
H24_AY250006.1    : --gacgataatggtgattacta---------------------------cgcgcatgtcacagggactacgggta------------------------- :  574
H48_AY250025.1    : ctgataatggtaatgattacta------------------------------------------------------------------------------ :  560
H1_AB028471.1     : atgcatctgctggtaacttctc---------------------------attcagtaatgtatcgaataatacttcagcaaaag------------cagg :  644
H12_AY337471.1    : atgcatcagctggtaacttctc---------------------------attcagtaatgtatcgaataatacttcagaaaaag------------cagg :  644
H51_AY250027.1    : acgcagatgcaggtaacttcag---------------------------ttataacaatactgcaaacaaaacaagtgctgcgg------------ctgg :  659
H45_AY250023.1    : atgcggctaaagggaacttcac---------------------------cactcaagcaacagttgcagatggcgatg------------------ttgt :  632
H49_AY250026.1    : acgcatcgaccaatgattttac---------------------------gacagaaaatacagtagcgacaggcactgcaacga---------------c :  632
H23_AB028476.1    : atgctgctacaggtgggtttacgacggatgtctccat------------ctccggggatcctaccgctgctgacgctactgctaataaaactgcccgtga :  668
H7_AY337468.1     : acgctaaatctggtgatttactaccactaaatctactgctggtacgggtgtagacgccgcggcgcaggctgctgattcagctt------caaaacgtga :  671
H6_AY249991.1     : ataaagacactaattcatattc---------------------------cgccgcttctgatgatatttccagcg------------------------c :  668
H18_AY250001.1    : ataaagatactaacacatatac---------------------------ggcttctgcttcagttgatgc---------------------------gac :  671
H34_AY250016.1    : atgcaaccagcaataccttctc---------------------------ctatacggctgacggtgtaaaccaaa------------------------c :  668
H14_AY249998.1    : acagtgcaagtaagtcttactc---------------------------ttttgataccacaacggcttcagctg------------------------c :  650
H15_AY249999.1    : acccagcaaaaggcgacttcac---------------------------ttacgacgtagcttcaagcgccaata---------------atactgctgc :  698
H20_AY250003.1    : acaaagataataatacatttac---------------------------ttatgacacgactgctacgac---------------------------agc :  671
H31_AY250013.1    : atcaagcaaacaacacttttac---------------------------ttacaataccacctcaacagc---------------------------ggc :  653
H28_AY250010.1    : acagcgctacaggcgattttac---------------------------atatagtgcaactattgcagctggtacaaattctggtgatagtaacagtgc :  692
H46_AY250024.1    : ataaagctacaaatgatttcac---------------------------tttcaatgccagcattgctgctggtgctgcggccggtgatagtaacagcgc :  698
H44_AB269770.1    : ataaagatactaatgattttac---------------------------attcaatgctactattgcagctggtgacgcaacaactccaagtaatagcgc :  716
H19_AY250002.1    : ataaggactctaactcatacag---------------------------ctttagcgcaacggctgcatctaaag------------------------a :  668
H55_AB269771.1    : ataaagcctcagattcttatag---------------------------cttcagcgcaactgcagcttctaagg------------------------a :  671
H26_AY250008.1    : atgcagccaataagtcttacac---------------------------ttttgatgctactggtgttgccaagg------------------------c :  677
H30_AY250011.1    : acgcaaccagcaagtcttattc---------------------------atttgacgcaaccgcacttaccaatggcgatggtactg---gggccaccac :  695
H32_AY250014.1    : acgcaaccagcaagtcttattc---------------------------atttgacgcaaccgcacttactaatggtgatggtactg---cgggctcaac :  695
H37_AY250017.1    : acgatagcactaaatcctatac---------------------------ctttgattctacgggggctgcagtag------------------------c :  674
H41_AY250020.1    : atgcgaacagtaaatcttattc---------------------------cttcaatgccaatggtctgacgggcg------------------------c :  674
H9_AY249994.1     : atgcagcaagtaaatcttatag---------------------------ttttgctgcagatggtgccgattcag------------------------c :  665
H36_EF392693.1    : ataaagcaagtaatagttttc---------------------------tttcgatatcgatgacgctgctggtacaactgcac---------------c :  662

                         *      820       *      840       *      860       *      880       *      900
H21_AIHL01000060.1: ----------------------------------------gcaactaagcttgcagtggctgctaaagatt------------tagctcaaggtaataag :  639
H8_AJ865465.1     : ----------------------------------------gcaacagaactagcgaaacttgcaataaaat------------tagctgacaaaggctcc :  645
H40_AJ884568.1    : ----------------------------------------gcaacagagcttgcgaaacttgcaataaact------------tggctgaccagaaatcc :  645
H11_AY337465.1    : ----------------------------------------gctacagcactcgcagcagcggctaagaata------------aagcacagaatgataaa :  645
H27_AM231154.2    : ----------------------------------------gcgactgggcttgcaaaagccgcagtttctt------------tagctaaagatgcctca :  648
H2_AIHA01000023.1 : ----------------------------------------gttgccggttcagataaacccgctgaaaatg------------gttattatgaagttact :  615
H16_AY337475.1    : -----------------------------------------------------------------------------------ttggtcaagataataat :  654
H3_AB128916.1     : -----------------------------------------------------------------------------------tagcacaaaatggagat :  648
H54_AB128918.1    : ----------------------------------------ataacttcaacagaacataacgataaaaatg------------gtgattatgaaattact :  633
H53_AB128917.1    : ----------------------------------------accggatatgcagtaaaagataaagacggtg------------catatcacgctgctgtt :  585
```

```
H47_EF392694.1     : ---------------------------------------tgtatcaaagtctactaccacggataataatg------------gcatttatgccgcttca :  570
H35_EF392692.1     : ----------------------------------------gcgggaaccgattttattga---------------------------------------- :  656
H43_AIGA01000038.1: -----------------------------------------------------------cgggccttgatg------------gtgcttatgaaatccat :  603
H17_CP002291.1     : -----------------------------------------------------ataatactttaaaaaatg------------gtacatatattgcaact :  621
H5_AY249990.1      : ----------------------------------------acatcacagttcgtcgttcaatccggcaatg------------atttctactccgcgtcg :  612
H56_AY250029.1     : ----------------------------------------accgcacagttcgtcgttcaatccggtagtg------------atttctactccgcgtcc :  612
H42_AY250021.1     : ----------------------------------------actgagaactatgttgttagctatggtagtg------------ataattacgctgcatct :  609
H29_AY250012.1     : ----------------------------------------acgtctacttatgttgttcagtatggcgata------------agagctatgcagcatct :  594
H38_AY250018.1     : ----------------------------------------actaaccagtatgttgtacagaatggcggaa------------aatcttacgctgctaca :  603
H33_AY250015.1     : ----------------------------------------acagctaactatgttgtctcttctggttctg------------acaactactcagtatct :  588
H39_AY250019.1     : ----------------------------------------actggtaccttcgttgtttcttctggcagcg------------acaactatgctgtgtct :  591
H52_AY250028.1     : ----------------------------------------acaggaacctatgttgttcaatctggtaatg------------acttctattcggcttcc :  621
H4_AJ536600.1      : -----------------------------------------------------------------------------------acactgacaccgcgtct :  513
H25_AGSG01000116.1: -----------------------------------------------------------------------------------acactgacaccgcgtct :  513
H10_AY249995.1     : -----------------------------------------------------------------gtagta------------agtactacgatgccgaa :  561
H24_AY250006.1     : -----------------------------------------------------------atactgctgatg------------gttactatgctgtcgat :  603
H48_AY250025.1     : ---------------------------------------tgcgaaaatcaccggtggtgataacgatggga------------agtattacgcagtaaca :  609
H1_AB028471.1      : tgatgtagcagctagccttctcccgccggctgggcaa---actgctagtggtgtttacaaagcagcaagcg------------gtgaagtgaactttgat :  729
H12_AY337471.1     : tgatgtagcagctagccttctcccgccggctgggcaa---actgctagtggtgtttataaagcagcaagcg------------gtgaagtgaactttgat :  729
H51_AY250027.1     : aactctggcagatactcttctcccggcagctggccag---actaaaaccggtacttacaaggctgctactg------------gtgatgttaactttaat :  744
H45_AY250023.1     : taactttgcgaatactctgaaaccagcggctggcact---actgcatcaggtgtttatactcgtagtactg------------gtgatgtgaagtttgat :  717
H49_AY250026.1     : agatcttggcgctactctgaaggctgctgctgggcag---agtcaatcaggtacatatacctttgcaaatg------------gtaaagttaactttgat :  717
H23_AB028476.1     : tgcacttgcggcgtctttacatgctgagccgggtaaa---actgttaatggttcttggactacgaatgatg------------gtacggtaaaatttgat :  753
H7_AY337468.1      : tgcgttagctgccacccttcatgctgatgtgggtaaa---tctgttaatggttcttacaccacaaaagatg------------gtactgtttctttcgaa :  756
H6_AY249991.1      : taacctggctgctttcctcaatcctcaggccggagat---acgactaaagctacagttacaattggtggca------------aagatcaagatgtaaac :  753
H18_AY250001.1     : tcaactggcggcattcctgaatcctgaagcgggtggt---accactgctgcaacagtaagtattggcaacggtacaacagctcaagagcaaaaagtcatt :  768
H34_AY250016.1     : gaatgctgcagcaaatctcatacctgcagcagggaaa---acgacagctgcatcagttactattggtggga------------cagcacagaatgtaaat :  753
H14_AY249998.1     : cgatgttcagaaatatttgaccccgggcgttggtgat---accgctaagggcactattactatcgatggtt------------ctgcacaggatgttcag :  735
H15_AY249999.1     : ccaggttcagtccttcctgacgccgaaagcaggtgat---accgcaaatctgaaagtaaccgttggtacga------------catcggttgatgtcgtt :  783
H20_AY250003.1     : tgagctgcagtcttacctgactccgaaagcgggcgac---actgcaacattcagtgttgaaattggtggta------------ctacacaagacgtcgtg :  756
H31_AY250013.1     : agaactccaatcttacctcacgcctaaggcgggggat---accgcaactttctccgttgaaattggtggca------------ccaagcaggatgttgtt :  738
H28_AY250010.1     : tcagttacaatccttcctgacaccaaaagcgggcgat---actgctaacttaaacgttaaaattggttcta------------cgtcaattgacgttgta :  777
H46_AY250024.1     : agctctgcaatccttcctgactccaaaagcaggtgat---acagctaacctgagcgtcaaaatcggtacga------------catctgttaatgttgtt :  783
H44_AB269770.1     : caagttacaatctatcctgactcctaaagcaggtgat---accgctcatctgaacgttaagattggtgcaa------------catctgtagatgttgtt :  801
H19_AY250002.1     : ttctctgttgagcacactggcaccaaacgctggcgat---acatttaccgctaaagtgactattggttcta------------aatcgcaagaagttaac :  753
H55_AB269771.1     : ttctctccttagcatgttggcacctaacgctggtgac---agttttaccgcatctgtttctattggcggta------------aagcgcaggacgttaat :  756
H26_AY250008.1     : ggatgctggaacggcactgaaagggtacttaggcgca---tctaacaccggtaaaattaatatcggtggta------------ccgagcaagaagttaac :  762
H30_AY250011.1     : taaagttgctgatgtgctgaaagcctatgcagcaaacggtgataatacggctcagatctccatcggcggaa------------gcgctcaggacgttaaa :  783
H32_AY250014.1     : taaagttgctgatgttctgaaagcctatgcagcaaacggcgataacacggctcagatctccatcggtggta------------gcgctcaggaagttaaa :  783
H37_AY250017.1     : tggtgccgcgtccagcctgcaaggtacttttggtacagatacgaatactgcaaaaatcaccatcgatggtt------------ctgctcaagaagtaaac :  762
H41_AY250020.1     : aaataccgcaactgcactcaaaggttacttggggacaggtgctaacaccgctaaaatttctatcggtggta------------cagagcaggaagtgaat :  762
H9_AY249994.1      : gaagacgttaagcatcattaatccaaacaccggtgat---tcgtcgcaggcgacagtgactattggtggta------------aagagcagaaagttaat :  750
H36_EF392693.1     : acaagtagcaacttacttaaatccaactgctaatgat---aaaactaaagcttctgttgaaatcaatggct------------cttcccaagcggttatt :  747

                          *      920         *      940         *      960         *      980         *      1000
```

```
H21_AIHL01000060.1: attgtctacgaaggtatcgaatttacaaa----------taccggcactggcgctatacctgccacaggtaatggtaaattaaccgccaatgttgatggt :  729
H8_AJ865465.1     : attgaatacaagggcattacatttactaa----------caacactggcgcagagcttgatgctaatggtaaaggtgtttgaccgcaaatattgatggt :  735
H40_AJ884568.1    : attgactacaaaggtattacatttactaa----------caagagtggtactgcatttgatgctaatggtaaaggcgtttgactgcaaatattgatggt :  735
H11_AY337465.1    : ttcacgtttaatggagttgaattcacaac---------------aacaactgcagcggatggcaatgggaatggtgtatattctgcagaaattgatggt :  729
H27_AM231154.2    : attaaataccaaggtattactttcaccaa----------caaaggcactggtgcatttgatggcagtagtaacggcactctaatcgctaatattgatggc :  738
H2_AIHA01000023.1 : gtggaggatgat---------ccgacatc-------tcctgatgcaggtaagctgaagctggggctctagcggtacccagcctcaagctggtaattta :  699
H16_AY337475.1    : accaacacgaacggttttaca-ttattgaaacaaaactctacaggttatgaaaaagttcaggtgggtggtaaggatgttcagttagcaactttggtggt :  753
H3_AB128916.1     : actgccactaatgcttattcc-ttacttaagcaatctggttcaggttatgaaaaagtaactgttgatagtaaagctgttcagattgcgaattttggcggg :  747
H54_AB128918.1    : gttgataaagatggcagcg---ctgcatt----------ggttgcgggtcaatcgtctccaaaatctcttgaagatgtaggagccacaaaaatgtcacc :  720
H53_AB128917.1    : aaaaatgctgatggtaagg---tcacttg--------------------------------ggattcaagtgatacttctgtgacagcaactgatgtt :  648
H47_EF392694.1    : gttgactcggatggtaacg---ttacaat----------tgatgctagtaaga------------------------------------------------ :  610
H35_EF392692.1    : ------------------------------------------cgcgaccggtaaagatgtccatgtgggtggtggacgtgtaatggctgcaaatgatatcaaa :  717
H43_AIGA01000038.1: gttaatgacgcggatggttccttcactgt----------agcagcgagtgataaacaagcgggtgctgctccgggtactgctctgacaagcggtaaagtt :  693
H17_CP002291.1    : cttgactccagtggtgaag---tgac-------------tctgggtagttcaaccactgcgccaaatacagcaacagcagtaactaagaaccaagttgct :  705
H5_AY249990.1     : attaat--------------------------------------------------------catacagacggcaaagtcacgttgaataaagccgat :  654
H56_AY250029.1    : attgac--------------------------------------------------------catgcaagtggtgaagtgacgttgaataaagccgat :  654
H42_AY250021.1    : gttgc-----------------------------------------------------------agatgacggactgtaactcttaataaaacggat :  648
H29_AY250012.1    : gttga-----------------------------------------------------------tgcgggaggtacagtaaaactgaataaagccgac :  633
H38_AY250018.1    : gtcgc-----------------------------------------------------------tgccaatggtaatgttacgctgaacaaagcaaat :  642
H33_AY250015.1    : gttgaa--------------------------------------------------------gatagctccggtacagttacgctgaacaccactgat :  630
H39_AY250019.1    : gtagac--------------------------------------------------------gcggcctctggtgcagttaacctgaacaccactgac :  633
H52_AY250028.1    : gttaa-----------------------------------------------------------tgctggtggcgttgttacgcttaataccaccaat :  660
H4_AJ536600.1     : aacagtactaaatatgcgg--------------------------------------------------------------------------------- :  532
H25_AGSG01000116.1: aacagtactaaatatgcgg--------------------------------------------------------------------------------- :  532
H10_AY249995.1    : gtagatac--------------------------------------------------------tagtaagggtaaaattaacttcaactctacaaat :  603
H24_AY250006.1    : atcgacaagg--------------------------------------------------------ctaccggtgaggtcgctctgaaagatggtaac :  645
H48_AY250025.1    : gttgctaatgatggtacag---tgacaat----------ggcgactggagcaacggcaaatgcaactgtaactgatgcaaatactactaaagcta----- :  691
H1_AB028471.1     : gttgatgcgaatggtaaaa---ttac-------------aatcggaggacaggaagcctatttaactagtgatggtaacttaactacaacgatgctggt :  813
H12_AY337471.1    : gttgatgcgaatggtaaaa---tcac-------------aatcggaggacagaaagcatatttaactagtgatggtaacttaactacaacgatgctggt :  813
H51_AY250027.1    : gttgacgcaactggtaatc---tgac-------------aattggcggacagcaagcctacctgactactgatggtaaccttacaacaaacaactccggt :  828
H45_AY250023.1    : gtagatgctaatggcgatg---tgac-------------catcggtggtaaagccgcgtacctggacgccactggtaacctatctacaaacaaccccggc :  801
H49_AY250026.1    : gttgatgcaagcggtaata---tcac-------------tattggcggcgaaaaggctttcttgg---ttggtggagcgctgactactaacgatcccacc :  798
H23_AB028476.1    : accgatgccgatggtaaga---tttc-------------tattggtggtgttgctgcttatgtagatgcagcaggcaacctgaccactaacgcagcaggt :  837
H7_AY337468.1     : acggattcagcaggtaata---tcac-------------catcggtggaagccaggcatacgtagacgatgcaggcaacttgacgactaacaacgctggt :  840
H6_AY249991.1     : atcgataaatccggtaatttaactgctgc----------tgatgatggcgcagtactttatatggatgctaccggtaacttaactaaaaataatgctggt :  843
H18_AY250001.1    : attgctaaagatggttctttaactgctgc----------tgatgacggtgccgctctctatcttgatgatactggtaacttaagtaaaactaacgcaggc :  858
H34_AY250016.1    : attgatgattcgggcaata---ttacttc----------aagtgatggcgatcaactttatctggattcaacaggtaacctgactaaaaaccaggccggc :  840
H14_AY249998.1    : atcagcagtgatggtaaaa---ttacgtc----------aagcaatggagataaactttacattgatacaactggcgctt aacgaaaaacggctttagt :  822
H15_AY249999.1    : ctggccagtgatggtaaga---ttacagc----------aaaagatggttctgcattatatatcgacagtacaggtaacctgactcagaacagtgctggc :  870
H20_AY250003.1    : ctgtccagtgatggcaaac---tcactgc----------taaggatggctctaagctttacattgatacaactggtaatttaactcagaatggtggtaat :  843
H31_AY250013.1    : ctggctagtgatggcaaaa---tcacagc----------aaaagacgggtctaaactttatattgacaccacaggagaatttaacccaaaacggtggaggt :  825
H28_AY250010.1    : ttggctagcgacggtaaaa---ttaccgc----------gaaagatggttcagaactatttattgacgtagatggtaacctcactcaaaacaatgctggg :  864
H46_AY250024.1    : ctggcgagcgatggcaaaa---ttacagc----------gaaagatggctcagctctgtatatcgactcaacgggtaacctgactcagaacagcgcaggc :  870
H44_AB269770.1    : ctgtcaagcgatggtaaaa---tcactgc----------gaaagatggttccaagcttttcattggtattgatggcaacctgactcagaacagtgctggt :  888
H19_AY250002.1    : gttagcaaagatggtacga---ttacatc----------cagcgatggtaaggcgctgtatttagatgagaagggcaacctgacccaaacaggtagtggc :  840
H55_AB269771.1    : gttagcaaagatggcacga---tcactac----------taccgatggtaaatcgctgtatcttgatcagaaaggaaacctgactcagactggcagcggt :  843
```

```
H26_AY250008.1     : attgccaaagatggctcca---tcaccga----------taccaatggcgatgcgctgtatctcgatagtaccggcaacttaaccaaaaataccgcgaat :  849
H30_AY250011.1     : attgccagcgatggcaccc---tgactga----------cgtcaatggtgatgctttatatattggttctgacggcaacctgactaaaaaccaggccggc :  870
H32_AY250014.1     : attgccagcgatggtaccc---tgacgga----------tactaatggcgatgctttatacattggtgctgacggtaacctgacgaaaaaccaggccggc :  870
H37_AY250017.1     : atcgctaaagatgggaaaa---ttactga----------tactgatggtaaagctttatatatcgattccactggtaatttgactaagaacggctctgat :  849
H41_AY250020.1     : attgccaaagatggcacta---ttacaga----------tacgaatggtgatgcgctctatctggatattaccggcaacctgactaagaactatgcgggt :  849
H9_AY249994.1      : atttcccaggatggaaaaa---ttactgcg-------gcagatgataatgcgacgctgtatttagataaacaggaaacttgacaaaaacgaatgcaggt :  840
H36_EF392693.1     : attgatcataatggtaaaa---tgactgc-------tgcagatgataatgcagaacttttcattgataactctggtaatttaactaagaataacaaaact :  837
                      t                                                                       gg     t       aa

                          *     1020        *     1040        *     1060        *     1080        *     1100
H21_AIHL01000060.1: --------------------------aaggctgttgaattcactatttcg-------------------------------------------------- :  753
H8_AJ865465.1      : --------------------------caagatgttcaatttactatt----------------------------------------------------- :  756
H40_AJ884568.1     : --------------------------caagatgtaaaatttactatt----------------------------------------------------- :  756
H11_AY337465.1     : --------------------------aagtcagtgacatttactgtg----------------------------------------------------- :  750
H27_AM231154.2     : --------------------------aaagatgtaacctttactatt----------------------------------------------------- :  759
H2_AIHA01000023.1  : aag-----------------------gaagtcacaacggtgaaaggg----------------------------------------------------- :  723
H16_AY337475.1     : --------------------------cgtgtaactgcatttgttgaagata------------------------------------------------- :  778
H3_AB128916.1      : --------------------------cgagttacagcatttgttgacgatg------------------------------------------------- :  772
H54_AB128918.1     : gcatatcaagttgccaacaca-----------------------------------------------------------------------caatctaa :  749
H53_AB128917.1     : gac-----------------------caaaccacaaggttacagaattcc-------------------------------------------------- :  676
H47_EF392694.1     : ---------------------------------------------------------------------------------------------------- :    -
H35_EF392692.1     : ggccgtacagatgacaatgtagctgctaagcctcaacttttcttagacc--------------------------------------------------- :  766
H43_AIGA01000038.1: -----------------------cagactgcaacca---------------------------------------------------------------- :  706
H17_CP002291.1     : gga-----------------------acgcctgtta---------------------------------------------------------------- :  718
H5_AY249990.1      : gtcgaatacacagacaccgat-----aatggactaacga------------------------------------------------------------- :  688
H56_AY250029.1     : gtcgaatacaaagacaccgat-----aatggactaacga------------------------------------------------------------- :  688
H42_AY250021.1     : attacttattcaggc-----------ggtgatatta---------------------------------------------------------------- :  673
H29_AY250012.1     : gtaacatataacgacgcagca-----aatggtgtta---------------------------------------------------------------- :  664
H38_AY250018.1     : gtaacctacagcgatgtcgca-----aacggtattg---------------------------------------------------------------- :  673
H33_AY250015.1     : ataggttataccgataccgct-----aatggcgtta---------------------------------------------------------------- :  661
H39_AY250019.1     : gtcacctatgatgacgctact-----aatggtgtta---------------------------------------------------------------- :  664
H52_AY250028.1     : gttactttcactgatcctgcg-----aacggtgtta---------------------------------------------------------------- :  691
H4_AJ536600.1      : ---------------------------------------------------------------------------------------------------- :    -
H25_AGSG01000116.1: ---------------------------------------------------------------------------------------------------- :    -
H10_AY249995.1     : gaaagt---ggaactactcct-----actgcagcgacggaagtaa------------------------------------------------------- :  640
H24_AY250006.1     : gtagat-------acaccgaca----ggtacgccaacgacgacaagcacat------------------------------------------------- :  685
H48_AY250025.1     : ---------------------------------------------------------------------------------------------------- :    -
H1_AB028471.1      : ---------------ggtgcg-----actgcggctacgcttgatggtttattcaagaaagctggtgatggtca---------------------atcaat :  872
H12_AY337471.1     : ---------------ggtgcg-----actgcggctacgcttgatggtttattcaagaaagctggtgatggtca---------------------atcaat :  872
H51_AY250027.1     : ---------------ggtgcg-----gctactgcaactcttaaagagctgtttactcttgctggcgatggtaa---------------------atctct :  887
H45_AY250023.1     : ---------------attgca-----tcttcagcgaaattgtccgatctgtttgctagcggtagtaccttag---------------------------- :  853
H49_AY250026.1     : ---------------ggctcc-----actccagcaacgatgtcttccctgtttaaggccgcggatgacaaagatgccgctcaat---------------- :  862
H23_AB028476.1     : ---------------atgacg-----actcaagcaacaactaccgatttggttactgctgctg---------------------------------catc :  884
H7_AY337468.1      : ---------------agcgca-----gctaaagctgatatgaaagcgctgctcaaagcagcgagcgaaggta---------------------------- :  892
H6_AY249991.1      : ---------------ggtgat-----acacaagctactttggctaaacttgctactgctactggtgctaaag---------------------------- :  895
H18_AY250001.1     : ---------------actgat-----actcaagctaaactgtctgacttaatggcaaacaatgctaatgcca---------------------------- :  910
```

```
H34_AY250016.1    : ---------------aacccg-----aaaaaagcaaccgtttctgggcttctcggaaatacggatgcgaaaggta------------------------- :  895
H14_AY249998.1    : ---------------gcttct--ttgactgaggctagtctgtccacacttg---------------------------------------------cagc :  860
H15_AY249999.1    : ttgacc---------tctgct-----aaactggctactctgactggccttc---------------------------------------------aggg :  911
H20_AY250003.1    : aacggt---gttggaacactc-----gcggaagcgactctgagtggtttagctctgaacaaaaatggtttaa---------------------------- :  907
H31_AY250013.1    : ---------------acttta-----gaagaagctaccctcaatggcttagctttcaaccactctggtccag---------------------------c :  878
H28_AY250010.1    : ---------------actgtc-----aaagcagccactcttgatgcactgactaaaaactggcatacaacaggca---------------------cacc :  923
H46_AY250024.1    : ---------------actgta-----acagcagcaaccctggatggactgaccaaaaaccatgatgcgacag---------------------------- :  922
H44_AB269770.1    : gc------------tggtatt-----aagcctgcaactcttgatgctcttactcaaaacacaactactcctg---------------------------c :  944
H19_AY250002.1    : ---------------acaacc-----aaagctgcaacctgggataacctgatggccaatacagatactacaggcaaagatg---cctatggtaactctgc :  917
H55_AB269771.1    : ---------------actact-----attgcagctacttgggacaatctgatggcaaacaccgataccacaggtgttgatgctacttctggtcattcagc :  923
H26_AY250008.1    : ttgggg---------gctgct-----gataaagcaactgtagataaactgtttgctggtg---------------------------------------- :  895
H30_AY250011.1    : ---------------ggtcca-----gatgcggcaacgttggacggtattttcaacggtgcgaatggtaatg---------------------------- :  922
H32_AY250014.1    : ---------------ggccca-----gccgcggcaacgttggacggtattttcaacggtgcgaatggtcatg---------------------------- :  922
H37_AY250017.1    : ---------------acttta-----actcaggcaacattgaatgatgtccttactggtgctaatt---------------------------------- :  895
H41_AY250020.1    : ---------------tcacca-----cctgcagcaacgctggataacgtattagcttccg---------------------------------------- :  889
H9_AY249994.1     : ---------------aacgat-----accgcagcgacttggatggtttaatttccaacagcgattctaccggtgcggttc-------------------c :  902
H36_EF392693.1    : ---------------ggcggc-----aaagcagcaactttagaaaatctagcgctcaataagacaggtacca---------------------------a :  890
                                                       a

                         *     1120         *     1140         *     1160         *     1180         *     1200
H21_AIHL01000060.1: ----------gggagtgctgatacatcaggtactagtg-------------------------------------------------------------- :  781
H8_AJ865465.1     : ----------gacagtaatgcacccacgggtgccggcg-------------------------------------------------------------- :  784
H40_AJ884568.1    : ----------aacagtacagccgccacgggtgccgacg-------------------------------------------------------------- :  784
H11_AY337465.1    : -------------------acagatgctgacaaaaaag-------------------------------------------------------------- :  769
H27_AM231154.2    : -------------------gatgcgacagggaaggacg-------------------------------------------------------------- :  778
H2_AIHA01000023.1 : ----------aagggggctattgatgttcagttgggta-------------------------------------------------------------- :  751
H16_AY337475.1    : -----------------atggttct---------gcca-------------------------------------------------------------- :  790
H3_AB128916.1     : -----------------gtaccgctgctcataacgctc-------------------------------------------------------------- :  793
H54_AB128918.1    : cacacaatctgttgacgctactgttagtgcaggagcta-------------------------------------------------------------- :  787
H53_AB128917.1    : -----------------aggaagtatataaaaaggttg-------------------------------------------------------------- :  697
H47_EF392694.1    : ---------------------------------------------------------------------------------------------------- :    -
H35_EF392692.1    : --agagcggtaagtatcatatcagtaaagataatggtg-------------------------------------------------------------- :  802
H43_AIGA01000038.1: -----------------ccacgccaggtacggctgttg-------------------------------------------------------------- :  727
H17_CP002291.1    : -----------------caacgtccactccagtcgctg-------------------------------------------------------------- :  739
H5_AY249990.1     : -----------------ctgcggctactcagaaagatc-------------------------------------------------------------- :  709
H56_AY250029.1    : -----------------ctgcagctactcagaaagatc-------------------------------------------------------------- :  709
H42_AY250021.1    : -----------------ccggcgctaccaaagatgata-------------------------------------------------------------- :  694
H29_AY250012.1    : -----------------cgaatgccacccagattggta-------------------------------------------------------------- :  685
H38_AY250018.1    : -----------------ataccgcaacgcagtcaggcc-------------------------------------------------------------- :  694
H33_AY250015.1    : -----------------ctaccggttccatgactggta-------------------------------------------------------------- :  682
H39_AY250019.1    : -----------------ctggcgcgactcagaacggtc-------------------------------------------------------------- :  685
H52_AY250028.1    : -----------------ccacagcaacacagacaggtc-------------------------------------------------------------- :  712
H4_AJ536600.1     : -----------------tcgttgattctgcaaccggta-------------------------------------------------------------- :  553
H25_AGSG01000116.1: -----------------tcgttgattctgcaaccggta-------------------------------------------------------------- :  553
H10_AY249995.1    : -----------------ctactgttggccgcgatgtaa-------------------------------------------------------------- :  661
H24_AY250006.1    : -----------------atgacttcacagacgctggtc-------------------------------------------------------------- :  706
```

```
H48_AY250025.1    : -----------------caactatcacttcaggcggta------------------------------------------------------------ :  712
H1_AB028471.1     : cgggtttaataagactgcatcagtcacgatggggggaa------------------------------------------------------------ :  910
H12_AY337471.1    : cgggtttaagaagactgcatcagtcacgatggggggaa------------------------------------------------------------ :  910
H51_AY250027.1    : ggggaacggcggtactgctaccgttactctggataata------------------------------------------------------------ :  925
H45_AY250023.1    : --------cgacaactggttctatccagctgtctggca------------------------------------------------------------ :  883
H49_AY250026.1    : -----------------cctcgattgattttggcggga------------------------------------------------------------ :  883
H23_AB028476.1    : tgctactggtaagggtggatccctgacctttggtgaca------------------------------------------------------------ :  922
H7_AY337468.1     : --------gtgacggtgcctctctgacattcaatggca------------------------------------------------------------ :  922
H6_AY249991.1     : -----------ccgcgaccatccaaactgataaaggaa------------------------------------------------------------ :  922
H18_AY250001.1    : -----------aaacagtcattacaacagataaaggta------------------------------------------------------------ :  937
H34_AY250016.1    : --ctgctgttaaaacaaccatcaagacagaggctggtg------------------------------------------------------------ :  931
H14_AY249998.1    : caataataccaaagcgacaaccattgacattggcggta------------------------------------------------------------ :  898
H15_AY249999.1    : ctctggtgttgcttcaaccatcactactgaagatggca------------------------------------------------------------ :  949
H20_AY250003.1    : --cggctgttaaatccacaattactacagctgataaca------------------------------------------------------------ :  943
H31_AY250013.1    : cgctgctgtacaatctactattactactgcggatggaa------------------------------------------------------------ :  916
H28_AY250010.1    : gagtgccgtatctacggtaattacaactgaagatgaaa------------------------------------------------------------ :  961
H46_AY250024.1    : --gagctgttggtgttgatatcacgaccgcagatggcg------------------------------------------------------------ :  958
H44_AB269770.1    : ggcggcagttccagtcaccattacgaccgaagataaga------------------------------------------------------------ :  982
H19_AY250002.1    : ggcagcagctgttgggacagtaatcgaagcaaaaggaa------------------------------------------------------------ :  955
H55_AB269771.1    : tgcaactgctgtaaaaaccatcattaaagtcaaagata------------------------------------------------------------ :  961
H26_AY250008.1    : -----ctcaggatgcaacgatcaccttcgatagcggca------------------------------------------------------------ :  928
H30_AY250011.1    : --cagcagttgatgcgaagattacattcggcagcggca------------------------------------------------------------ :  958
H32_AY250014.1    : --atgcagttgatgcgaagattaccttcggcagcggca------------------------------------------------------------ :  958
H37_AY250017.1    : --cagttgatgatacaaggattgacttcgatagcggca------------------------------------------------------------ :  931
H41_AY250020.1    : --caactgtaaatgccactatcaagtttgatagcggta------------------------------------------------------------ :  925
H9_AY249994.1     : agttggggttgcaactacaattacaattacttctggtacagcttccggaatgtctgttcagtccgcaggagcaggaattcagacctcaacaaattctcag : 1002
H36_EF392693.1    : cactgcggttaagtcctctattactaccgaatcaggca------------------------------------------------------------ :  928
                                                    g

                           *     1220         *     1240         *     1260         *     1280         *     1300
H21_AIHL01000060.1: ----------------------------------------------------caaccgttgcccctacgacagccctatacaaaaa-------------- :  815
H8_AJ865465.1     : ----------------------------------------------------caacaataactacagacacagctgtttacaaaaa-------------- :  818
H40_AJ884568.1    : ----------------------------------------------------caacaataactacagacatagatgtttacaaaaa-------------- :  818
H11_AY337465.1    : ----------------------------------------------------cttctttgattacgagtgagacagtttacaaaaa-------------- :  803
H27_AM231154.2    : ----------------------------------------------------caacattaaaaacgtctgatcctgtttacaaaaa-------------- :  812
H2_AIHA01000023.1 : ----------------------------------------------------ctgataccgcaaccgcttctatcacaggtgcaaaa------------- :  786
H16_AY337475.1    : -------------------------------------------------catcagttgatttagctgcgggtaaaatgggtaaagcattagcttataatg :  841
H3_AB128916.1     : -------------------------------------------------tttctgttgatctacagaaaggaactgttggaaaagcattatctttcaacg :  844
H54_AB128918.1    : ----------------------------------------------------tttctgaattgaaaacgggtggtgttgataatacggcagctggtaatg :  835
H53_AB128917.1    : ----------------------------------------------------cagcaaatacatctggacttgctgctaaccaaaca------------- :  732
H47_EF392694.1    : ---------------------------------------------------------------------------------------------------- :    -
H35_EF392692.1    : ----------------------------------------------------caacctatgctaatgcaactgtgaatgccactac-------------- :  836
H43_AIGA01000038.1: ----------------------------------------------------atgtcactgcggctaaaactgctctggc------------------- :  755
H17_CP002291.1    : ----------------------------------------------------taaccgctgcagctaacaccgccctgagcaatgc-------------- :  773
H5_AY249990.1     : ----------------------------------------------------aactgattaaagttgccgctgactctgacggctcggctgcggga---- :  753
H56_AY250029.1    : ----------------------------------------------------agctgattaaagttgccgctgactctgacggcgcggctgcggga---- :  753
```

```
H42_AY250021.1    : ----------------------------------------------------cgttgattaaagttgctgctaattctgacggagaggccgttggt---- :  738
H29_AY250012.1    : ----------------------------------------------------gtctggttcaggttggtgctgatgcaaacaatgatgcagttggt---- :  729
H38_AY250018.1    : ----------------------------------------------------agttagttcaggttggtgcagattctaccggtacgccaaaagca---- :  738
H33_AY250015.1    : ----------------------------------------------------agtacgttaaagttggagctgatgcattgggtgctgctgtaggt---- :  726
H39_AY250019.1    : ----------------------------------------------------agctgatcaaagtaacttctgacgccaacggtgcagctgttggt---- :  729
H52_AY250028.1    : ----------------------------------------------------agcctatcaaggtcacgacgaatagtgctggcgcggctgttggc---- :  756
H4_AJ536600.1     : ----------------------------------------------------aatacatggaagccactgtagccattaccggtacggcggcggcg---- :  597
H25_AGSG01000116.1: ----------------------------------------------------aatacatggcagccactgtagtcattaccagtacggcggcggcg---- :  597
H10_AY249995.1    : ----------------------------------------------------aattggatgcttctgcacttaaagccaaccaatcg------------- :  696
H24_AY250006.1    : ----------------------------------------------------aaaccgtttcctttggcactgatgctgc-------------------- :  734
H48_AY250025.1    : ----------------------------------------------------cacctgttcagattgataatactgcagg-------------------- :  740
H1_AB028471.1     : ----------------------------------------------------caacttataactttaaaacgggtgctgatgctgg-------------- :  944
H12_AY337471.1    : ----------------------------------------------------caacttataactttaaaacgggtgctgatgctga-------------- :  944
H51_AY250027.1    : ----------------------------------------------------ctacgtataatttcaaagctgctgcgaacgttactgatggtg------ :  967
H45_AY250023.1    : ----------------------------------------------------caacttataactttggtgcagcggcaac-------------------- :  911
H49_AY250026.1    : ----------------------------------------------------aaaaatacgaatttgctggtggcaattctactaa-------------- :  917
H23_AB028476.1    : ----------------------------------------------------cgacgtataaaattggtcagggtacggctggggt-------------- :  956
H7_AY337468.1     : ----------------------------------------------------cagaatataccatcgcaaaagcaactcctgcgacaaccactccag--- :  967
H6_AY249991.1     : ----------------------------------------------------cattcaccagtgacggtacagcgtttga-------------------- :  950
H18_AY250001.1    : ----------------------------------------------------catttactgctaatacgacaaagtttga-------------------- :  965
H34_AY250016.1    : ----------------------------------------------------taacagttacagctgaaggtaatacagg-------------------- :  959
H14_AY249998.1    : ----------------------------------------------------cctctatctcctttaccggtaatagtactacgccgaa----------- :  935
H15_AY249999.1    : ----------------------------------------------------ctaatattgatattgctg------------------------------ :  967
H20_AY250003.1    : ----------------------------------------------------cttcgattgtactgaatggttcaagcgatggtactggta--------- :  982
H31_AY250013.1    : ----------------------------------------------------cttcaatagttctagcaggttctggcgactttggaac----------- :  953
H28_AY250010.1    : ----------------------------------------------------caaccttcactctggctggcggtactga-------------------- :  989
H46_AY250024.1    : ----------------------------------------------------caactatctctctggcaggctctgctaacgcggcaac----------- :  995
H44_AB269770.1    : ----------------------------------------------------ctgaaatcaaattagcgggtgcaactgt-------------------- : 1010
H19_AY250002.1    : ----------------------------------------------------tgaccatcacttctgctggtggtaatgctcaggtgttaaaagacgcgg : 1003
H55_AB269771.1    : ----------------------------------------------------tgactattacttccgcaggtggtaatgctcaggtcgcaactgataaag : 1009
H26_AY250008.1    : ----------------------------------------------------tgacagctaaattcgatcaaactgctgg-------------------- :  956
H30_AY250011.1    : ----------------------------------------------------tgaccgttgatttcacccaggctagcaa-------------------- :  986
H32_AY250014.1    : ----------------------------------------------------tgaccgttgacttcacccaggttagcaa-------------------- :  986
H37_AY250017.1    : ----------------------------------------------------tgtctgtcacccttgataaagtgaacag-------------------- :  959
H41_AY250020.1    : ----------------------------------------------------tgacggttgattacactgcaggtactgg-------------------- :  953
H9_AY249994.1     : attcttgcaggtggtgcatttgcggctaaggtaagtattgagggaggcgctgctacagacattttggtagcaagtaatggaaacataacagcggctgatg : 1102
H36_EF392693.1    : ----------------------------------------------------ccaaaattgcaattgctggtagtactgacggcac-------------- :  962

                           *      1320      *      1340      *      1360      *      1380      *      1400
H21_AIHL01000060.1: ---------------------------------------------------------------------------------------------------- :    -
H8_AJ865465.1     : ---------------------------------------------------------------------------------------------------- :    -
H40_AJ884568.1    : ---------------------------------------------------------------------------------------------------- :    -
H11_AY337465.1    : ---------------------------------------------------------------------------------------------------- :    -
H27_AM231154.2    : ---------------------------------------------------------------------------------------------------- :    -
H2_AIHA01000023.1 : ---------------------------------------------------------------------------------------------------- :    -
```

```
H16_AY337475.1    : a---------------------------------------------------------------------------tgcaccaatgtctgtttattttgg :  866
H3_AB128916.1     : a---------------------------------------------------------------------------ttctcagatgtcagtatatgttga :  869
H54_AB128918.1    : ctaag----------------------------------------------------------------------------------------------- :  840
H53_AB128917.1    : ---------------------------------------------------------------------------------------------------- :    -
H47_EF392694.1    : ---------------------------------------------------------------------------------------------------- :    -
H35_EF392692.1    : ---------------------------------------------------------------------------------------------------- :    -
H43_AIGA01000038.1: ---------------------------------------------------------------------------------------------------- :    -
H17_CP002291.1    : ---------------------------------------------------------------------------------------------------- :    -
H5_AY249990.1     : ---------------------------------------------------------------------------------------------------- :    -
H56_AY250029.1    : ---------------------------------------------------------------------------------------------------- :    -
H42_AY250021.1    : ---------------------------------------------------------------------------------------------------- :    -
H29_AY250012.1    : ---------------------------------------------------------------------------------------------------- :    -
H38_AY250018.1    : ---------------------------------------------------------------------------------------------------- :    -
H33_AY250015.1    : ---------------------------------------------------------------------------------------------------- :    -
H39_AY250019.1    : ---------------------------------------------------------------------------------------------------- :    -
H52_AY250028.1    : ---------------------------------------------------------------------------------------------------- :    -
H4_AJ536600.1     : ---------------------------------------------------------------------------------------------------- :    -
H25_AGSG01000116.1: ---------------------------------------------------------------------------------------------------- :    -
H10_AY249995.1    : ---------------------------------------------------------------------------------------------------- :    -
H24_AY250006.1    : ---------------------------------------------------------------------------------------------------- :    -
H48_AY250025.1    : ---------------------------------------------------------------------------------------------------- :    -
H1_AB028471.1     : ---------------------------------------------------------------------------------------------------- :    -
H12_AY337471.1    : ---------------------------------------------------------------------------------------------------- :    -
H51_AY250027.1    : ---------------------------------------------------------------------------------------------------- :    -
H45_AY250023.1    : ---------------------------------------------------------------------------------------------------- :    -
H49_AY250026.1    : ---------------------------------------------------------------------------------------------------- :    -
H23_AB028476.1    : ---------------------------------------------------------------------------------------------------- :    -
H7_AY337468.1     : ---------------------------------------------------------------------------------------------------- :    -
H6_AY249991.1     : ---------------------------------------------------------------------------------------------------- :    -
H18_AY250001.1    : ---------------------------------------------------------------------------------------------------- :    -
H34_AY250016.1    : ---------------------------------------------------------------------------------------------------- :    -
H14_AY249998.1    : ---------------------------------------------------------------------------------------------------- :    -
H15_AY249999.1    : ---------------------------------------------------------------------------------------------------- :    -
H20_AY250003.1    : ---------------------------------------------------------------------------------------------------- :    -
H31_AY250013.1    : ---------------------------------------------------------------------------------------------------- :    -
H28_AY250010.1    : ---------------------------------------------------------------------------------------------------- :    -
H46_AY250024.1    : ---------------------------------------------------------------------------------------------------- :    -
H44_AB269770.1    : ---------------------------------------------------------------------------------------------------- :    -
H19_AY250002.1    : c---------ttataatgccgcatatgcgacctcaattactactggtactccgggtgatgcgggagccgcgggagccgctgcaactgcgggtaatgccgc : 1094
H55_AB269771.1    : c---------ttacaatgataaatacgctgctcgcattgttgctggt---------------gacaccgctgcacaggctgatacagcagcagatactgc : 1085
H26_AY250008.1    : ---------------------------------------------------------------------------------------------------- :    -
H30_AY250011.1    : ---------------------------------------------------------------------------------------------------- :    -
H32_AY250014.1    : ---------------------------------------------------------------------------------------------------- :    -
H37_AY250017.1    : ---------------------------------------------------------------------------------------------------- :    -
H41_AY250020.1    : ---------------------------------------------------------------------------------------------------- :    -
H9_AY249994.1     : gtagtgcactttatcttgatgcgactactggtggattcactacaacggctggaggaaatacagctgcttcgttagataatttaattgc---taacagtaa : 1199
```

```
H36_EF392693.1    : ---------------------------------------------------------------------------------------------------------------- :    -

                         *      1420         *      1440         *      1460         *      1480         *      1500
H21_AIHL01000060.1: ---------------------------------------------------------------------------------------------------------------- :    -
H8_AJ865465.1     : ---------------------------------------------------------------------------------------------------------------- :    -
H40_AJ884568.1    : ---------------------------------------------------------------------------------------------------------------- :    -
H11_AY337465.1    : ---------------------------------------------------------------------------------------------------------------- :    -
H27_AM231154.2    : ---------------------------------------------------------------------------------------------------------------- :    -
H2_AIHA01000023.1 : ---------------------------------------------------------------------------------------------------------------- :    -
H16_AY337475.1    : gggaa---------------------------------------------------------------------------------------aaaacctagatgtccaccaa :  891
H3_AB128916.1     : cggta---------------------------------------------------------------------------------------aaaaccttgaaattaagcag :  894
H54_AB128918.1    : ---------------------------------------------------------------------------------------------------------------- :    -
H53_AB128917.1    : ------------------------------------------------------------------------------------------ttta------------------ :  736
H47_EF392694.1    : ---------------------------------------------------------------------------------------------------------------- :    -
H35_EF392692.1    : ------------------------------------------------------------------------------------------------tggcgaagtaacctac :  852
H43_AIGA01000038.1: ---------------------------------------------------------------------------------------------tgcagcaggtgctgacacg :  774
H17_CP002291.1    : ------------------------------------------------------------------------------------------------------tggtgttact :  783
H5_AY249990.1     : ---------------------------------------------------------------------------------------------------------------- :    -
H56_AY250029.1    : ---------------------------------------------------------------------------------------------------------------- :    -
H42_AY250021.1    : ---------------------------------------------------------------------------------------------------------------- :    -
H29_AY250012.1    : ---------------------------------------------------------------------------------------------------------------- :    -
H38_AY250018.1    : ---------------------------------------------------------------------------------------------------------------- :    -
H33_AY250015.1    : ---------------------------------------------------------------------------------------------------------------- :    -
H39_AY250019.1    : ---------------------------------------------------------------------------------------------------------------- :    -
H52_AY250028.1    : ---------------------------------------------------------------------------------------------------------------- :    -
H4_AJ536600.1     : ---------------------------------------------------------------------------------------------------------------- :    -
H25_AGSG01000116.1: ---------------------------------------------------------------------------------------------------------------- :    -
H10_AY249995.1    : ---------------------------------------------------------------------------------------------------------------- :    -
H24_AY250006.1    : ---------------------------------------------------------------------------------------------aacagccggtatcagcact :  753
H48_AY250025.1    : ---------------------------------------------------------------------------------------------ttccgcaactgccaacctt :  759
H1_AB028471.1     : ---------------------------------------------------------------------------------------------tgctgcaactgctaacgca :  963
H12_AY337471.1    : ---------------------------------------------------------------------------------------------tgctgcaactgctaacgca :  963
H51_AY250027.1    : --------------------------------------------------------------------------------------------ctggtgtcatcgctgctgct :  987
H45_AY250023.1    : ------------------------------------------------------------------------------------------------------------ttct :  915
H49_AY250026.1    : ---------------------------------------------------------------------------------------------------------tggtggc :  924
H23_AB028476.1    : ---------------------------------------------------------------------------------------------------------tgatcct :  963
H7_AY337468.1     : --------------------------------------------------------------------------------------------tagctccgttaatccctggt :  987
H6_AY249991.1     : ---------------------------------------------------------------------------------------------------------------- :    -
H18_AY250001.1    : ---------------------------------------------------------------------------------------------------------------- :    -
H34_AY250016.1    : ------------------------------------------------------------------------------------------------------------tact :  963
H14_AY249998.1    : ---------------------------------------------------------------------------------------------------------------- :    -
H15_AY249999.1    : -----------------------------------------------------------------------------------------------------ctaacggtaat :  978
H20_AY250003.1    : --------------------------------------------------------------------------------------------atgctggtactgaaggtacg : 1002
H31_AY250013.1    : ---------------------------------------------------------------------------------------------aacaaaaactgctggggct :  972
H28_AY250010.1    : ---------------------------------------------------------------------------------------------tgctactacttctggtgca : 1008
```

```
H46_AY250024.1    : ---------------------------------------------------------------------------------aggtactcaatcaggtgca : 1014
H44_AB269770.1    : ---------------------------------------------------------------------------------tgctggtcagagtggtgca : 1029
H19_AY250002.1    : ggtgggagcgctgggcgcaa------------------------------------------------------------cggcagttgataataccacg : 1134
H55_AB269771.1    : agaagctaccgctactacta------------------------------------------------------------ctgcagtagccaacaccact : 1125
H26_AY250008.1    : ------------------------------------------------------------------------------------------------tacc :  960
H30_AY250011.1    : ------------------------------------------------------------------------------------------------aaaa :  990
H32_AY250014.1    : ------------------------------------------------------------------------------------------------caat :  990
H37_AY250017.1    : ------------------------------------------------------------------------------------------------cact :  963
H41_AY250020.1    : ---------------------------------------------------------------------------------------------------c :  954
H9_AY249994.1     : ggatgctaccttaaccgtaacttcaggtaccggccagaacactgtttatagcacaacaggaagtggcgctcagttcaccagtttagcaaaagtagacaca : 1299
H36_EF392693.1    : ---------------------------------------------------------------------------------------taccgcaggcaag :  975

                         *     1520       *     1540       *     1560       *     1580       *     1600
H21_AIHL01000060.1: ---------------------------------------------------------------------------------------------------- :    -
H8_AJ865465.1     : ---------------------------------------------------------------------------------------------------- :    -
H40_AJ884568.1    : ---------------------------------------------------------------------------------------------------- :    -
H11_AY337465.1    : ---------------------------------------------------------------------------------------------------- :    -
H27_AM231154.2    : ---------------------------------------------------------------------------------------------------- :    -
H2_AIHA01000023.1 : ---------------------------------------ctctttaagttagaagacgc------------caatggcaaagatactggttcatttgcgt :  835
H16_AY337475.1    : gtacaagatacccaagggaatcctgtacctaattcatttgctgctaaaacatcagacggc---------------------------------------- :  951
H3_AB128916.1     : gttctggacgctgatggtaagcctaaagcaggtgctttgcagctcaaaccgctgatggt----------------------------------------- :  954
H54_AB128918.1    : ---------------------------------------ttagtgaaaatgacctacac------------cgatagcaacggtaaaaaagtcgaaggcg :  889
H53_AB128917.1    : ---------------------------------------------------------------------------------------------------- :    -
H47_EF392694.1    : ---------------------------------------------------------------------------------------------------- :    -
H35_EF392692.1    : gattccggaacagcggctgcccttggtgctggt------------------------------------------------------------------- :  885
H43_AIGA01000038.1: agtggcctgaaa---------------------------ctggttcaactgtccaacac------------ggattccgcaggtaaagtgaccaacgtgg :  835
H17_CP002291.1    : ggttctgttgag---------------------------ttggttaaactcacttcaac------------tgattctgcaggcaatgctactgatgcag :  844
H5_AY249990.1     : ---------------------------------------tatgtaacattccaaggtaa------------aaactacgctacaacg------------- :  789
H56_AY250029.1    : ---------------------------------------tatgtaacattccagggtaa------------aaactacgctacaacg------------- :  789
H42_AY250021.1    : ---------------------------------------ttcgctaccgttcagggtaa------------gaattatgaaattacagat---------- :  777
H29_AY250012.1    : ---------------------------------------tttgttaccgtgcaggggaa------------aaactatgttgctaat------------- :  765
H38_AY250018.1    : ---------------------------------------ttcgtgtctgtccaaggtaa------------aagctttggcattgatgac---------- :  777
H33_AY250015.1    : ---------------------------------------tatgtcaccgtacagggaca------------aaacttcaaagctgatgct---------- :  765
H39_AY250019.1    : ---------------------------------------tacgtaaccattcagggtaa------------aaactatcaggctggtgcgaccggtgtt- :  777
H52_AY250028.1    : ---------------------------------------tatgttactattcaaggcaa------------agattaccttgctggtgcagacggtaag- :  804
H4_AJ536600.1     : ---------------------------------------------------------------------------------------------------- :    -
H25_AGSG01000116.1: ---------------------------------------------------------------------------------------------------- :    -
H10_AY249995.1    : ---------------------------------------cttgtcgtgtataaagataa------------aagcggcaatgat---------------- :  729
H24_AY250006.1    : ggtgcttct------------------------------ctcgttaaacttcaggatga------------gaaaggcaatgatactgctacttatgcaa :  811
H48_AY250025.1    : ggtgctgttagc---------------------------ttagtaaaactgcaggattc------------caagggtaatgataccgatacatatgcgc :  820
H1_AB028471.1     : ggggtatcgttcactgatacagctagcaaagaaaccgttttaaataaagtggctacagctaaacaaggcacagcagttgcagctaacggtgatacatccg : 1063
H12_AY337471.1    : ggggtatcgttcactgatacagctagcaaagaaaccgttttaaataaagtggctacagctaaacaaggcaaagcagctgcagctgacggtgatacatccg : 1063
H51_AY250027.1    : ggtgtaacttatacagccactgtttctaaagatgtcattctggcacaactgcaatctgcaagtcaggcagcagcaaccgctaccgacggtgatactgtcg : 1087
H45_AY250023.1    : ggcgtaacctacaccaaaactgtaagcgctgatactgtactgagcacagtgcagagtgc------------tgcaacggctaacacagcagttactggtg : 1003
H49_AY250026.1    : ggcgttaaattcaaagacacggtgtcttctgacgcgcttttggctcaggttaaagcgga------------------------tagtactgctaataatg : 1000
```

```
H23_AB028476.1    : gatgacgcttcagatgatgtactgggcaccatttcttactctaaatcagtaagcaagga---------tgttgttcttgctgatactaaagcaactggta : 1054
H7_AY337468.1     : gggattacttatcaggctacagtgagtaaagatgtagtattgagcgaaaccaaagcggc------------------------------tgccgcgacat : 1057
H6_AY249991.1     : -----tggtgcatcaatgtccattgataccaatacatttgcaaatgcagtaaaaaatga------------cacttatactgccactgtaggtgcta--- : 1030
H18_AY250001.1    : -----tggggtagatatttctgttgatgcttcaacgtttgctaacgccgttaaaaatga------------gacttacactgcaactgttggtgtaact- : 1047
H34_AY250016.1    : gtaaaaattgaaggtgctactgtttcagc------------atctgcatttacgggcat------------tgcatattccgccaacaccggtggga--- : 1036
H14_AY249998.1    : --cactattacttattcagtaacaggtgcaaaagttgatcaggcagctttcgataaagc------------tgtatcaacctctggaaacgatgttgatt : 1021
H15_AY249999.1    : attggtctgaccggtgttcgtatcagtgctgattctctgcagtcagcgactaaatctac------------gggctttactgttggtactggcgctacag : 1066
H20_AY250003.1    : attgctgttacaggcgctgtaattagttcagctgctctgcaatctgcaagcaaaacgac------------tggtttcactgttggtacagtagacacag : 1090
H31_AY250013.1    : attaatgtcacaggagcagtgatcagtgctgatgcacttctttccgccagtaaagcgac------------tgggtttacttctggcacttat------- : 1053
H28_AY250010.1    : atcactgtagcaaatgcaagaatgagtgctgagtctcttcaatcggcaactaagtccac------------aggattcacagttgatgttggagctactg : 1096
H46_AY250024.1    : attacactgaaaaatgttcgtatcagtgctgatgctctgcagtctgctgcgaaaggtac------------tgttatcaatgttgataatggtgctg--- : 1099
H44_AB269770.1    : attgtagttactggtgcccgaatcagcgctgaagctatgcagtctgctaccaaaacgac------------tggtttcacaactggcacaacgacagtag : 1117
H19_AY250002.1    : gcagatgttgccgatatctctatctcagcttcgcaaatggcgagcatccttcaggataa------------agatttcaccttaagtgatggtagtg--- : 1219
H55_AB269771.1    : gctgatgtttcaggggtaacaatctctgcgtctcaaatggcaagcattctgaaagacaa------------ggattttgcccttaatagtggaacta--- : 1210
H26_AY250008.1    : gttgatttcaaaggcgcgtctatttctgctgatgcaatggcatcaaccttaaataatgg------------ttcctatacagccaacgtaggtggta--- : 1045
H30_AY250011.1    : gtggatattaagggcgcaacggtatccgccgaagatatggacactgcgttaactgggca------------ggcttataccgtagctaacggcgcac--- : 1075
H32_AY250014.1    : gtggatattaagggcgcgacggtatccgccgaagatatgaacactgcgttaaccggtca------------ggcttataccgtagctaacggcgcac--- : 1075
H37_AY250017.1    : gtagatatcactggcgcatctatttcagccgctgcaatgactaatgagttgacaggtaa------------ggcctataccgtagtaaatggtgcag--- : 1048
H41_AY250020.1    : gcgaatattacaggtgcatccatttctgcagatgacatggccgcaaaactgagcggaaa------------ggcgtacactgttgccaatggtgctg--- : 1039
H9_AY249994.1     : gtcaatgtcaccaacgcacatgtcagtgccgaaggtatggcaaatctgacaaaaagcaa---------------ttttaccattgatatgggcggtacag : 1384
H36_EF392693.1    : atcagcgctactaatgtgaagattagtgctgaggatcttaaagctaaagcagatactgc------------gggttttactacgagtactggatttaca- : 1062

                         *     1620          *     1640          *     1660          *     1680          *     1700
H21_AIHL01000060.1: ------------------------------tagtgcagggcaattgactgca-acaaaagttgaaaataaagcag------------------------- :  859
H8_AJ865465.1     : ------------------------------cagtgcgggccagttcaccact-acaaaagtggaaaataaagccg------------------------- :  862
H40_AJ884568.1    : ------------------------------tagcgcgggccagttcactact-acaaaagtggaaaataaagccg------------------------- :  862
H11_AY337465.1    : ------------------------------tagcgctggcctttatacgaca-accaaagttgataacaaggctg------------------------- :  847
H27_AM231154.2    : ------------------------------tagtgcaggtcagttcactaca-actaaggttgaaaacaaagccg------------------------- :  856
H2_AIHA01000023.1 : -----------------------tgattggtgatgacggtaaacagtatgca-gcgaatgttgatcagaaaacag------------------------- :  886
H16_AY337475.1    : -----------------------------------------------------acctacattgcagtaaatgtag------------------------- :  973
H3_AB128916.1     : -----------------------------------------------------aagtctttagctgtcaatattg------------------------- :  976
H54_AB128918.1    : g-----------------ttacgcccttaaagttggtgatgactactatgca-gctgattatgaaagcactagca------------------------- :  946
H53_AB128917.1    : ---------------------------------------------------------------------------------------------------- :    -
H47_EF392694.1    : ---------------------------------------------------------------------------------------------------- :    -
H35_EF392692.1    : -----------------------------------------------------attgaagttggtagcgcactta------------------------- :  907
H43_AIGA01000038.1: g-----ttacggcc---------tgcagaatgacagcggcactatctttgca-accgactacgatggcaccactg------------------------t :  896
H17_CP002291.1    : g-----ttatggtc---------tgcaagcagccgatggcaaaatttacgct-acagattatgacggcaccaacg------------------------c :  905
H5_AY249990.1     : -----------------------------------------------------gtttcaacggcacttgatgata------------------------- :  811
H56_AY250029.1    : -----------------------------------------------------gctccagcggcgcttaatgatg------------------------- :  811
H42_AY250021.1    : -----------------------------------------------------ggtgtaaaaaaccagtccactg------------------------- :  799
H29_AY250012.1    : -----------------------------------------------------gactcattagtcaatgctaatg------------------------- :  787
H38_AY250018.1    : -----------------------------------------------------gccgccttgaagaataacactg------------------------g :  800
H33_AY250015.1    : -----------------------------------------------------ggcgcgctggttaactccaaga------------------------- :  787
H39_AY250019.1    : -----------------------------------------------------gacgttctggcgaacagcggtg------------------------- :  799
H52_AY250028.1    : -----------------------------------------------------gatgcaattgaaaacggtggtg------------------------- :  826
```

```
H4_AJ536600.1     : ---------------------------------------------------------------------------------------------------- :    -
H25_AGSG01000116.1: ---------------------------------------------------------------------------------------------------- :    -
H10_AY249995.1    : -----------------------------------------------------gcttatatcattcagaccaaag------------------------- :  751
H24_AY250006.1    : -----------------------tcaaagcacaagatggcagcctgtatgcc-gccaacgttgatgaggctaccg------------------------- :  862
H48_AY250025.1    : -----------------------ttaaagatacaaatggcaatctttacgct-gcggatgtgaatgaaactactg------------------------- :  871
H1_AB028471.1     : ------caacaattacctataaatctggcgttcagacgtatcaggcggtatttgccgcaggtgacggtactgctagcgcaaaatatgccgata------a : 1151
H12_AY337471.1    : ------caacaattacctataaatctggcgttcagacgtatcaggcggtatttgccgcaggtgacggtactgctagcgcaaaatatgccgata------a : 1151
H51_AY250027.1    : ------caacgatcaactataaatctggtgtcatgatcggttccgctacctttaccaatggtaaaggtactgccgatggtatgacttctggtacaactcc : 1181
H45_AY250023.1    : ------cgacaattaagtataatacaggtattcagtctgcaacggcgtccttcggtggtgtgaatactaatggtg------------------------- : 1072
H49_AY250026.1    : ------taaaaatcacctttaacaatggtcctctgtcattcactgcatcgttccaaaatggtg------------------------------------t : 1058
H23_AB028476.1    : acacgacaacagttgatttcaactccggtatcatgacttcaaaggttagtttcgatgcaggtacatcaactg---------------------------- : 1126
H7_AY337468.1     : ------cttcaattacctttaattccggtgtactgagcaaaactattgggtttaccgcgggtgaatccagtgatgctgcga------------------- : 1132
H6_AY249991.1     : --------------------------------------agacttatagcgta-acaacaggttctgctgctgcag------------------------- : 1066
H18_AY250001.1    : --------------------------------------ttacctgcgacatatacagtcaataatggcactgctg------------------------- : 1084
H34_AY250016.1    : --------------------------------------atacttatgctgtt-gccgcaaataatactacaaatg------------------------- : 1072
H14_AY249998.1    : ------tcac--------------------------taccgcaggttatagc-gtcga---cggcgcaactggcg------------------------- : 1060
H15_AY249999.1    : ------gtctg------------------------------------------accgt---aggtactgatggta------------------------- : 1090
H20_AY250003.1    : -----------------------------------------ctggttatatc-tctgt---aggtactgatggagtgttcaggcat----------atga : 1136
H31_AY250013.1    : -----------------------------------------------------accgt---aggtacagatggag------------------------- : 1072
H28_AY250010.1    : ------gtac--------------------------cagcgcaggcgatatt-aaagt---tgatagtaaaggtatagtacaacaac------------- : 1147
H46_AY250024.1    : --------------------------------------------atgatatt-tctgt---tagtaaaaccggtg------------------------- : 1126
H44_AB269770.1    : ------c------------------------------------------------------agctaatactggca------------------------- : 1132
H19_AY250002.1    : --------------------------------------atacttacaacgtg-accagcaatg------------------------------------- : 1243
H55_AB269771.1    : --------------------------------------ctcagtatgctgtc-accggcagtacaggtgccgtaa---cttacgatccagatacagatcc : 1268
H26_AY250008.1    : --------------------------------------aggcttatgccgta-accgc------------tggcg------------------------- : 1069
H30_AY250011.1    : --------------------------------------agtcttttgacgtt-gccgc---tggtggggcagtaa------------------------- : 1108
H32_AY250014.1    : --------------------------------------agtcttatgacgtt-gccgc---tgatggtgcagtaa------------------------- : 1108
H37_AY250017.1    : --------------------------------------aatcttacgctgta-gctac---taataacacagtaa------------------------- : 1081
H41_AY250020.1    : --------------------------------------agtcttatgacgtt-gctgcagttacgggggctgtaa------------------------- : 1075
H9_AY249994.1     : ------gtacagta---------------------------------------acttacacagtttccaatgggg------------------------- : 1414
H36_EF392693.1    : -----------------------------------------------------gtggctgctggtggtgatcaga------------------------- : 1084

                          *     1720        *     1740        *     1760        *     1780        *     1800
H21_AIHL01000060.1: --cgacactatctgatcttgatc------tgaacgctgccaagaaaacaggaagcacgttagttgttaacggtgcaacttacgatgttagtgcagatggt :  951
H8_AJ865465.1     : --caacactctctgatctggatc------ttaatgcagccaagaaaacaggtagcactttagttgtaaatggcgccacctacaatgtcagcgcagatggt :  954
H40_AJ884568.1    : --caacactctctgatctggatc------taaatgctgccaagaaaacaggtagtactttagttgtaaatggcgccacctacaatgtcagtgcagatggt :  954
H11_AY337465.1    : --ccacactttccgatcttgatc------tcaatgcagctaagaaaacaggaagcacgttagttgttaacggtgcaacttacgatgttagtgcagatggt :  939
H27_AM231154.2    : --ctacagcatcggatctggact------taaataacgctaaaaaagtgggtagttctttagttgtaaatggcgctgattatgaagttagcgctgatggt :  948
H2_AIHA01000023.1 : --gagcagtttccgttaaaacaa---------tgtcttacactgatgctgacggtgtcaaacacgacaatgttaaagttgaactgggtggaagcgatggc :  975
H16_AY337475.1    : --atgccgctacaggtaacacgt---------ctgttattactgatcctaatggtaa---------------ggcagttgaatgggcagtaaaaaatgat : 1047
H3_AB128916.1     : --atggcaac---ggcaatactt---------ctgttgtgaaagatgcagacggcaa---------------taatgtcgaatgggttgttgataaagac : 1047
H54_AB128918.1    : --aaaccgtgactgttcgtacaa---------cttcctataaagatgttgatggagtaccacag---aagggtttaaataaaatcggtggcgcggacggt : 1032
H53_AB128917.1    : --------------------------------aatcttacaaaaaagataatggcga------------------------------------------- :  761
H47_EF392694.1    : --------------------------------aagtaacagtggatcttgctgatgcagca--------------------------------------- :  639
H35_EF392692.1    : --aagagattgctaaaccagacg------ctggggtggctgtggaccttactggtgtttctggtgttacaggtgcacaattattcgcgaaaaaggatggt :  999
```

```
H43_AIGA01000038.1: gaccacgccgggcgcagagactg---------tgacttacaaagatgcttccggtaa---cagcaccactgcggctgtcacactgggtggctctgatggc :  984
H17_CP002291.1    : aactgcattcactgctaaaactg---------ctacttatactgatgcttctggtaatactcagac---ttcagcaatcactctgggcggcacagatggc :  993
H5_AY249990.1     : --atactgcggcaaaagcaacag---------ataataaagttgttgttg------------------------------------------------------ :  850
H56_AY250029.1    : --acactacggcaacagccacag---------cgaacaaagttgttgttg------------------------------------------------------ :  850
H42_AY250021.1    : --ctgcaccaaccgatattgctc------------------------------------------------------------------------------ :  820
H29_AY250012.1    : --gcgctgctggcgctgcagcaa------ctagagttacaattgatggtgatggtag--------------------------ccttggagctaaccaggct :  855
H38_AY250018.1    : tgatgctaccgctactcaaccgg---------gaacatctgggacaacagttgtcgc-------------------------------agcgtcaattcatct :  863
H33_AY250015.1    : --atgctgctggtagtcagaatg---------ttacttctgcaattggcg------------------------------------------------------ :  826
H39_AY250019.1    : --ttgcagctccaactacagctg---------ttgataccggtactctgc------------------------------------------------------ :  838
H52_AY250028.1    : --acgctgcaacaaatgaagaca------caaaaatccaacttaccgatg------------------------------------------------------ :  868
H4_AJ536600.1     : ---------------------------------------------------------------------------------------------------- :    -
H25_AGSG01000116.1: ---------------------------------------------------------------------------------------------------- :    -
H10_AY249995.1    : --atgtaacaactaatcaatcaactttcaatgccgctaatatcagtgatgctggtgt--------------------------------------------- :  806
H24_AY250006.1    : --gtaaagtcactgtcaaaaccg---------ccagctatactgatgctgacggcaaagcagtgaccgatgccgctgtaaaactgggtggtgacaatggc :  951
H48_AY250025.1    : --gtgctgtttctgttaaaacta---------ttacctatactgactcttccggtgccgccagttctccaaccgcggtcaaactgggcggagatgatggc :  960
H1_AB028471.1     : tactgacgtttctaatgcaacag---------caacatacacagatgctgatggtga-------------------------aatgactacaattggttca : 1218
H12_AY337471.1    : agctgacgtttctaatgcaacag---------caacatacactgatgctgatggtga-------------------------aatgactacaattggttca : 1218
H51_AY250027.1    : agtcgtagctacaggtgctaaag---------ctgtatatgttgatggcaacaatga-------------------------actgacttccactgcatct : 1248
H45_AY250023.1    : --------ctggtaattcgaatg---------acacctatactgatgcagacaaaga-------------------------gctcaccacaaccgcatct : 1131
H49_AY250026.1    : atctggctccgcggcatcgaatg---------cagcctacattgatagcgaaggcga-------------------------actgacaactactgaatcc : 1125
H23_AB028476.1    : --------------------------------atacattcaaagatgcagatggtgc-------------------------tatcaccaaaactaaagaa : 1170
H7_AY337468.1     : --------------------------------agtcttatgtggatgataaaggtgg-------------------------tatcactaacgttgccgac : 1176
H6_AY249991.1     : -----------------------------acaccgcttatatgagcaatggggttct---------------------------------------cagt : 1098
H18_AY250001.1    : -----------------------------catcagcgtatttagtcgatggaaaagt---------------------------------------gagc : 1116
H34_AY250016.1    : --------------------------------gtttcctggcggggatga-------------------------------cttaacccaggatgctcaa : 1110
H14_AY249998.1    : --ctgtaacaaaaggtgttgctc---------cggtttatattgataacaacggggc-------------------------gttgaccacat------ct : 1119
H15_AY249999.1    : --aagtgactatcggcgggacta---ctgctcagtcctacaccagcaaagatggttc-------------------------cctgactactg------at : 1155
H20_AY250003.1    : tgctgcgacttctggcaacaaag---------cttcttacaccaacactgacggtac-------------------------actgactactg------at : 1197
H31_AY250013.1    : --ttgttaaatctggtggcaatg---------acgtttataacaaagctgacgggac---------------------gggattaactactg------ac : 1134
H28_AY250010.1    : --acacaggtacaggttttgaag---------acgcttacaccaaagctgatggttc-------------------------actgactaccg------at : 1206
H46_AY250024.1    : --tcgttactaccggaggtgcgc---------ctacttatactgatgctgatggtaa-------------------------attaacgacaa------cc : 1185
H44_AB269770.1    : --aagttacaattggtggtaatc---------aagcttacactcagactgacggtac-------------------------gttagctgcca------ag : 1191
H19_AY250002.1    : --ctgtcactatcaatggcaaag---------cagcaaacattgatgacagcggcgc-------------------------aatcacagaccaaaccagt : 1308
H55_AB269771.1    : tgccgcgactggtgatattgttt---------ctgcttatgttgatgatgcaggtac-------------------------attgacaactgatgcaaac : 1335
H26_AY250008.1    : --cagttcagacaggtggcgcag---------atgtgtataaagataccactggcgc-------------------------actgacgactgaagatgac : 1134
H30_AY250011.1    : --ccgctactacaggtggcgcta---------ccgtaaatattggtgctgatggtga-------------------------actgacgactgcgaccaac : 1173
H32_AY250014.1    : --ctgctactacaggtggagcga---------ccgtaaatattggtgctgagggtga-------------------------actgacgactgcggccaac : 1173
H37_AY250017.1    : --aaacgactgctgatgctaaaa---------atgtttatgttgatgctagtggtaa-------------------------attaactactgatgacaaa : 1146
H41_AY250020.1    : --caactacagcaggtaattcac---------ctgtgtatgccgatgcagacggtaa-------------------------attaacgacgagtgccagt : 1140
H9_AY249994.1     : --atgtgaaagctgctgcaaatg------ctgatgtttatgtcgaagatggtgc---------------------------actttcagccaatgctaca : 1479
H36_EF392693.1    : --aagcaacccttaacggttctg---------aggcatacgttaaaggcgatggatt------------------------------------tactatcgac : 1140

                         *      1820         *      1840         *      1860         *      1880         *      1900
H21_AIHL01000060.1: aaaacgataacg----------------------------------------------------------gagactgcttctggta--------------- :  979
H8_AJ865465.1     : aaaacggtaact----------------------------------------------------------gatactactcctggtg--------------- :  982
```

```
H40_AJ884568.1    : aaaacggtaact---------------------------------------------------------gatactactcctggtg--------------- :  982
H11_AY337465.1    : aaaacgataacg---------------------------------------------------------gagactgcttctggta--------------- :  967
H27_AM231154.2    : aagacagtaact---------------------------------------------------------gggcttg------------------------ :  967
H2_AIHA01000023.1 : aaaaccgaagtt---------------------------------------------------------gtaactgcaaccgatg--------------- : 1003
H16_AY337475.1    : ggttctgc------acaggcaattatgcgtgaagat---------------------------------gataaggtttatacag--------------- : 1093
H3_AB128916.1     : ggtgcagc------taagactgttgtacgtaaagat---------------------------------gacaaaatatacggcg--------------- : 1093
H54_AB128918.1    : aaaaccgaaaca---------------------------------------------------------gtaaccataggtgaaa--------------- : 1060
H53_AB128917.1    : ---act--------------------------------------------------------------tggatatgtggttgaaa--------------- :  781
H47_EF392694.1    : ---------------------------------------------------------------------ggcgatttaactaaaa--------------- :  655
H35_EF392692.1    : agc------------------------------------------------------------------ggttatgtaattaaag--------------- : 1018
H43_AIGA01000038.1: aaaaccaatctg---------------------------------------------------------gttaccgccgctgacg--------------- : 1012
H17_CP002291.1    : aaaactgagctg---------------------------------gtaactctt---------------ggaagtggtgctaacg--------------- : 1030
H5_AY249990.1     : ---------------------------------------------------------------------------------------------------- :    -
H56_AY250029.1    : ---------------------------------------------------------------------------------------------------- :    -
H42_AY250021.1    : ---------------------------------------------------------------------------------------------------- :    -
H29_AY250012.1    : aaaattga-------------------------------------------------------------------------------------------- :  863
H38_AY250018.1    : gagtacgg-------------------------------------------------------------------------------------------- :  871
H33_AY250015.1    : ---------------------------------------------------------------------------------------------------- :    -
H39_AY250019.1    : ---------------------------------------------------------------------------------------------------- :    -
H52_AY250028.1    : ---------------------------------------------------------------------------------------------------- :    -
H4_AJ536600.1     : ---------------------------------------------------------------------------------------------------- :    -
H25_AGSG01000116.1: ---------------------------------------------------------------------------------------------------- :    -
H10_AY249995.1    : ----------------------------------------------------------------------------------------tttat------- :  811
H24_AY250006.1    : acaaccgaaattg------------------------------------------------------ttgtcgatgctgcgtcag--------------- :  982
H48_AY250025.1    : aaaacagaagtg---------------------------------------------------------gtcgatattgatggtaaaacatacg------ :  997
H1_AB028471.1     : tacaccac------gaagtattcaatcgatgctaacaacggcaaggtaactgttgattct---------ggaactggtacgggta--------------- : 1288
H12_AY337471.1    : tacaccac------gaagtattcaatcgatgctaacaacggcaaggtaactgttgattct---------ggaactggtacgggta--------------- : 1288
H51_AY250027.1    : tacgatac------gacttactctgtcaacgcagatacaggcgcagtaaaagtggtatca---------ggtactggtactggta--------------- : 1318
H45_AY250023.1    : tacactat------caactacaacgtcgataaggataccggtacagtaactgtagcttcaaatg---gcgcaggtgcaactggta--------------- : 1207
H49_AY250026.1    : tacaacac------aaattattccgtagacaaagacacgggggctgtaagtgttacaggg---------gggagcggtacgggta--------------- : 1195
H23_AB028476.1    : tacaccac------ttcttatgctgtaaataaagatactggtgaagttaccgttgctgattatgctgcggtagatagcgccgataaggctgttgatgata : 1264
H7_AY337468.1     : tatacagt------ctcttacagcgttaacaaggataacggctctgtgactgttgccgggtatgcttcagcgactgataccaataaag------------ : 1258
H6_AY249991.1     : gatactccgcc---aacttactatgcacaagctgat---------------------------------ggaagtatcacaacta--------------- : 1147
H18_AY250001.1    : aaaactcctgc---cgagtattttgctcaagctgat---------------------------------ggcactattactagtg--------------- : 1165
H34_AY250016.1    : actgtttc------aacctactactcgcaagccgat---------------------------------ggcacggtcacgaata--------------- : 1156
H14_AY249998.1    : gatactgt------agatttttatctacaggatgat---------------------------------ggttcagtgactaacg--------------- : 1165
H15_AY249999.1    : aacaccac------taaactgtatctgcagaaagat---------------------------------ggctctgtaaccaacg--------------- : 1201
H20_AY250003.1    : aacaccac------taaactgtatctgcagaaagat---------------------------------ggctctgtaaccaacg--------------- : 1243
H31_AY250013.1    : aataccac------aaaatattatttacaagatgac---------------------------------gggtctgtaactaatg--------------- : 1180
H28_AY250010.1    : aatacaac------caatctgtttttgcaaaaagac---------------------------------ggaactgtgaccaatg--------------- : 1252
H46_AY250024.1    : aacaccgt------tgattatttcctgcaaactgat---------------------------------ggcagcgtaaccaatg--------------- : 1231
H44_AB269770.1    : aatgaaac------tgagattttcctgcagaaagac---------------------------------ggctccattactaaca--------------- : 1237
H19_AY250002.1    : aaagttgt------caattatttcgctcatactaac---------------------------------ggtagcgtgactaacg--------------- : 1354
H55_AB269771.1    : aaaactgt------aaaatattatgcccacactaat---------------------------------ggtagcgtcacgaacg--------------- : 1381
H26_AY250008.1    : gaaaccgttaccgcgacctactacggttttgctgat---------------------------------ggtaaagtttctgacg--------------- : 1186
H30_AY250011.1    : aagactgtcacagaaacttatcacgaatttgctaac---------------------------------ggcaatattctggatg--------------- : 1225
```

```
H32_AY250014.1    : aagactgtcacagaaacttatcacgaatttgctaac---------------------------------ggcaatattctggatg--------------- : 1225
H37_AY250017.1    : gccactgttacagaaacttatcatgaatttgcgaat---------------------------------ggcaatatctatgatg--------------- : 1198
H41_AY250020.1    : aatacggttactcagacttatcacgagtttgctaat---------------------------------ggtaacatttatgatg--------------- : 1192
H9_AY249994.1     : aaagatgt------aacctactttgaacaaaaaaat---------------------------------gggctattaccaaca--------------- : 1525
H36_EF392693.1    : aacactgc------aaaatattacgtgcaagaagac---------------------------------ggtgctatcaccaacg--------------- : 1186
                                                                                           g

                         *     1920          *     1940          *     1960          *     1980          *     2000
H21_AIHL01000060.1: ---acaataaagtcatgtatctgagcaaatcagaaggtggtagcccgattctggtaaacgaagatgcagcaaaatcgttgcaatctaccaccaacccgct : 1076
H8_AJ865465.1     : ---cccctaaagtgatgtatctgagcaaatcagaaggtggtagcccgattctggtaaacgaagatgcagcaaaatcgttgcaatctaccaccaacccgct : 1079
H40_AJ884568.1    : ---cccctaaagtgatgtatctgagcaaatcagaaggtggtagcccgattctggtaaacgaagatgcagcgaaatcgttgcaatctaccaccaacccgct : 1079
H11_AY337465.1    : ---acaataaagtcatgtatctgagcaaatcagaaggtggtagcccgattctggtaaacgaagatgcagcaaaatcgttgcaatctaccaccaacccgct : 1064
H27_AM231154.2    : ------gcaaaactatgtatctgagcaaatcagaaggtggtagcccgattctggtaaaagaagatgcagcaaaatcgttgcaatctactaccaacccgct : 1061
H2_AIHA01000023.1 : ---gcaaaactt------acagtgttagtgatttacaaggtaagagcctgaaaactgattctattgcagcaatttctacgcagaaaacagaagatccttt : 1094
H16_AY337475.1    : ---ccaatatcacg------aataagacggcaaccaaaggtgctga---------actcagtgcctcagatttgaaagccttagcaaccacaaatccatt : 1175
H3_AB128916.1     : ---cttcagtaacagggtttggtgggactccaactgttaacgttgatacaaccgcaattgatgcttctgagttaaaaggcatgacaactgctaagccact : 1190
H54_AB128918.1    : ---aaacttatg------ccgctgataaattaaagatcatgacttcagtaaacaagctaccttaggtgaagaagcaacaacaactactgtaaacccatt : 1151
H53_AB128917.1    : ---acgctgatg------gcacttttaaccgtgctaacgtcgattcaaaaacgggtgttgtttctgtaggaactaaaatttccacaagccctgatgtttt :  872
H47_EF392694.1    : c------------------------------------------------caaagttgtagatgaagatgcaacagcagcaaccaaaactagtaacccttt :  707
H35_EF392692.1    : ---gcactgctg------ataataaagaagtactgttcgaagcaaaagttgctgctgatggcaaagtgactaagggtgatcaacttacagcagatcccct : 1109
H43_AIGA01000038.1: ---gcaaaacgt------acggtgcgactgcactgaatggtgctgatctgtccgatcctaataacaccgttaaatctgttgcagacaacgctaaaccgtt : 1103
H17_CP002291.1    : ---cgaagactt------atactgcctctgatttggacggtgcagatctccatgacccggcgaatactgtcaagcctattgctgataactcaaaaccgct : 1121
H5_AY249990.1     : ------------------------------------------aattatcaacagcaaaaccgactgcacagttctcagggcttcttctgctgatccact :  908
H56_AY250029.1    : ------------------------------------------aattatctacagcaactccgactgcgcagttctcagggcttcttctgctgatccact :  908
H42_AY250021.1    : ------------------------------------------agaccattgatctggatacggctgatgaatttactggggcttccactgctgatccact :  878
H29_AY250012.1    : ---acttagcc-------------------------------aaaatggtgctactgctgcaacatcagagttcgctggtgcttcaaccaacgatccact :  929
H38_AY250018.1    : ---gcaaaaactc---------------------------tgtagacgctgatgtaacggcttccactgaattcacaggtgcttcaaccaacgatccact :  941
H33_AY250015.1    : ------------------------------------------atattgctaataaagcgaatgctaacatttacactggaacctcttctgcagatccact :  884
H39_AY250019.1    : ------------------------------------------aactgagcggtactggtgcaactactgagctgaaaggtactgcaactcagaacccact :  896
H52_AY250028.1    : ----------------------------aactcgatgttgatggttctgtaaaaacagcggcaacagcaacatttctggtactgcaaccaacgatccgct :  941
H4_AJ536600.1     : --------------------------------------------------gtaactgttggtgcagcggaagtggcgggagccgctacagccgatccgtt :  647
H25_AGSG01000116.1: --------------------------------------------------gtaactgttggtgcaacggaagtggcgggagccgctacagccgaaccgtt :  647
H10_AY249995.1    : ---------------------------------------------------ctattggtgcatctacaaccgcgccaagcaatttaacagctaacccgct :  860
H24_AY250006.1    : ---gtaaaacttacgatgctggtgcactgcaaaacgttgatctctccagtgcaaccaacacggtaaccgcaatcccgaacggtaaaaccacgtctccgct : 1079
H48_AY250025.1    : ---attctgccgatttaaatggcggtaatctgcaaacaggtttgactgctggtggtgaggctctgactgctgttgcaaatggtaaaaccacggatccgct : 1094
H1_AB028471.1     : ---aatatgcgccgaaagtcggggctgaagtatatgttagtgctaatggtactttaacaacagatgcaactagcgaaggcacagtaacaaaagatccact : 1385
H12_AY337471.1    : ---aatatgcgccgaaagtaggggctgaagtatatgttagtgctaatggtactttaacaacagatgcaactagcgaaggcacagtaacaaaagatccact : 1385
H51_AY250027.1    : ---aatttgaagctgttgctggtgcggatgcttatgtaagcaaagatggcaaattaacgacagaaaccaccagtgcaggcactgcaaccaaagatccttt : 1415
H45_AY250023.1    : ---aatttgcagctactgttggggcacaggcttatgttaactctacaggcaaactgaccactgaaaccaccagtgcaggcactgcaaccaaagatcctct : 1304
H49_AY250026.1    : ---aatacgccgcaaacgtgggtgctcaggcttatgtaggtgcagatggtaaattaaccacgaatactactagtaccggctctgcaaccaaagatccact : 1292
H23_AB028476.1    : ctaaatataaaccgactatcggcgcgacagttaacctgaattctgcaggtaaattgaccactgataccaccagtgcaggcacagcaaccaaagatcctct : 1364
H7_AY337468.1     : ---attatgctccagcaattggtactgctgtaaatgtgaactccgcgggtaaaatcactactgagactaccagtgctggttctgcaacgaccaacccgct : 1355
H6_AY249991.1     : ---ctgaggatgcggctgccggtaaactggtctacaaaggttccgatggtaagttaacaacggatacgactagcaaagcagaatcaacatcagatccgct : 1244
H18_AY250001.1    : ---gtgaaaatgcggctaccagtaaagctatctatgtaagtgccaatggtaacttaacgactaatacaactagtgaatctgaagctactaccaacccgct : 1262
H34_AY250016.1    : ---gcg------------caggcaaagaaatctataaagacgctgatggtgtctacagcacaga------gaataaaacatcgaagacgtccgatccatt : 1235
H14_AY249998.1    : ---gca------------gcggtaaggcagtttataaagatgctgacggtaaattgacgacagatgctgaaactaaagctgcaaccaccgccgatcccct : 1250
```

```
H15_AY249999.1    : ---gtt------------caggtaaagcggtctatgtagaagcggatggtgatttcactaccgacgctgcaaccaagccgcaaccaccaccgatccgct : 1286
H20_AY250003.1    : ---gtt------------caggtaaagcggtctatgtagaagcggatggtgatttcactaccgacgctgcaaccaagccgcaaccaccaccgatccgct : 1328
H31_AY250013.1    : ---gtt------------ctggtaaagctgtgtatgctgatgcaacaggaaaactaactactgacgctgaaactaaagccgaaaccaccgccgatccccct : 1265
H28_AY250010.1    : ---gtt------------caggtaaagcagtctatgtttcagcggatggtaattttactactgacgctgaaactaaagctgcaaccaccgccgatccact : 1337
H46_AY250024.1    : ---gtt------------ctggtaaaggggtttacaccgatgcagctggtaaattcactaccgacgctgcaaccaaagccgcaaccaccaccgatccgct : 1316
H44_AB269770.1    : ---att------------ccggtaaggctgtatatgtacaggaagatggaaattcaccacagatgcagcaactaaagcagcaaccactgctgacccact : 1322
H19_AY250002.1    : ---ata------------caggctccactatttatgcgacagaagatggtagcctgaccaccgatgcagcaaccaaagccgaaaccaccgccgatccccct : 1439
H55_AB269771.1    : ---aca------------gtggttcagctatttacgcaactgaagcgggcaaattgactactgaagcgtctacagctgctgaaactaccgctaacccact : 1466
H26_AY250008.1    : ---gtg------------aaggttctactgtctataaagctgctgatggttccatcactaaagatgcgactaccaagtctgaagcaaccactgaccctct : 1271
H30_AY250011.1    : ---atg------------acggcgcggctctgtacaaagcggctgacggttctctgaccactgaagctactggtaaatccgaagtgaccacggatccgct : 1310
H32_AY250014.1    : ---atg------------acggcgcggctctgtataaagcggctgacggctctctgaccactgaagctacaggtaaatctgaagcgaccacggatccgct : 1310
H37_AY250017.1    : ---ata------------aaggcgctgctgtttatgcggcggcggatggttctctgactacagaaactacaagtaaatcagaagctacagctaacccgct : 1283
H41_AY250020.1    : ---aca------------aaggctcgtcactgtataaagctgcagatggctctctgacttctgaagctaaagggaaatctgaagcaaccgccgatccccct : 1277
H9_AY249994.1     : ---gca------------ccggtggtaccatctatgaaacagctgatggtaagttaacaacagaagctactactgcatccagttccaccgccgatccccct : 1610
H36_EF392693.1    : ---gtt------------ctggtaaggttgcttataaagacgcagatggtaaaattactactgatgctaaaactgaaacagcaaagacaaccgatcctat : 1271
                                                                      c                  ac      a cc  cT

                          *      2020         *      2040         *      2060         *      2080         *      2100
H21_AIHL01000060.1: cgaaactatcgacaaagcattggctaaagttgacaatctgcgttctgacctcggtgcagtacaaaaccgtttcgactctgccatcaccaaccttggcaac : 1176
H8_AJ865465.1     : cgaaactatcgacaaggcattggctaaagttgacaatctgcgttctgacctcggtgcagtacaaaaccgtttcgactctgccatcaccaaccttggcaac : 1179
H40_AJ884568.1    : cgaaactatcgacaaagcattggctaaagttgacaatctgcgttctgacctcggtgcagtacaaaaccgtttcgactctgctatcaccaaccttggcaac : 1179
H11_AY337465.1    : cgaaactatcgacaaagcattggctaaagttgacaatctgcgttctgacctcggtgcagtacaaaaccgtttcgactctgctatcaccaaccttggcaac : 1164
H27_AM231154.2    : cgaaaccatcgacaaggcattggctaaagttgacaatctgcgttctgacctcggtgcagtacaaaaccgtttcgactctgctatcaccaaccttggcaac : 1161
H2_AIHA01000023.1 : ggctgctatcgataaagcactgtctcaggttgactcgttgcgttctaacctaggtgcaattcaaaatcgtttcgactctgccatcaccaaccttggcaac : 1194
H16_AY337475.1    : atccaaattagacgaagctttggcaaaagttgataagttgcgcagttctttgggtgcagtacaaaaccgtttcgactctgccatcaccaaccttggcaac : 1275
H3_AB128916.1     : ggaaaaattagatacagctttagctaaagttgataagttgcgcagctcccttggtgcggtacagaaccgttttgattctgctattaccaacttgggtaat : 1290
H54_AB128918.1    : ggatgcaattgataaagctttagcgcaggttgactccttgcgttcagacttgggggcggtgcagaaccgtttcgactccacaatcaccaacttgggaaat : 1251
H53_AB128917.1    : agcaactatcgataatgctttgaagattgttgactctcaacgtagctctttaggtgccatccagaaccgtttcgattcggccatcactaacttgggtaac :  972
H47_EF392694.1    : atcaaaaattgatgatgcgatctctgatgttgattcacttcgctctgatttaggtgctgtacaaaaccgcttcgattctgctattaccaacttaggtaac :  807
H35_EF392692.1    : taaaagtattgatgatgcactttcccaagtggatcaattccgtagctccttgggggcagtgcaaaaccgcttcgattctgctatcactaacttaggtaat : 1209
H43_AIGA01000038.1: ggctgccctggatgatgcaattgcgatggtcgacaaattccgctcctccctcggtgcggtgcaaaaccgtctggattccgcagtcaccaacctgaacaac : 1203
H17_CP002291.1    : ggcggctctggatgcagctatcgcttctgtcgataaattccgttcttctctgggtgcggtacagaaccagttgaattctgctatcaccaacctgaacaac : 1221
H5_AY249990.1     : ggcactttttagacaaagctattgcacaggttgatacttttccgctcctccctcggtgcggtgcaaaaccgtctggattccgcagtaaccaacctgaacaac : 1008
H56_AY250029.1    : ggcactttttagacaaagccattgcacaggttgatacttttccgctcctccctcggtgccgttcaaaaccgtctggactctgcggtaaccaacctgaacaac : 1008
H42_AY250021.1    : ggcactttttagacaaagctattgcacaggttgatacttttccgctcctccctcggtgccgttcaaaaccgtctggattccgcagtcaccaacctgaacaac :  978
H29_AY250012.1    : gactctgctggacaaagctatcgcatctgttgataaattccgttcttctttgggggcggtacagaaccgtctgagctccgctgtaaccaacctgaacaac : 1029
H38_AY250018.1    : gactctgctggacaaagctatcgcatctgttgataaattccgttcttctttgggggcggtacagaaccgtctgagctccgctgtaaccaacctgaacaac : 1041
H33_AY250015.1    : ggctctgctggacaaagctatcgcatctgttgataaattccgttcttctctagggggcggtgcagaaccgtctgagctctgctgtaaccaacctgaacaac :  984
H39_AY250019.1    : ggcactattggacaaagctatcgcttctgttgataaattccgttcttctctgggtgcggtacagaatcgtctgagctctgctgtaaccaacctgaataac :  996
H52_AY250028.1    : ggcactttttagacaaagctatctcgcaagttgatacttttccgctcctccctcggtgccgtacaaaaccgtctggattctgcggtcaccaacctgaataac : 1041
H4_AJ536600.1     : aaaagcactggatgccgcaatcgctaaagtcgacaaattccgctcctccctcggtgccgttcaaaaccgtctggattctgcggtcaccaacctgaacaac :  747
H25_AGSG01000116.1: aaaagcactggatgccgcaatcgctaaagtcgacaaattccgctcctccctcggtgccgttcaaaaccgtctggattctgcggtcaccaacctgaacaac :  747
H10_AY249995.1    : taaggctcttgatgatgcaattgcatctgttgataaattccgctcttctctcggtgccgttcagaaccgtctggattctgccattgccaacctgaacaac :  960
H24_AY250006.1    : ggctgcccttgacgacgcaatcagccagatcgacaaattccgctcctccctcggtgcggtgcagaaccgtctggattccgcggtcaccaacctgaacaac : 1179
H48_AY250025.1    : gaaagcgctggacgatgctatcgcatctgtagacaaattccgttcttccctcggtgcggtgcaaaaccgtctggattccgcggttaccaacctgaacaac : 1194
H1_AB028471.1     : gaaagctctggatgaagctatcagctccatcgacaaattccgttcatccctgggggctatccaaaaccgtttggattccgccgtcaccaacctgaacaac : 1485
```

```
H12_AY337471.1    : gaaagctctggatgaagctatcagctccatcgacaaattccgttcttccctgggtgctatccagaaccgtctggattccgcagtcaccaacctgaacaac : 1485
H51_AY250027.1    : ggctgccctggatgctgctatcagctccatcgacaaattccgttcctccctgggtgctatccagaaccgtctggattccgcagtcaccaacctgaacaac : 1515
H45_AY250023.1    : ggctgccctggatgaagctatcagctccatcgacaaattccgttcatccctgggtgctatccagaaccgtctggattccgcggttaccaacctgaacaac : 1404
H49_AY250026.1    : aaatgcgctggatgaggcaattgcatccatcgacaaattccgttcttccctgggggctatccagaaccgtctggattccgcagtcaccaacctgaacaac : 1392
H23_AB028476.1    : ggctgccctggacgctgctatcagctccatcgacaaattccgttcatccctgggtgctatccagaaccgtctggattccgcagtcaccaacctgaacaac : 1464
H7_AY337468.1     : tgctgccctggacgacgcaatcagctccatcgacaaattccgttcttccctgggtgctatccagaaccgtctggattccgcagtcaccaacctgaacaac : 1455
H6_AY249991.1     : ggcagctcttgacgacgctatcagccagatcgacaaattccgctcctccctgggtgcggtgcaaaaccgtctggattccgcagtgaccaacctgaacaac : 1344
H18_AY250001.1    : ggcagcattggatgacgctatcgcgtctatcgacaaattccgttcttccctgggtgctatccagaaccgtctggattccgcagtcaccaacctgaacaac : 1362
H34_AY250016.1    : ggctgcgcttgacgacgcaatcagctccatcgacaaattccgttcatccttgggtgctatccagaaccgtctggattccgcggtcaccaacctgaacaac : 1335
H14_AY249998.1    : gaaagctctggacgaagccatcagctccatcgacaaattccgctcctccctcggtgcggtgcagaaccgtctggattccgcggtcaccaacctgaacaac : 1350
H15_AY249999.1    : gaaagccctggatgaggcaatcagccagatcgataagttccgttcatccctgggtgctatccagaaccgtctggattccgcggtcaccaacctgaacaac : 1386
H20_AY250003.1    : ggccgctctggatgacgcaatcagccagatcgacaagttccgttcatccttgggtgctatccagaaccgtctggattctgcagtcaccaacctgaacaac : 1428
H31_AY250013.1    : gaaagctctggacgaagcgatcagctccatcgacaaattccgttcttccctcggtgcggtgcaaaaccgtctggattccgcggtcaccaacctgaacaac : 1365
H28_AY250010.1    : gaaagctctggacgaagcgatcagctccatcgacaaattccgttcttccctcggtgcggtgcaaaaccgtctggattccgcagtcaccaacctgaacaac : 1437
H46_AY250024.1    : gaaagcccttgatgacgcaatcagccagatcgataagttccgttcatccctgggtgctatccagaaccgtctggattccgcggttaccaacctgaacaac : 1416
H44_AB269770.1    : gaaagcgctggacgatgcaatcagctctatcgacaaattccgttcttccctgggtgctgtacagaaccgtctggattctgcagtaaccaacctgaacaac : 1422
H19_AY250002.1    : gaaagctctggacgaagccatcagctccatcgacaaattccgctcctccctcggtgcggtgcaaaaccgtctggattccgcggtcaccaacctgaacaac : 1539
H55_AB269771.1    : gaaagccctggacgatgcaatcagccagatcgacaaattccgttcttctctgggtgctgtacagaaccgtctggattctgcggtaaccaacctgaacaac : 1566
H26_AY250008.1    : gaaagcccttgacgacgcaatcagccagatcgacaaattccgctcctccctcggtgccgttcaaaaccgtctggattccgccgtcaccaacctgaacaac : 1371
H30_AY250011.1    : gaaagcgctggacgatgctatcgcatccgtagacaaattccgctcctccctcggtgcggtgcagaaccgtctggattccgcagtcaccaacctgaacaac : 1410
H32_AY250014.1    : gaaagcgctggacgatgctatcgcatccgtagacaaattccgttcttccctgggtgccgtgcagaaccgtctggattccgcagtcaccaacctgaacaac : 1410
H37_AY250017.1    : ggccgctctggacgacgcaatcagccagatcgacaaattccgttcatccctgggtgctatccagaaccgtctggattccgcagtcaccaacctgaacaac : 1383
H41_AY250020.1    : gaaagctctggacgaagccatcagctccatcgacaaattccgctcctccctcggtgccgttcaaaaccgtctggattctgcggtgaccaacctgaacaac : 1377
H9_AY249994.1     : gaaagctctggacgaagccatcagctccatcgacaaattccgctcctccctcggtgcggtgcaaaaccgtctggattccgcggtcaccaacctgaacaac : 1710
H36_EF392693.1    : ggcaaagttggataaagctctagctaaagttgacgcattacgttctgacttgggtgcaatccagaaccgtttcgattctaccatcaccaacttgggcaac : 1371
                                T GA  a GC   T        T GA aa tt CG tc tcccT GGtGC   T CA AAcCgt T ga TC gC  T aCcAACcTg  cAAc

                         *      2120         *      2140         *      2160
H21_AIHL01000060.1: accgtaaacaacctgtcttctgcccgtagccgtatcgaagatgctgactacgcgaccgaagtgtctaa : 1244
H8_AJ865465.1     : accgtaaacaacctgtcttctgcccgtagccgtatcgaagatgctgactacgcgaccgaagtgtctaa : 1247
H40_AJ884568.1    : accgtaaacaacctgtcttctgcccgtagccgtatcgaagatgctgactacgcgaccgaagtgtctaa : 1247
H11_AY337465.1    : accgtaaacaacctgtcttctgcccgtagccgtatcgaagatgctgactacgcgaccgaagtgtctaa : 1232
H27_AM231154.2    : accgtaaacaacctgtcttctgcccgtagccgtatcgaagatgctgactacgcgaccgaagtgtctaa : 1229
H2_AIHA01000023.1 : accgtaaacaacctgtcttctgcccgtagccgtatcgaagatgctgactacgcgaccgaagtgtctaa : 1262
H16_AY337475.1    : accgtaaacaacctgtcttctgcccgtagccgtatagaagatgctgactacgcaaccgaagtgtctaa : 1343
H3_AB128916.1     : actgttaacaacctgtcttctgcacgtagccgtatcgaagattccgactacgcgactgaagtatctaa : 1358
H54_AB128918.1    : actctaaacaacttatcttctgctcgaagccgtattgaagatgctgactacgcgactgaagtctctaa : 1319
H53_AB128917.1    : acagtgaacaacctgtcttctgcacgtagccgtatcgaagactccgactacgcgaccgaagtgtctaa : 1040
H47_EF392694.1    : actgtaaataatctgtcttcagcacgtagccgtattgaagactctgactacgcaaccgaagtatctaa :  875
H35_EF392692.1    : actgtaaacaatctgtcttctgcacgtagccgtatcgaagattccgactacgcgaccgaagtatctaa : 1277
H43_AIGA01000038.1: accactaccaacctgtctgaagcgcagtcccgtattcaggacgccgactatgcgaccgaagtgtccaa : 1271
H17_CP002291.1    : accaccaccaacctgtctgaagcgcagtcccgtattcaggacgccgactatgcgaccgaagtgtcaaa : 1289
H5_AY249990.1     : accaccaccaacctgtctgaagcgcagtcccgtattcaggacgccgactatgctacagaagtgtccaa : 1076
H56_AY250029.1    : accaccaccaacctgtctgaagcgcagtcccgtattcaggacgccgactatgcgaccgaagtgtctaa : 1076
H42_AY250021.1    : actactaccaacctgtctgaagcgcagtcccgtattcaggacgccgactatgcgaccgaagtgtccaa : 1046
H29_AY250012.1    : accactaccaacctgtctgaagcgcagtcccgtattcaggacgccgactatgcgaccgaagtgtccaa : 1097
```

```
H38_AY250018.1     : accaccaccaacctgtctgaagcgcagtcccgtattcaggacgccgactatgcgaccgaagtgtccaa : 1109
H33_AY250015.1     : accactaccaacctgtccgaagcgcagtcccgtattcaggacgccgactatgcgaccgaagtgtccaa : 1052
H39_AY250019.1     : accaccactaacctgtctgaagcgcagtcccgtattcaggatgccgactatgcgaccgaagtgtcaaaa : 1064
H52_AY250028.1     : accaccaccaacctgtctgaagcgcagtcccgtattcaggacgccgactatgcgaccgaagtgtccaa : 1109
H4_AJ536600.1      : accaccaccaacctgtctgaagcgcagtcccgtattcaggacgccgactatgcgaccgaagtgtccaa :  815
H25_AGSG01000116.1: accaccaccaacctgtctgaagcgcagtcccgtattcaggacgccgactatgcgaccgaagtgtccaa :  815
H10_AY249995.1     : accactaccaacctgtctgaagcgcagtcccgtattcaggacgctgactatgcgaccgaagtgtccaa : 1028
H24_AY250006.1     : accactaccaacctgtctgaagcgcagtcccgtattcaggacgctgactatgcgaccgaagtatccaa : 1247
H48_AY250025.1     : accactaccaacctgtctgaagcgcagtcccgtattcaggacgccgactatgcgaccgaagtgtccaa : 1262
H1_AB028471.1      : accactaccaacctgtctgaagcgcagtcccgtattcaggacgccgactatgcgaccgaagtgtccaa : 1553
H12_AY337471.1     : accactaccaacctgtccgaagcgcagtcccgtattcaggacgccgactatgcgaccgaagtgtccaa : 1553
H51_AY250027.1     : accactactaacctgtctgaagcgcagtcccgtattcaggacgccgactatgcgaccgaagtgtccaa : 1583
H45_AY250023.1     : accactaccaacctgtccgaagcgcagtcccgtattcaggacgccgactatgcgaccgaagtgtccaa : 1472
H49_AY250026.1     : accactaccaacctgtctgaagcgcagtcccgtattcaggacgccgactatgcgaccgaagtgtccaa : 1460
H23_AB028476.1     : accactaccaacctgtccgaagcgcagtcccgtattcaggacgccgactatgcgaccgaagtgtccaa : 1532
H7_AY337468.1      : accactaccaacctgtccgaagcgcagtcccgtattcaggacgccgactatgcgaccgaagtgtccaa : 1523
H6_AY249991.1      : accactaccaacctgtctgaagcgcagtcccgtattcaggacgccgactatgcgaccgaagtgtccaa : 1412
H18_AY250001.1     : accactaccaacctgtctgaagcgcagtcccgtattcaggacgccgactatgcgaccgaagtgtccaa : 1430
H34_AY250016.1     : accactaccaacctgtccgaagcgcagtcccgtattcaggacgccgactatgcgaccgaagtgtccaa : 1403
H14_AY249998.1     : accactaccaacctgtctgaagcgcagtcccgtattcaggacgctgactatgcgaccgaagtatccaa : 1418
H15_AY249999.1     : accactaccaacctgtctgaagcgcagtcccgtattcaggacgccgactatgcgaccgaagtgtccaa : 1454
H20_AY250003.1     : accaccaccaacctgtctgaagcgcagtcccgtattcaggacgccgactatgcgaccgaagtgtccaa : 1496
H31_AY250013.1     : accactaccaacctgtccgaagcgcagtcccgtattcaggacgccgactatgcgaccgaagtgtccaa : 1433
H28_AY250010.1     : accactactaacctgtctgaagcgcagtcccgtattcaggacgctgactatgcgaccgaagtgtccaa : 1505
H46_AY250024.1     : accactaccaacctgtccgaagcgcagtcccgtattcaggacgccgactatgcgaccgaagtgtccaa : 1484
H44_AB269770.1     : accaccaccaacctgtctgaagcgcagtcccgtattcaggacgccgactatgcgaccgaagtgtcaaaa : 1490
H19_AY250002.1     : accaccaccaacctgtctgaagcgcagtcccgtattcaggacgccgactatgcgaccgaagtgtccaa : 1607
H55_AB269771.1     : accaccaccaacctgtctgaagcgcagtcccgtattcaggacgccgactatgcgaccgaagtgtcaaaa : 1634
H26_AY250008.1     : accactaccaacctgtctgaagcgcagtcccgtattcaggacgccgactatgcgaccgaagtgtccaa : 1439
H30_AY250011.1     : accactaccaacctgtctgaagcgcagtcccgcattcaggacgccgactatgcgaccgaagtgtccaa : 1478
H32_AY250014.1     : accactaccaacctgtccgaagcgcagtcccgtattcaggacgccgactatgcgaccgaagtgtccaa : 1478
H37_AY250017.1     : accactaccaatctgtctgaagcgcagtcccgtattcaggacgccgactatgcgaccgaagtgtccaa : 1451
H41_AY250020.1     : accactaccaacctgtctgaagcgcagtcccgtattcaggacgccgactatgcgaccgaagtgtccaa : 1445
H9_AY249994.1      : accactaccaacctgtccgaagcgcagtcccgtattcaggacgccgactatgcgaccgaagtgtccaa : 1778
H36_EF392693.1     : actgttaacaacttgacttccgcacgtagtcgtatcgaagatgctgactatgcgacagaagtgtctaa : 1439
                     ACc   A  cAAccTgtCt    GC C    cCGtATt A GA gC GACTA GCgACcGAAGTgTC AA
```

Supplementary materials Figure 1. Comparative analysis of sequences of *fliC* genes and its homologs (in total 53 sequences) coding for 53 different serotypes of H-antigen (*E. coli* flagellin). The reference sequences were previously selected and deposited by Joensen et al., 2015 [17]. The sequences of the universal primers: FLIC1: 5′-GGT-CAG-GCG-ATT-GCT-AAC-CG-3′ and FLIC2: 5′-TTG-GAC-ACT-TCG-GTC-GCA-TAG-TC-3′ are marked with yellow.

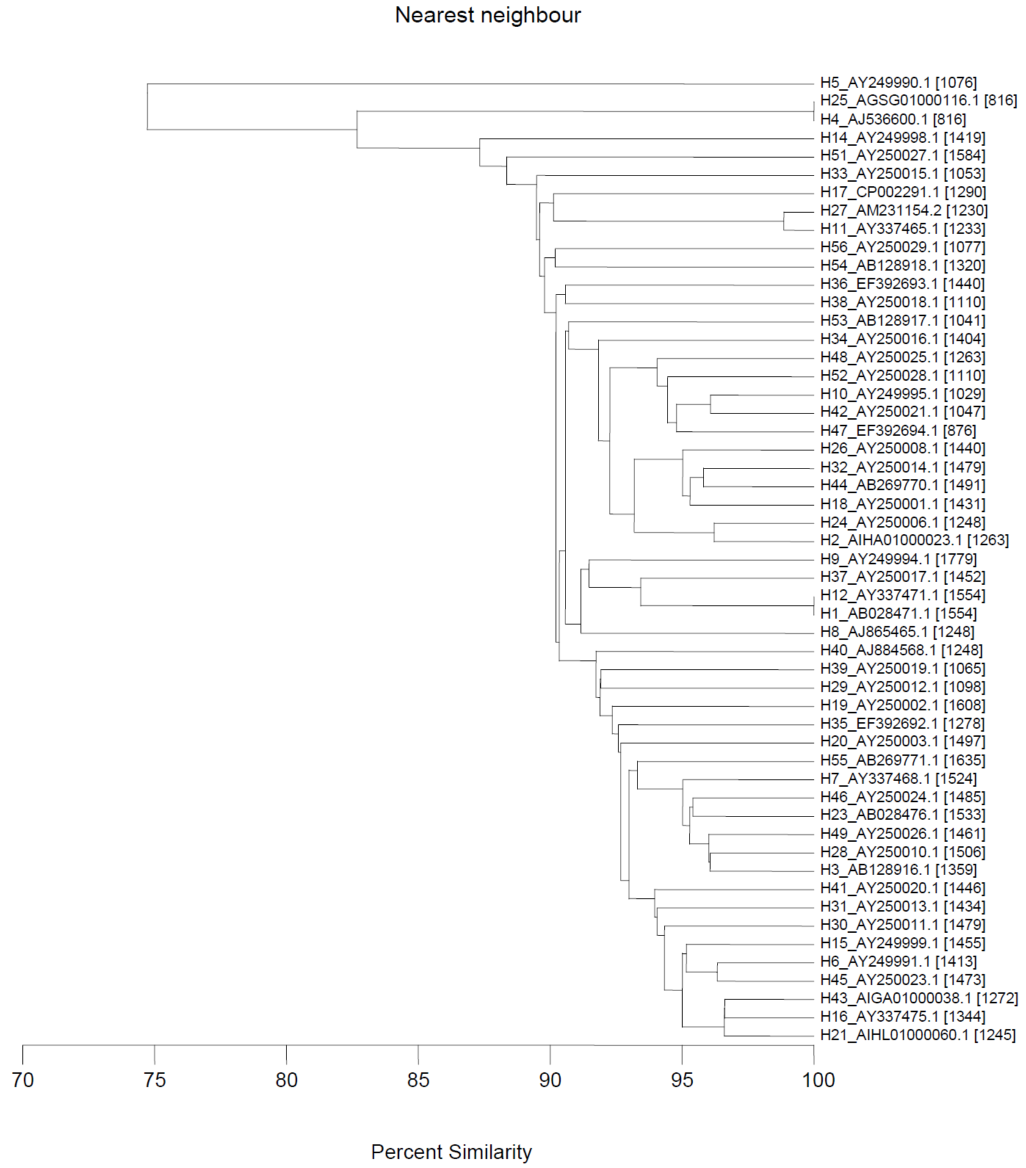

Supplementary materials Figure 2. Differentiation of sequences of *fliC* genes and its homologs (in total 53 sequences) coding for 53 different serotypes of H-antigen (*E. coli* flagellin) with proposed RFLP-PCR *Rsa*I method. The reference sequences were previously selected and deposited by Joensen et al., 2015 [17].

```
                     *        20         *        40         *        60         *        80         *       100
H4_AJ53660 : ggccaggcgattgctaaccgctttacttctaacatcaaaggtctgactcaggccgcacgtaacgccaacgacggtatttctctggcgcagacggctgaag : 100
H25_AGSG01 : ggccaggcgattgctaaccgcttcacttctaacatcaaaggtctgactcaggccgcacgtaacgccaacgacggtatttctctggcgcagacggctgaag : 100
H1_AB02847 : ggtcaggcgattgctaaccgtttttacttctaacattaaaggcctgactcaggctgcacgtaacgccaacgacggtatttctgttgcacagaccactgaag : 100
H12_AY3374 : ggtcaggcgattgctaaccgtttttacttctaacattaaaggcctgactcaggctgcacgtaacgccaacgacggtatttccgttgcgcagaccaccgaag : 100
             GG CAGGCGATTGCTAACCG TTtACTTCTAACAT AAAGG CTGACTCAGGC GCACGTAACGCCAACGACGGTATTTCt T GCgCAGAC  CtGAAG

                     *       120         *       140         *       160         *       180         *       200
H4_AJ53660 : gcgcgctgtcagagattaacaacaacttgcagcgtattcgtgaactgaccgttcaggcctctaccggcacgaactctgattccgacctgtcttctattca : 200
H25_AGSG01 : gcgcgctgtcagagattaacaacaacttgcagcgtattcgtgaactgaccgttcaggcctctaccggcacgaactctgattccgacctgtcttctattca : 200
H1_AB02847 : gcgcgctgtccgaaatcaacaacaacttacagcgtatccgtgagctgacggttcaggcttctaccgggactaactctgattcggatctggactccattca : 200
H12_AY3374 : gcgcgctgtccgaaatcaacaacaacttacagcgtatccgtgaactgacggttcaggcttctaccgggactaactccgattcggatctggactccattca : 200
             GCGCGCTGTC GA AT AACAACAACTT CAGCGTAT CGTGAaCTGAC GTTCAGGC TCTACCGG AC AACTCtGATTC GA CTG   TC ATTCA

                     *       220         *       240         *       260         *       280         *       300
H4_AJ53660 : ggacgaaatcaaatcccgtcttgatgaaattgaccgtgtatctggtcagacccagttcaacggtgtgaacgtgctgtcgaaaaacgattcgatgaagatt : 300
H25_AGSG01 : ggacgaaatcaaatcccgtcttgatgaaattgaccgtgtatctggtcagacccagttcaacggtgtgaacgtgctgtcgaaaaacgattcgatgaagatt : 300
H1_AB02847 : ggacgaaatcaaatcccgtctcgacgaaattgaccgcgtatccggtcagacccagttcaacggcgtgaacgtactggcaaaagacggttcgatgaaaatt : 300
H12_AY3374 : ggacgaaatcaaatcccgtctggacgaaattgaccgcgtatctggccagacccagttcaacggcgtgaacgtactggcgaaagacggttcaatgaaaatt : 300
             GGACGAAATCAAATCCCGTCT GA GAAATTGACCG GTATCtGGtCAGACCCAGTTCAACGG GTGAACGT CTG CgAAA ACG TTCgATGAA ATT

                     *       320         *       340         *       360         *       380         *       400
H4_AJ53660 : cagattggtgccaatgataaccagacgatcagcattggcttgcaacaaatcgacagtaccacttttgaatctgaaaggatttaccgtgtccggcatggcg- : 399
H25_AGSG01 : cagattggtgccaatgataaccagacgatcagcattggcttgcaacaaatcgacagtaccacttttgaatctgaaaggatttaccgtgtccggcatggcg- : 399
H1_AB02847 : caggttggtgcgaacgacggccagactatcactattgatctgaagaaaattgactctgatacgctggggctgaatggttttaacgtgaatggttccggta : 400
H12_AY3374 : caggttggtgcgaatgacggccagactatcacgattgatctgaagaaaattgactcagatacgctggggctgaatggttttaacgtgaatggttccggta : 400
             CAG TTGGTGC AAtGA   CCAGAC ATCA  ATTG   TG A  AAAT GAC  t   AC   TG   CTGAA GG TTTA CGTG   GG   G

                     *       420         *       440         *       460         *       480         *       500
H4_AJ53660 : --gatttcagcgcggcgaaactgacggctgctga---tggtacagcaa---------------------------------------------------- : 442
H25_AGSG01 : --gatttcagcgcggcgaaactgacggctgctga---tggtacagcaa---------------------------------------------------- : 442
H1_AB02847 : cgatagccaataaagcggcgaccattagcgacct---gacagcagcgaaaatggatgctgca-------------------------------------- : 459
H12_AY3374 : cgatagccaataaagcggcgaccattagcgacct---gacagcagcgaaaatggatgctgca-------------------------------------- : 459
                    CA     GCG      A     G            CAGC A

                     *       520         *       540         *       560         *       580         *       600
H4_AJ53660 : -----------------------------------------------------ttgctgctgcggatgtcaa---ggatgctgggggtaaacaagtc--- : 483
H25_AGSG01 : -----------------------------------------------------ttgctgctgcggatgtcaa---ggatgctgggggtaaacaagtc--- : 483
H1_AB02847 : -------------actaatactata------actacaacaaataatgcgc---tgactgcatcaaaggccct------tgatcaactgaaagatggt--- : 528
H12_AY3374 : -------------actaatactata------actacaacaaataatgcgc---tgactgcatcaaaggcgct------tgatcaactgaaagatggt--- : 528
                                                                  T  CTGC  C  A G c        TG T      AAA A G

                     *       620         *       640         *       660         *       680         *       700
H4_AJ53660 : aatttac----------tgtctt----------------------------------------------------------------------------- : 496
H25_AGSG01 : aatttac----------tgtctt----------------------------------------------------------------------------- : 496
H1_AB02847 : gacac------------tgttac---tatcaaagcagatgcagctcaaactg------------------------------ccacggtctatacataca : 583
```

```
H12_AY3374 : gacac------------tgttac---tatcaaagcagatgctgctcaaactg------------------------------ccacggtttatacataca :  583
              A                TGT

                    *      720        *      740        *      760        *      780        *      800
H4_AJ53660 : ---------------------------------------------------------------------------------------------------- :    -
H25_AGSG01 : ---------------------------------------------------------------------------------------------------- :    -
H1_AB02847 : atgcatctgctggtaacttctc---------------------------attcagtaatgtatcgaataatacttcagcaaaag------------cagg :  644
H12_AY3374 : atgcatcagctggtaacttctc---------------------------attcagtaatgtatcgaataatacttcagaaaaag------------cagg :  644

                    *      820        *      840        *      860        *      880        *      900
H4_AJ53660 : -----------------------------------------------------------------------------------acactgacaccgcgtct :  513
H25_AGSG01 : -----------------------------------------------------------------------------------acactgacaccgcgtct :  513
H1_AB02847 : tgatgtagcagctagccttctcccgccggctgggcaa---actgctagtggtgtttacaaagcagcaagcg------------gtgaagtgaactttgat :  729
H12_AY3374 : tgatgtagcagctagccttctcccgccggctgggcaa---actgctagtggtgtttataaagcagcaagcg------------gtgaagtgaactttgat :  729
                                                                                                  G  A C     T

                    *      920        *      940        *      960        *      980        *     1000
H4_AJ53660 : aacagtactaaatatgcgg--------------------------------------------------------------------------------- :  532
H25_AGSG01 : aacagtactaaatatgcgg--------------------------------------------------------------------------------- :  532
H1_AB02847 : gttgatgcgaatggtaaaa---ttac-------------aatcggaggacaggaagcctatttaactagtgatggtaacttaactacaaacgatgctggt :  813
H12_AY3374 : gttgatgcgaatggtaaaa---tcac-------------aatcggaggacagaaagcatatttaactagtgatggtaacttaactacaaacgatgctggt :  813
                   T C AA   T

                    *     1020        *     1040        *     1060        *     1080        *     1100
H4_AJ53660 : ---------------------------------------------------------------------------------------------------- :    -
H25_AGSG01 : ---------------------------------------------------------------------------------------------------- :    -
H1_AB02847 : ---------------ggtgcg-----actgcggctacgcttgatggtttattcaagaaagctggtgatggtca---------------------atcaat :  872
H12_AY3374 : ---------------ggtgcg-----actgcggctacgcttgatggtttattcaagaaagctggtgatggtca---------------------atcaat :  872

                    *     1120        *     1140        *     1160        *     1180        *     1200
H4_AJ53660 : -----------------tcgttgattctgcaaccggta-------------------------------------------------------------- :  553
H25_AGSG01 : -----------------tcgttgattctgcaaccggta-------------------------------------------------------------- :  553
H1_AB02847 : cgggtttaataagactgcatcagtcacgatggggggaa-------------------------------------------------------------- :  910
H12_AY3374 : cgggtttaagaagactgcatcagtcacgatggggggaa-------------------------------------------------------------- :  910
                                 G   C       GG A

                    *     1220        *     1240        *     1260        *     1280        *     1300
H4_AJ53660 : ----------------------------------------------------aatacatggaagccactgtagccattaccggtacggcggcggcg---- :  597
H25_AGSG01 : ----------------------------------------------------aatacatggcagccactgtagtcattaccagtacggcggcggcg---- :  597
H1_AB02847 : ----------------------------------------------------caacttataactttaaaacgggtgctgatgctgg-------------- :  944
H12_AY3374 : ----------------------------------------------------caacttataactttaaaacgggtgctgatgctga-------------- :  944
                                                                     A       a    A     G    T    g T

                    *     1320        *     1340        *     1360        *     1380        *     1400
```

```
H4_AJ53660 : ---------------------------------------------------------------------------------------------------- :    -
H25_AGSG01 : ---------------------------------------------------------------------------------------------------- :    -
H1_AB02847 : ---------------------------------------------------------------------------------------------------- :    -
H12_AY3374 : ---------------------------------------------------------------------------------------------------- :    -

                   *     1420         *     1440         *     1460         *     1480         *     1500
H4_AJ53660 : ---------------------------------------------------------------------------------------------------- :    -
H25_AGSG01 : ---------------------------------------------------------------------------------------------------- :    -
H1_AB02847 : ---------------------------------------------------------------------------------tgctgcaactgctaacgca :  963
H12_AY3374 : ---------------------------------------------------------------------------------tgctgcaactgctaacgca :  963

                   *     1520         *     1540         *     1560         *     1580         *     1600
H4_AJ53660 : ---------------------------------------------------------------------------------------------------- :    -
H25_AGSG01 : ---------------------------------------------------------------------------------------------------- :    -
H1_AB02847 : ggggtatcgttcactgatacagctagcaaagaaaccgttttaaataaagtggctacagctaaacaaggcacagcagttgcagctaacggtgatacatccg : 1063
H12_AY3374 : ggggtatcgttcactgatacagctagcaaagaaaccgttttaaataaagtggctacagctaaacaaggcaaagcagctgcagctgacggtgatacatccg : 1063

                   *     1620         *     1640         *     1660         *     1680         *     1700
H4_AJ53660 : ---------------------------------------------------------------------------------------------------- :    -
H25_AGSG01 : ---------------------------------------------------------------------------------------------------- :    -
H1_AB02847 : ------caacaattacctataaatctggcgttcagacgtatcaggcggtatttgccgcaggtgacggtactgctagcgcaaaatatgccgata------a : 1151
H12_AY3374 : ------caacaattacctataaatctggcgttcagacgtatcaggcggtatttgccgcaggtgacggtactgctagcgcaaaatatgccgata------a : 1151

                   *     1720         *     1740         *     1760         *     1780         *     1800
H4_AJ53660 : ---------------------------------------------------------------------------------------------------- :    -
H25_AGSG01 : ---------------------------------------------------------------------------------------------------- :    -
H1_AB02847 : tactgacgtttctaatgcaacag---------caacatacacagatgctgatggtga------------------------aatgactacaattggttca : 1218
H12_AY3374 : agctgacgtttctaatgcaacag---------caacatacactgatgctgatggtga------------------------aatgactacaattggttca : 1218

                   *     1820         *     1840         *     1860         *     1880         *     1900
H4_AJ53660 : ---------------------------------------------------------------------------------------------------- :    -
H25_AGSG01 : ---------------------------------------------------------------------------------------------------- :    -
H1_AB02847 : tacaccac------gaagtattcaatcgatgctaacaacggcaaggtaactgttgattct---------ggaactggtacgggta--------------- : 1288
H12_AY3374 : tacaccac------gaagtattcaatcgatgctaacaacggcaaggtaactgttgattct---------ggaactggtacgggta--------------- : 1288

                   *     1920         *     1940         *     1960         *     1980         *     2000
H4_AJ53660 : --------------------------------------------------gtaactgttggtgcagcggaagtggcgggagccgctacagccgatccgtt :  647
H25_AGSG01 : --------------------------------------------------gtaactgttggtgcaacggaagtggcgggagccgctacagccgaaccgtt :  647
H1_AB02847 : ---aatatgcgccgaaagtcggggctgaagtatatgttagtgctaatggtactttaacaacagatgcaactagcgaaggcacagtaacaaaagatccact : 1385
H12_AY3374 : ---aatatgcgccgaaagtaggggctgaagtatatgttagtgctaatggtactttaacaacagatgcaactagcgaaggcacagtaacaaaagatccact : 1385
```

```
                                                                         G  gC        G  GG  C  G  ACA    GAtCC  T

                 *     2020         *     2040         *     2060         *     2080         *     2100
H4_AJ53660 : aaaagcactggatgccgcaatcgctaaagtcgacaaattccgctcctccctcggtgccgttcaaaaccgtctggattctgcggtcaccaacctgaacaac :  747
H25_AGSG01 : aaaagcactggatgccgcaatcgctaaagtcgacaaattccgctcctccctcggtgccgttcaaaaccgtctggattctgcggtcaccaacctgaacaac :  747
H1_AB02847 : gaaagctctggatgaagctatcagctccatcgacaaattccgttcatccctggggctatccaaaaccgttggattccgccgtcaccaacctgaacaac : 1485
H12_AY3374 : gaaagctctggatgaagctatcagctccatcgacaaattccgttctttccctgggtgctatccagaaccgtctggattccgcagtcaccaacctgaacaac : 1485
              AAAGC CTGGATG  GC ATC       TCGACAAATTCCG TC TCCCT GGtGC  T CAaAACCGTcTGGATTC GC GTCACCAACCTGAACAAC

                 *     2120         *     2140         *     2160
H4_AJ53660 : accaccaccaacctgtctgaagcgcagtcccgtattcaggacgccgactatgcgaccgaagtgtccaa :  815
H25_AGSG01 : accaccaccaacctgtctgaagcgcagtcccgtattcaggacgccgactatgcgaccgaagtgtccaa :  815
H1_AB02847 : accactaccaacctgtctgaagcgcagtcccgtattcaggacgccgactatgcgaccgaagtgtccaa : 1553
H12_AY3374 : accactaccaacctgtccgaagcgcagtcccgtattcaggacgccgactatgcgaccgaagtgtccaa : 1553
             ACCAC ACCAACCTGTCtGAAGCGCAGTCCCGTATTCAGGACGCCGACTATGCGACCGAAGTGTCCAA
```

DIFFERENT RESTRICTION SITES AND POSITIONS FOR H4 and H25 *fliC* SEQUENCES

H4> AciI_GCGG-397/-410/-452/-530/-590/-596/-614/-623/-728/ *vs* H25> AciI_GCGG-397/-410/-452/-530/-590/-596/-623/-728/
H4> Dpn1(Methylated) or DpnII or MboI or NdeII or Sau3AI-327/-641/ *vs* H25> Dpn1(Methylated) or DpnII or MboI or NdeII or Sau3AI-327/
H4> HpaII or MspI-164/-390/-549/-582/ *vs* H25> HpaII or MspI-164/-390/-549/
H4> AlwI_GATCC-640/ *vs* H25> AlwI_GATCC -No Restriction
H4> BbvI_GCAGC-129/-611/ *vs* H25> BbvI_GCAGC-129/-563/
H4> Fnu4HI or ItaI_GCAGC-129/-611/ *vs* H25> Fnu4HI or ItaI_GCAGC-129/-563/
H4> AgeI or PinAI-547/-581/ *vs* H25> AgeI or PinAI-547/
H4> BsgI_GTGCAG-608/ *vs* H25> BsgI_GTGCAG -No Restriction
H4> BsrFI or BssAI or Cfr10I_ACCGGT-547/-581/ *vs* H25> BsrFI or BssAI or Cfr10I_ACCGGT-547/
H4> NspBII_CAGCGG-611/ *vs* H25> NspBII_CAGCGG -No Restriction
H4> AspEI or Eam1105I -No Restriction *vs* H25> AspEI or Eam1105I-568/

DIFFERENT RESTRICTION SITES AND POSITIONS FOR H1 and H12 *fliC* SEQUENCES

H1> AluI-143/-556/-655/-853/-985/-1021/-1045/-1390/-1402/-1409/ *vs* H12> AluI-591/-655/-853/-985/-1021/-1039/-1045/-1153/-1390/-1402/-1409/
H1> CfoI or AspLEI or HhaI or HinP1I-101/-488/-1134/-1295/-1508/ *vs* H12> CfoI or AspLEI or HhaI or HinP1I-85/-102/-488/-506/-1134/-1295/-1508/
H1> HaeIII or BsuRI or PalI-40/-320/-505/ *vs* H12> HaeIII or BsuRI or PalI-40/-245/-320/
H1> HpaII or MspI-164/-243/-396/-671/ *vs* H12> HpaII or MspI-164/-396/-671/
H1> TaqI-221/-290/-620/-1239/-1416/ *vs* H12> TaqI-619/-1239/-1416/
H1> AsuI or Sau96I_GGTCC-504/ *vs* H12> AsuI or Sau96I_GGTCC -No Restriction
H1> BbvI_GCAGC-439/-554/-653/-704/-1043/ *vs* H12> BbvI_GCAGC-439/-653/-704/-1037/-1043/
H1> BbvI or Fnu4HI or ItaI_GCTGC-52/-455/-947/ *vs* H12> BbvI or Fnu4HI or ItaI_GCTGC-52/-455/-554/-947/-1040/
H1> DdeI or BstDEI_CTCAG-47/ *vs* H12> DdeI or BstDEI_CTCAG-47/-355/
H1> Fnu4HI or ItaI_GCAGC-439/-554/-653/-704/-1043/ *vs* H12> Fnu4HI or ItaI_GCAGC-439/-653/-704/-1037/-1043/
H1> Fnu4HI or ItaI or BbvI_GCTGC-52/-455/-947/ *vs* H12> Fnu4HI or ItaI or BbvI_GCTGC-52/-455/-554/-947/-1040/
H1> FokI_CATCC-1058/-1431/ *vs* H12> FokI_CATCC-1058/
H1> MboII_GAAGA-342/ *vs* H12> MboII_GAAGA-342/-883/
H1> MboII_TCTTC -No Restriction *vs* H12> MboII_TCTTC-1429/

```
H1> SfaNI_GATGC-451/-551/-734/-806/-938/-1187/-1241/-1349/ *vs* H12> SfaNI_GATGC-451/-551/-734/-806/-938/-944/-1187/-1241/-1349/
H1> AosI -No Restriction *vs* H12> AosI-84/
H1> AviII or FspI or MstI -No Restriction *vs* H12> AviII or FspI or MstI-84/
H1> BalI or MluNI or MscI -No Restriction *vs* H12> BalI or MluNI or MscI-243/
H1> BsmAI_GTCTCG-218/ *vs* H12> BsmAI_GTCTCG -No Restriction
H1> CfrI or EaeI_TGGCCA -No Restriction *vs* H12> CfrI or EaeI_TGGCCA-243/
H1> Eco57I_CTGAAG-95/-341/-1311/-1503/ *vs* H12> Eco57I_CTGAAG-340/-1311/
H1> HaeII_GGCGCT -No Restriction *vs* H12> HaeII_GGCGCT-504/
H1> NspBII_CAGCTG -No Restriction *vs* H12> NspBII_CAGCTG-590/-1038/
H1> PstI -No Restriction *vs* H12> PstI-1040/
H1> PvuII -No Restriction *vs* H12> PvuII-590/-1038/
H1> SfcI or BstSFI_CTGCAG -No Restriction *vs* H12> SfcI or BstSFI_CTGCAG-1040/
H1> DraII or EcoO109I_GGGACCT-503/ *vs* H12> DraII or EcoO109I_GGGACCT -No Restriction
H1> PpuMI_AGGTCCT-217/ *vs* H12> PpuMI_AGGTCCT -No Restriction
H1> SgrAI_CACCGGCG -No Restriction *vs* H12> SgrAI_CACCGGCG-882/
H1> AspEI or Eam1105I-1189/ *vs* H12> AspEI or Eam1105I-553/
```

Supplementary material Figure 3. Alignment of sequences of two pairs of genes coding for H antigen, namely H4-H25 and H1-H12, that are not differentiated with developed RFLP-PCR method with using *Rsa*I restrictions enzyme. Below alignment the restriction enzymes that can be used for differentiation (with places of digestion) are listed.

A – Achtman scheme

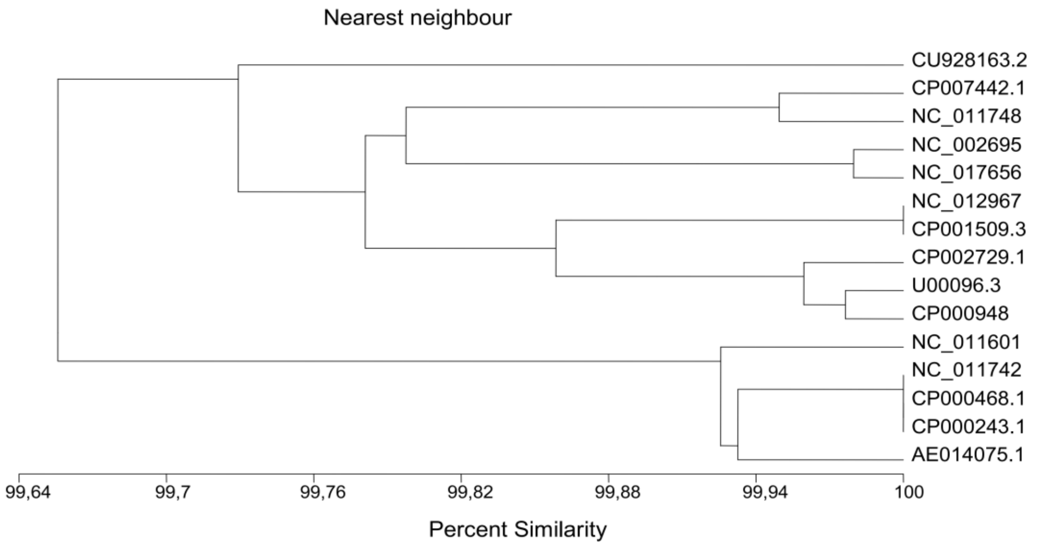

B – Pasteur scheme

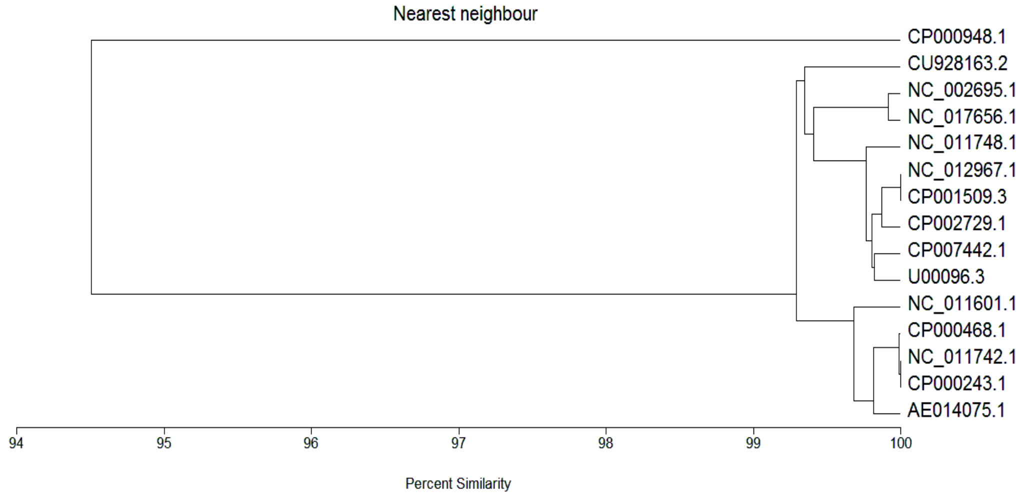

C – Clermond method

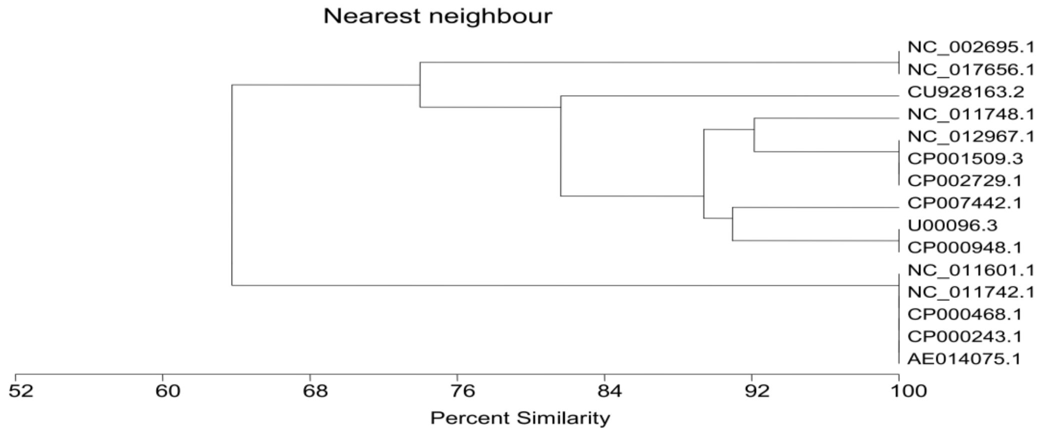

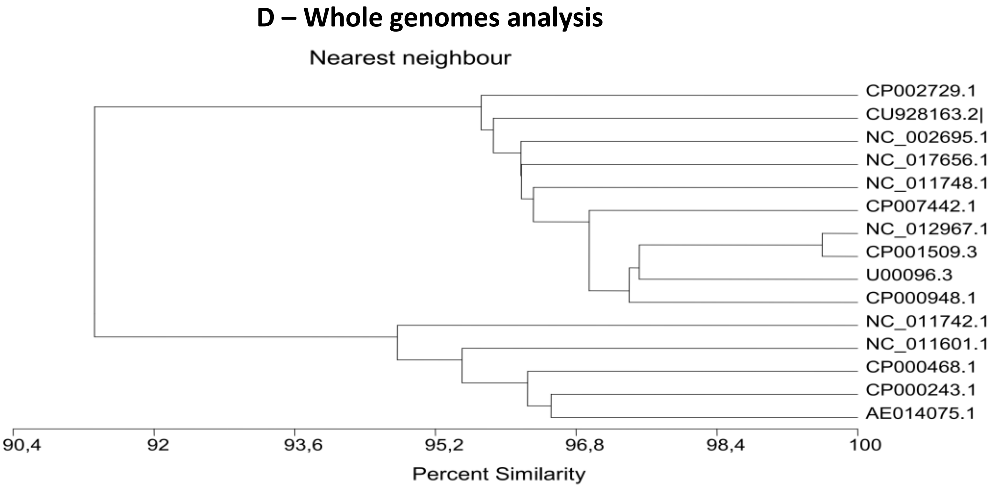

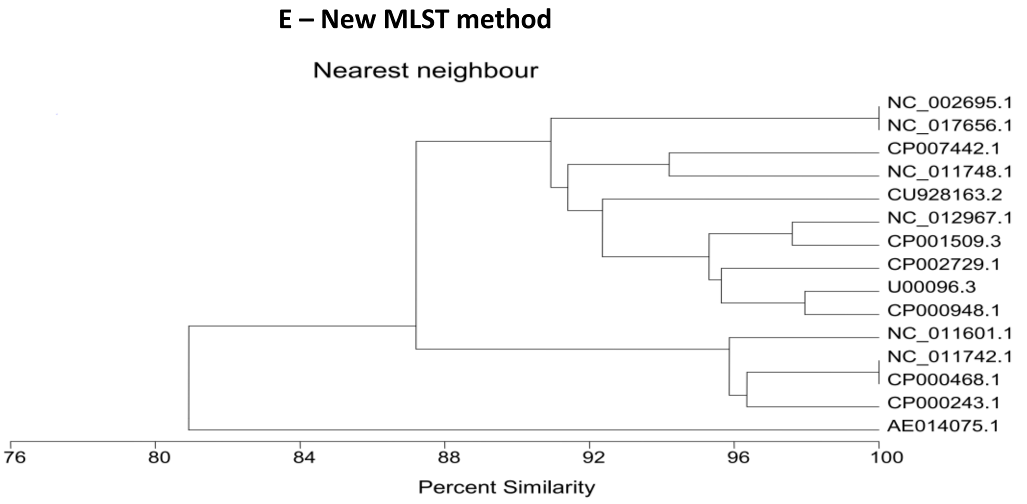

Supplementary material Figure 4. Dendrograms presenting results of *in silico* differentiation of the 15 *E. coli* strains (that were used for development of new MLST method) with four reference methods (A - Achtman scheme, B - Pasteur scheme, C - Clermond method, D – whole genome analysis) and the MLST method developed in this study (E – method developed in this study).

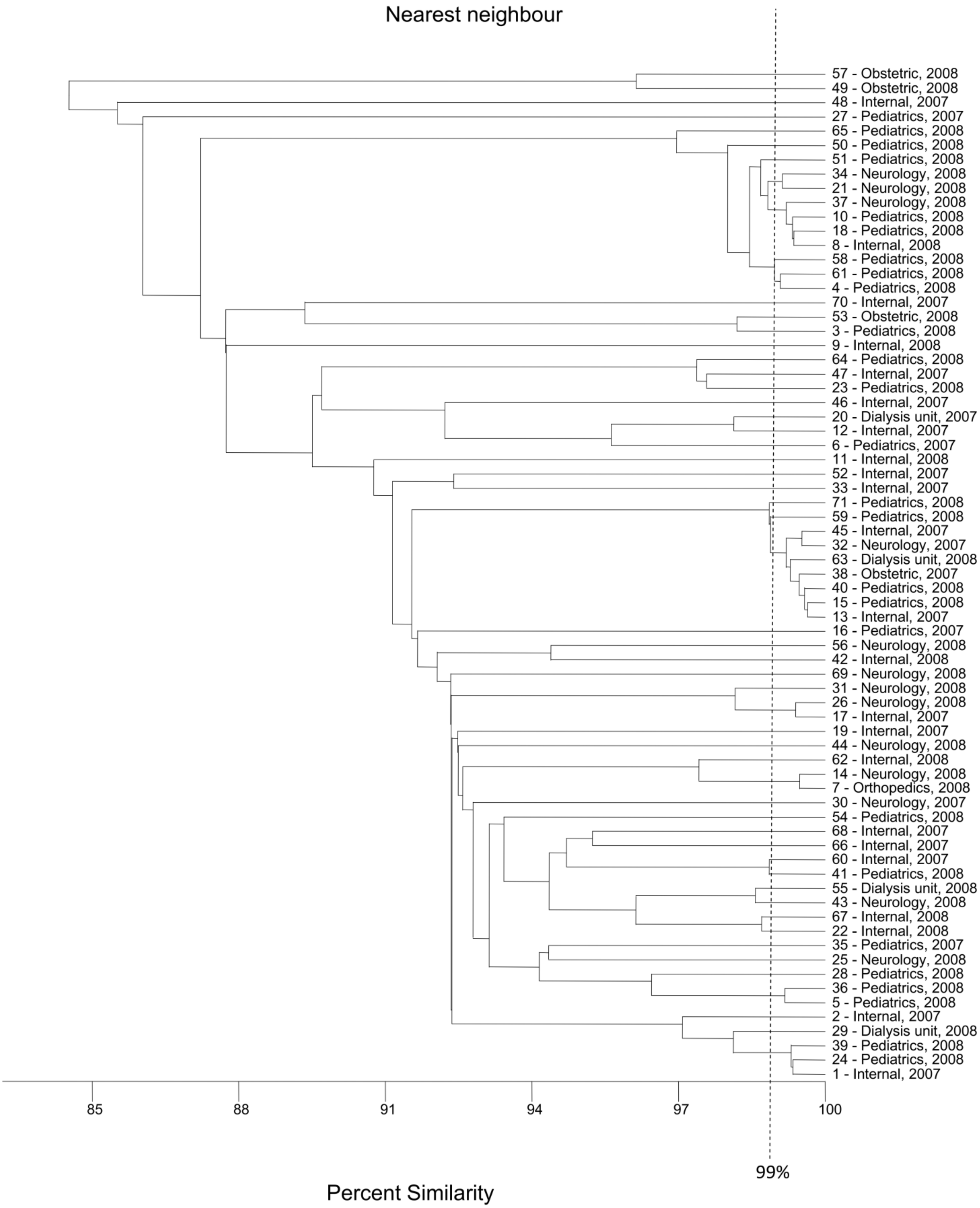

Supplementary material Figure 5. Dendrogram based on combination the results of all three methods of genotypic applied in this study (namely BOX-PCR, MLST and PCR-RFLP).

Supplementary Materials Table 1. Size of DNA bands obtained in RFLP-PCR and MLST methods for genotypes (classification according to combination of all three methods) counting more than 1 strain

Number of genotype	Number of Strains Classified to the Genotype	Numbers of Strains Classified to the Genotype	DNA fragments* in PCR-RFLP method	DNA fragments* in MLST method (fragments obtained with pairs of primers: Ec1-Ec2; Ec5-Ec6; Ec9-Ec10; Ec11-Ec12; Ec13-Ec14)
I	4	8<br>10<br>18<br>37	700, 270, 220, 155, 130	520, 220, 175, 875, 890
II	3	4<br>58<br>61	700, 270, 220, 155, 125	530, 220, 175, 750, 870
III	2	21<br>34	705, 275, 220, 160,130	435, 225, 180, 880, 935
IV	7	13<br>15<br>32<br>38<br>40<br>45<br>63	700, 275, 220, 160, 130	210, 455, 175, 1015, 385
V	3	1<br>24<br>39	575, 485, 200, 110	335, 450, 210, 1135, 385
VI	2	7<br>14	880, 170	300, 380, 320, 1000, 600
VII	2	17<br>26	890, 170	300, 380, 320, 1010, 600
VIII	2	5<br>36	500, 385, 170, 120	300, 380, 320, 1020, 385

* the size of each fragment is estimated with accuracy of +/- 10 bps.
